# Robust lipid scrambling by TMEM16 proteins requires an open groove and thin membrane

**DOI:** 10.1101/2024.09.25.615027

**Published:** 2024-09-27

**Authors:** Christina A. Stephens, Niek van Hilten, Lisa Zheng, Michael Grabe

**Affiliations:** 1 Cardiovascular Research Institute, University of California, San Francisco, CA 94158; 2 Graduate Group in Biophysics, University of California, San Francisco, CA 94158; 3 Department of Pharmaceutical Chemistry, University of California, San Francisco, CA 94158

**Keywords:** TMEM16s, scramblases, membrane deformation, MD simulation

## Abstract

Biological membranes are complex and dynamic structures with different populations of lipids on their inner and outer leaflets. The Ca^2+^-activated TMEM16 family of membrane proteins play an important role in collapsing this asymmetric lipid distribution by spontaneously, and bidirectionally, scrambling phospholipids between the two leaflets, which can initiate signaling and alter the physical properties of the membrane. While evidence shows that lipid scrambling occurs via an open hydrophilic pathway (“groove”) that spans the membrane, it remains unclear if all family members facilitate lipid movement in this manner. Here we present a comprehensive computational study of lipid scrambling by all TMEM16 members with experimentally solved structures. We performed coarse-grained molecular dynamics (MD) simulations of 27 structures from five different family members solved under activating and non-activating conditions, and we captured over 700 scrambling events in aggregate. This enabled us to directly compare scrambling rates, mechanisms, and protein-lipid interactions for fungal and mammalian TMEM16s, in both open (Ca^2+^-bound) and closed (Ca^2+^-free) conformations with statistical rigor. We show that nearly all (>90%) scrambling occurs in the dilated groove and the degree of induced membrane thinning positively correlates with the scrambling rate. Surprisingly, we also observed 60 scrambling events that occurred outside the canonical groove, over 90% of which took place at the dimer-dimer interface in mammalian TMEM16s. This new site suggests an alternative mechanism for lipid scrambling in the absence of an open groove.

## INTRODUCTION

The TMEM16 family of eukaryotic membrane proteins, also known as anoctamins (ANO), is comprised of lipid scramblases (1–3), ion channels (4–7), and members that can facilitate both lipid and ion permeation (8–15). This functional divergence, despite their high sequence conservation, is a unique feature among the 10 vertebrate paralogues (16). So far, all characterized TMEM16s require Ca^2+^ to achieve their maximum transport activity, whether that be passive ion movement or lipid flow down their electrochemical gradients (8, 11, 17–21). TMEM16s play critical roles in a variety of physiological processes including blood coagulation (8, 22–24), bone mineralization (25), mucus secretion (26), smooth muscle contraction (27) and membrane fusion (28). Mutations of TMEM16 have also been implicated in several cancers (29–31), neuronal disorder SCAR10 (32, 33), and SARS-CoV2 infection (34). Despite their significant roles in human physiology, the functional properties of most vertebrate TMEM16 paralogues remain unknown. Moreover, even though we have significant functional and structural insight into the mechanisms of a handful of members (11, 13, 15, 35–58), it is still an open question whether all TMEM16s work in the same way to conduct ions or scramble lipids.

Over the past ten years, 63 experimental structures of TMEM16s have been determined, revealing a remarkable structural similarity between mammalian and fungal members despite the diversity in their functions. All structures, except for one of fungal *Aspergillus fumigatus* TMEM16 (afTMEM16) (59) which is a monomer, are homodimers with a butterfly-like fold (12, 15, 19, 21, 46, 49, 59–67), and each subunit is comprised of 10 transmembrane (TM) helices with the final helix (TM10) forming most of the dimer interface. Residues on TM6 form half of a highly conserved Ca^2+^-binding site that accommodates up to 2 ions. TM6 along with 3, 4, and 5 also form a membrane spanning groove that contains hydrophilic residues that are shielded from the hydrophobic core of the bilayer in Ca^2+^-free states. When Ca^2+^ is bound, TM6 takes on a variety of conformational and secondary structural changes across the family, which can have profound effects on the shape of the membrane as seen in cryo-EM nanodiscs with TMEM16F (66). Ca^2+^-binding is also associated with the movement of the upper portion of TM4 away from TM6 which effectively exposes (opens) the hydrophilic groove to the bilayer, but this opening is not observed for all Ca^2+^ bound TMEM16 members (21, 46, 49, 61, 63–67).

It was first theorized (19, 68) and later predicted by molecular dynamics (MD) simulations (12, 35, 36, 40, 52, 55) that lipids can transverse the membrane bilayer by moving their headgroups along the water-filled hydrophilic groove (between TM4 and 6) while their tails project into the greasy center of the bilayer. This mechanism for scrambling, first proposed by Menon & Pomorski (69), is often referred to as the credit card model. All-atom MD (AAMD) simulations of open *Nectria haematococca* TMEM16 (nhTMEM16) have shown that lipids near the pore frequently interact with charged residues at the groove entrances (36), two of which are in the scrambling domain which confers scramblase activities to ion channel TMEM16A chimeras. Frequent headgroup interactions with residues lining the groove were also noted in atomistic simulations of open TMEM16K including two basic residues in the scrambling domain. Lipids experience a relatively low energy barrier for scrambling in open nhTMEM16 (<1 kcal/mol compared to 20–50 kcal/mol directly through the bilayer) (36, 69). Simulations also indicate that zwitterionic lipid headgroups stack in the open groove along their dipoles, which may help energetically stabilize them during scrambling (36, 42). Finally, simulations also show that lipids can directly gate nhTMEM16 groove opening and closing through interactions with their headgroups or tails (38, 39). It is important to note that all of these simulation observations are based on a limited number of spontaneous events from different groups (in aggregate we estimate up to 14 scrambling events in the absence of an applied voltage) (12, 36, 37, 40, 52, 55). Many more scrambling events (~800 in aggregate) have been seen in coarse-grained MD (CGMD) simulations for nhTMEM16 (35), TMEM16K (12), mutant TMEM16F (F518H) and even TMEM16A (70); however, a detailed analysis of how these scrambling events occurred is missing for the latter two. Moreover, a head-to-head comparison of fungal *versus* mammalian scrambling mechanisms has not been made.

An outstanding question in the field is whether scrambling requires an open groove. This question has been triggered in part by the failure to determine WT TMEM16F structures with open grooves wide enough to accommodate lipids, despite structures being solved under activating conditions (66, 67). Further uncertainty stems from data showing that scrambling can occur in the absence of Ca^2+^ when the groove is presumably closed (11–13, 19, 40, 43, 62). Moreover, afTMEM16 can scramble PEGylated lipids, which are too large for even the open groove (43). This last finding motivated Malvezzi *et al.* (43) to propose an alternate model of scrambling inspired by the realization that the bilayer adjacent to the protein, whether the groove is open or closed, is distorted based on observations from cryo-EM in nanodiscs (59–61, 66), MD simulations (12, 36, 38), and continuum models (36). The hallmark of this distortion is local bending and thinning adjacent to the groove (estimated to be 50–60% thinner than bulk for some family members) (12, 36, 38, 59–61, 66), and it has been suggested that this deformation, along with packing defects, may significantly lower the energy barrier for lipid crossing (36, 59, 66). To date no AAMD or CGMD simulation has reported scrambling by any wild-type TMEM16 harboring a closed groove; however, a CGMD simulation of the F518H TMEM16F mutant did report scrambling, but the details, such as whether groove opened, were not provided (70). Again, since a comprehensive analysis across all family members has not been carried out, it is difficult to determine how membrane thinning is related to scrambling or if scrambling mechanisms are specific to certain family members, conformational states of the protein, or both. Additionally, lipids are also directly involved in how TMEM16 scramblases conduct ions. As first speculated in ref. (11), AAMD simulations have shown that ions permeate through the lipid headgroup-lined hydrophilic groove of TMEM16K and nhTMEM16 (37, 39, 41, 42, 58). How might this mechanism differ in the absence of an open groove?

To address these outstanding questions, we employed CGMD simulation to systematically quantify scrambling in 23 experimental and 4 computationally predicted TMEM16 proteins taken from each family member that has been structurally characterized: nhTMEM16, afTMEM16, TMEM16K, TMEM16F, and TMEM16A (Table S1, Fig. S1). CGMD, which was the first computational method to identify nhTMEM16 as a scramblase (35), enables us to reach much longer timescales, while retaining enough chemical detail to faithfully reproduce experimentally verified protein-lipid interactions (71). This allowed us to quantitatively compare the scrambling statistics and mechanisms of different WT and mutant TMEM16s in both open and closed states solved under different conditions (e.g., salt concentrations, lipid and detergent environments, in the presence of modulators or activators like PIP_2_ and Ca^2+^). Our simulations successfully reproduce experimentally determined membrane deformations seen in nanodiscs across both fungal and mammalian TMEM16s. They also show that only open scramblase structures have grooves fully lined by lipids and each of these structures promote scrambling in the groove with lipids experiencing a less than 1 kT free energy barrier as they move between leaflets. One simulation of TMEM16A, which is not a scramblase, initiated from a predicted ion conductive state exhibited a very low scrambling rate facilitated by a lipid-lined groove where the lipids experienced an energy barrier 2.5–5.5 times larger than the true scramblases. Our analysis of the membrane deformation and groove conformation shows that most scrambling in the groove occurs when the membrane is thinned to a least 14 Å and the groove is open. We also observe >250 complete ion permeation events but only in the open scramblase structures and one TMEM16A structure which also had the most robust flows of water in the pathway. Our simulations also reveal alternative scrambling pathways the majority of which occur at the dimer-dimer interface in mammalian structures.

## RESULTS

### Lipid densities from coarse-grained simulations match all-atom simulations and cryo-EM nanodiscs

**Figure 1.**
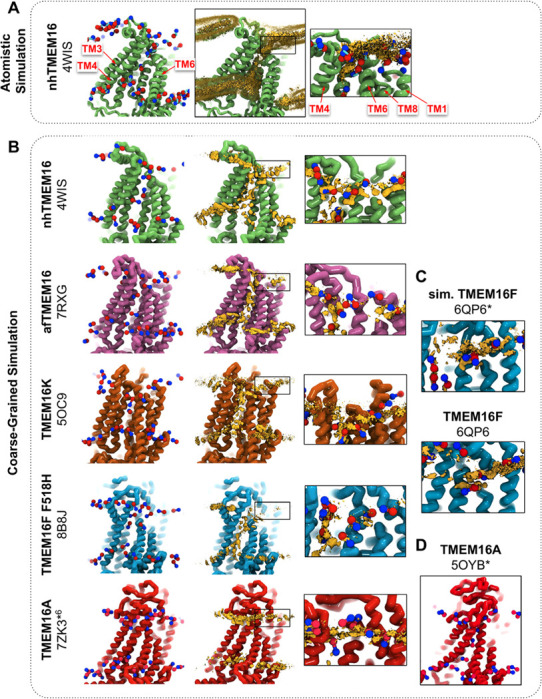
CG simulations of multiple TMEM16 structures captures lipid density in the TM4/TM6 pathway of scrambling competent members. **(A)** Snapshot and POPC headgroup density (right) from atomistic simulations of Ca^2+^-bound nhTMEM16 (PDB ID 4WIS) previously published in Bethel & Grabe 2016 (36). Only the PC lipid headgroup is shown for clarity. Density is averaged from both subunits, except TMEM16K 5OC9 which is asymmetric, across 8 independent simulations totaling ~2 μs. **(B)** Snapshots from CG simulations of open Ca^2+^-bound nhTMEM16 (PDB ID 4WIS, green), afTMEM16 (PDB ID 7RXG, violet), TMEM16K (PDB ID 5OC9, orange), TMEM16F F518H (PDB ID 8B8J, blue), TMEM16K (orange), and TMEM16A (red). **(C)** Snapshots with lipid headgroup densities around simulated open (6QP6*) and closed (PDB ID 6QP6) TMEM16F. **(D)** Snapshot of simulated ion conductive TMEM16A (5OYB*). Only the PC lipid headgroup is shown for clarity. Each density is averaged over both chains except TMEM16K and TMEM16A where only a single chain is used due to the structure’s asymmetry.

We simulated coarse-grained Ca^2+^-bound and -free (apo) structures of TMEM16 proteins in a 1,2-dioleoyl-sn-glycero-3-phosphocholine (DOPC) bilayer for 10 μs each using the Martini 3 forcefield (72). First, we determined how well the simulated membrane distortions matched experiment by comparing the annulus of lipids surrounding each protein to the lipid densities derived from structures solved in nanodiscs (Fig. S2). The shapes of the membrane near the protein qualitatively match the experimental densities and the shapes produced from AAMD simulations and continuum membrane models (36, 59, 61). For example, the CG simulations capture the sinusoidal curve around both fungal scramblases in apo and Ca^2+^-bound states (Fig. S2) previously determined by atomistic simulations (36) of Ca^2+^-bound nhTMEM16 (Fig. 1A-B). Even though membrane deformation is a feature of TMEM16s, the shapes between fungal and mammalian members are noticeably different. Specifically, the membrane appears flatter around TMEM16K and TMEM16F compared to the fungal members in both the nanodisc density and CGMD (Fig. S2). For WT TMEM16s, whether the groove is open (Fig. 1B, insets) or closed (Fig. S3), strong lipid density exists near the extracellular groove entrances at TM1 and 8. Interestingly, this density is lost in the simulation of the Ca^2+^-bound constitutively active TMEM16F F518H mutant (PDB ID 8B8J), consistent with what is seen in the cryo-EM structure solved in nanodisc (Fig. S2). The lipid density is, however, similarly strong at this location for the simulated open Ca^2+^-bound WT TMEM16F (6QP6*, initiated from PDB ID 6QP6) and closed Ca^2+^-bound WT TMEM16F (PDB ID 6QP6) (Fig. 1C). The loss of density indicates that the normal membrane contact with the protein near the TMEM16F groove has been compromised in the mutant structure. Residues in this site on nhTMEM16 and TMEM16F also seem to play a role in scrambling but the mechanism by which they do so is unclear (59, 62, 66, 67).

Headgroup density isosurfaces from CGMD simulations of known scramblases bound to Ca^2+^ and with clear separation of TM4 and 6 show that lipid headgroups occupy the full length of the groove creating a clear pathway that links the upper and lower membrane leaflets (nhTMEM16 (PDB ID 4WIS), afTMEM16 (PDB ID 7RXG), TMEM16K (PDB ID 5OC9) and TMEM16F F518H (PDB ID 8B8J), Fig. 1B). These simulation-derived densities crossing the bilayer are strikingly similar to lipids resolved in cryo-EM structurers of fungal scramblases in nanodisc (59, 62). Individual simulation snapshots provide insight into how lipids traverse this pathway. Additional analysis shows that all of the grooves are filled with water (Fig. S4). These profiles share additional features including a clear upward deflection of the membrane as it approaches TM3/TM4 from the left and a downward deflection as it approaches TM6/TM8 from the right; however, the degree of this deflection is not equal as can be seen for TMEM16K, which is less pronounced (Fig. 1B). These distortions are coupled to the sinusoidal curve around the entire protein, which was shown to thin the membrane across the groove and hypothesized to aid in scrambling (36).

Unlike the open Ca^2+^-bound scramblase structures, apo and closed Ca^2+^-bound TMEM16 structures lack lipid headgroup density spanning the bilayer, and their density profiles are more consistent across the entire family (Fig. S3). The membrane is deformed near the groove with some lower leaflet lipid density entering part of groove and some of the upper leaflet density deflecting inward around TM1, TM6, and TM8 but not entering the closed outer portion of the groove. Again, the membrane around TMEM16F and TMEM16K is flatter than it is in the fungal scramblases. Similarly, simulation of a Ca^2+^-bound TMEM16A conformation that conducts Cl^−^ in AAMD (7ZK3*^6^, initiated from PDB ID 7ZK3, see Methods and Fig. S1) samples partial lipid headgroup penetration into the extracellular vestibule formed by TM3/TM6, but lipids fail to traverse the bilayer as indicated by the lack of density in the center of the membrane (Fig. 1B). This finding is consistent with TMEM16A lacking scramblase activity (54, 73); however, there is a different ion-conductive TMEM16A conformation that can achieve a fully lipid-line groove during its simulation (Fig. 1D).

### Simulations recapitulate scrambling competence of open and closed structures

**Figure 2.**
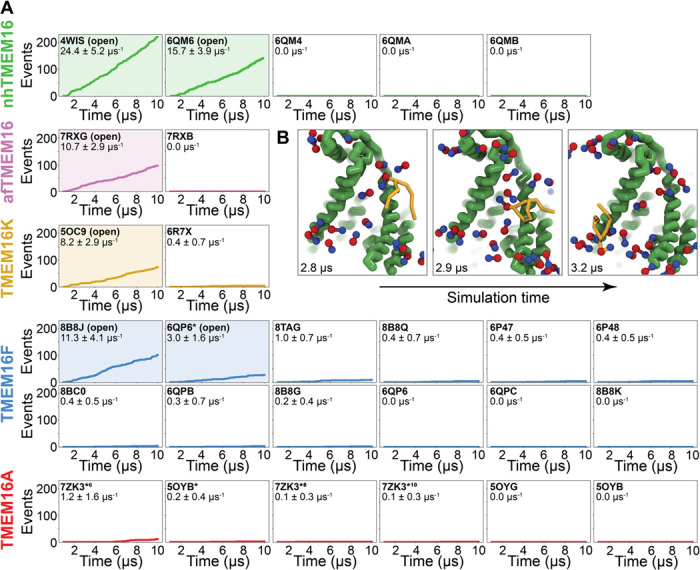
Simulated lipid scrambling differentiates closed/open conformations. **(A)** Accumulated scrambling events during CGMD simulation of experimental and simulated (sim) structures of nhTMEM16 (green), afTMEM16 (violet), TMEM16K (gold), TMEM16F (blue), TMEM16K (orange), and TMEM16A (red). Average and standard deviation values are inset in each plot. Structures that were described as open in the respective original publication (PDB IDs 4WIS (19), 6QM6 (61), 7RXG (59), 5OC9 (12), 8B8J (60), and 6QP6 (55)) are shaded in the same corresponding colors. **(B)** Snapshots of the open nhTMEM16 simulation (PDB ID 4WIS) showing a single scrambling event over time. Scrambling lipid shown with lipid tails (yellow) and headgroups of all other lipids shown as headgroup spheres only.

To quantify the scrambling competence of each simulated TMEM16 structures, we determined the number of events in which lipids transitioned from one leaflet to the other (see Methods, Fig. S5). The scrambling rates calculated from our MD trajectories are in excellent agreement with the presumed scrambling competence of each experimental structure (Fig. 2A). The strongest scrambler was the open-groove, Ca^2+^-bound fungal nhTMEM16 (PDB ID 4WIS (19)), with 24.4 ± 5.2 events per μs (Fig. S6A). In line with experimental findings (61), the open Ca^2+^-free structure (PDB ID 6QM6), which is structurally very similar to PDB ID 4WIS, also scrambled lipids in our simulations (15.7 ± 3.9 events per μs, Fig. S6B). In contrast, we observed no scrambling events for the intermediate- (PDB ID 6QMA) and closed- (PDB ID 6QM4, PDB ID 6QMB) groove nhTMEM16 structures (61). We observed a similar trend for the fungal afTMEM16 (59), where our simulations identified the open Ca^2+^-bound cryo-EM structure (PDB ID 7RXG) as scrambling competent (10.7± 2.9 events per μs, Fig. S6C) while the Ca^2+^-free closed-groove structure (PDB ID 7RXB) was not.

For TMEM16K, our simulations showed that the Ca^2+^-bound X-ray structure (PDB ID 5OC9) facilitates scrambling (8.2 ± 2.9 events per μs) in line with experiments in the presence of Ca^2+^, when the groove is presumably open, and previous MD simulations (12). Interestingly, we found a significant asymmetry in the number of scrambling events between the two different monomers, with >80% of events happening via chain B (Fig. S7A). Although both monomers are Ca^2+^-bound, chain B has a slightly wider ER lumen-facing entrance to the groove in the starting structure (Fig. S7B) and spontaneously opened its groove more than subunit A during the simulation (8.2 Å compared to 5.8 Å on average, Fig. S22), which likely accounts for the increased rate. The closed-groove TMEM16K conformation (PDB ID 6R7X) (12) showed very little scrambling activity (0.4 ± 0.7 events per μs).

Although TMEM16F is a known lipid scramblase found in the plasma membrane of platelets (10), none of the WT structures solved to date, even those determined under activating conditions, have exhibited an open hydrophilic groove. We simulated 10 of these proteins and observed little to no lipid scrambling in each case (Fig. 2A). Others have shown that mutations at position F518 turns TMEM16F into a constitutively active scramblase (52) and that the F518H mutant (8B8J) is structurally characterized by a kink in TM3, and TM4 pulls away from TM6 35° compared to a closed WT TMEM16F structure (PDB ID 6QP6) (60). In our simulations, TMEM16F F518H (PDB ID 8B8J) was the only system initiated directly from a solved structure that showed scrambling activity (11.3 ± 1.6 events per μs). Additionally, we performed a CGMD on a WT TMEM16F with a single open groove obtained from AAMD initiated from a closed-state structure (cluster 10 in (55), 6QP6* in Fig. 2A). We observed moderate lipid scrambling activity (3.0 ± 1.6 events per μs), most of which happened through the open groove (Fig. S8B-C). Although the rates of scrambling are higher for the mutant than the open WT TMEM16F, there were no noticeable differences in how lipids enter the pathway or how long they take to transition (Fig. S17).

Finally, we simulated six structures of mouse TMEM16A, which functions as an ion channel but lacks lipid scrambling activity (46). As expected, both the Ca^2+^-bound (PDB ID 5OYB) and the Ca^2+^-free (PDB ID 5OYG) experimental structures failed to induce scrambling in the CGMD simulations, as did one alternative and two ion conduction-competent structures that were obtained from AAMD (see Methods for details). However, a TMEM16A state with an open hydrophilic groove predicted by Jia & Chen (5OYB*, simulations initiated from PDB ID 5OYB) (48) did scramble a single lipid through each groove in a manner nearly identical to the scramblases (Fig. S9A, E and Video S1).

### Active scramblases thin the membrane near the groove

As we first suggested based on our simulations of nhTMEM16 (36) and now supported by additional simulation and cryo-EM (12, 39, 59, 60, 66) – membrane thinning is hypothesized to contribute to the scramblase activity of TMEM16s. To quantify the membrane deformation in our simulations, we calculated the ensemble-averaged positions of the glycerol groups of the lipids in the upper and lower membrane leaflets (see Methods for details). For most TMEM16 structures, we observed strong deformations of up to 10 Å from the equilibrium height in both leaflets close to the membrane-protein interface (Fig. S10-14 for all data). The patterns around the proteins are sinusoidal with inward deflections (orange) followed by outward deflections (purple) and the leaflet movements are tightly coupled. Nonetheless, bilayer thinning does occur adjacent to most proteins as revealed by subtracting the leaflet heights (Fig. S10-14). This visualization indicates that active scramblases indeed tend to feature more extreme membrane thinning, such as TMEM16F F518H (PDB ID 8B8J, 11.9 Å), open-groove nhTMEM16 (PDB ID 4WIS, 12.0 Å), afTMEM16 (PDB ID 7RXG, 12.2 Å), and TMEM16K (PDB ID 5OC9, 11.9 Å) as indicated by larger and deeper red areas near the hydrophilic grooves in the insets in Fig. S10-14 (e.g., PDB IDs 4WIS, 7RXG, 5OC9, and 8B8J).

### Scrambling rate depends on both groove dilation and membrane thinning

Although membrane thinning is well established for TMEM16, whether it is *sufficient* for scrambling when the groove is closed is an open question (74). Ninety-two percent of the observed scrambling events occur along TM4 and TM6 with headgroups embedded in the hydrated groove, in line with the credit card model, which we refer to as “in-the-groove” scrambling (Table 1). We previously identified four residues (E313, R432, K353, and E352) at the intracellular and extracellular entrances of the nhTMEM16 groove that we hypothesized help organize or stabilize scrambling lipids ((36), Fig. S15A, B). However, our CGMD of the same nhTMEM16 structure shows although these residues have elevated contact frequencies, more than half of the contacts are made with bulk lipids that do not end up scrambling (Fig. S15C, D). Across the family, scrambling events do not appear to enter and leave the groove at specific locations (Fig. S6-8) with roughly only 3–10% of events passing through high density lipid regions on lower TM4 and upper TM6/TM8 (Fig. 1B, Fig. S16). On average scrambling lipids do not spend more than 20 ns (< 1% the total simulation time) interacting with residues all along the groove interior, except for open TMEM16K which has higher dwell times concentrated at its groove constriction point (Fig. S17-18). Taken together this indicates that lipids do not to need to interact with specific residues near the groove entrances or within groove to scramble. Consistent with the low dwell times, for robust scramblers the free energy profile for lipids moving through the open groove is barrierless (< 1 kT) (Fig. S19A) with similar kinetics among the homologs and a mean diffusion coefficient between 10 and 16 Å^2^/𝑛𝑠 (Fig. S20). The in-the-groove scrambling events were Poisson distributed for all robust scramblers (Fig. S21), indicating that they occur independently.

**Figure 3.**
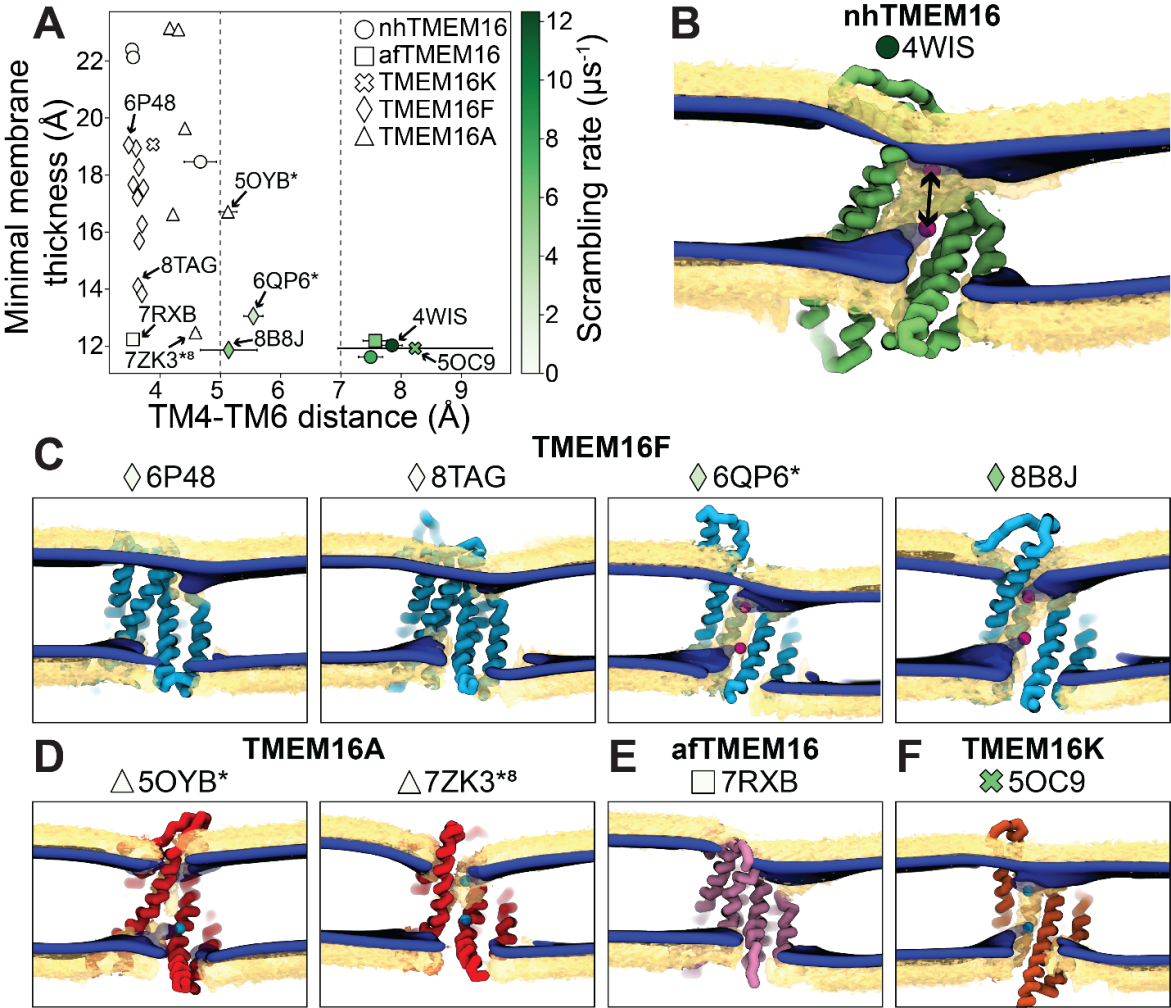
Lipid scrambling rates correlate with groove openness and membrane thinning. **(A)** The minimal membrane thickness (thinning) plotted against the average width of the groove measured by the minimal distance between any two residues on TM4 and TM6 of the groove with the most scrambling events. Each data point is colored by the scrambling rate through that same groove. Dashed lines define three regimes of groove openness, as discussed in the main text. **(B-F)** Density isosurfaces for DOPC headgroup beads (yellow) and average membrane surface calculate from glycerol beads (blue) from **(B)** nhTMEM16, **(C)** TMEM16K, **(D)** TMEM16A, **(E)** afTMEM16 and **(F)** TMEM16K simulations. Cartoon beads in each images indicate closest points between the inner and outer leaflet of the average surface. Closest points for TMEM16F PDB IDs 6P48 and 8TAG and afTMEM16 PDB ID 7RXB are located outside of the groove and not visible in this view.

To quantify how groove openness and membrane thinning relates to these events, we measured the minimum distance between residues on TM4 and TM6 at the most constricted location in the pathway (see Methods for details) and plotted it against the minimal membrane thickness (i.e., the shortest distance between the ensemble-averaged surfaces) with each data point colored by the scrambling rate in the groove (Fig. 3A). Our results show that a membrane thinning below 14 Å *and* a groove dilation above 5 Å constitute the minimal requirements for robust lipid scrambling. We further categorize this membrane thinning and protein conformation-dependent scrambling into three regimes (from right to left in Fig. 3A): widely open conformations that simultaneously thin the membrane and have no steric blockage along the groove exhibit most robust scrambling; less dilated conformations that present single or multiple steric barriers along the groove may achieve enhanced scrambling ability by leveraging structural fluctuations and/or membrane thinning; lastly, conformations with a groove dimension narrower than the width of the lipid headgroup exhibits minimal scrambling, regardless of the degree of membrane thinning.

To further illustrate the features corresponding to different levels of scrambling activity, we hereby present examples for these three regimes. Starting from the rightmost region on Fig. 3A, the most robust scramblers are characterized by membrane thinning to at least 13 Å and grooves wider than 7 Å. For example, the widely open Ca^2+^-bound nhTMEM16 structure (PDB ID 4WIS) funnels lipids directly in the pathway and lipid headgroups move in an uninterrupted fashion in the path (Video S2). Moving leftwards in Fig. 3A, scrambling activity plummets consistent with an increasingly constricted groove. Nonetheless, we presented two structures that have intermediate scrambling activity, both are Ca^2+^-bound TMEM16F (PDB ID 8B8J, 6QP6*). Upon inspection, we noticed that the hydrophobic gate at the pathway midpoint dynamically swings open to sporadically allow lipids through (Video S2-3). This resulted in a slower scrambling rate compared to the most robust scramblers. This regime of scrambling also appears to apply to the subunit that scrambles 8 times less than the more dilated opposite subunit in asymmetric TMEM16K (5OC9) (Fig. S7A, Fig. S22, Video S5-6). Uniquely, the regime of intermediate scramblers in Fig. 3A also contains a conformation that samples groove widths exceeding the 5 Å threshold for minimal scrambling, but nevertheless exhibits negligible activity (5OYB*, only 2 events). Close inspection of its trajectory reveals that although lipid headgroups can penetrate deeply into pathway, several locations restrict lipid movement (Video S1). Additionally, this TMEM16A conformation fails to thin the membrane. This notion reveals a second trend for this intermediate regime: a correlation between the membrane thinning and the scrambling rate when going down the plot in Fig. 3A from 5OYB* to 6QP6* to 8B8J.

The membrane profiles at the groove entrances look very similar between open nhTMEM16 (PDB ID 4WIS) and the scrambling-competent TMEM16F structures (6QP6* and PDB ID 8B8J) in that the upper leaflet is lowered on the TM6 side and lifted around TM4 (Fig. 3B, C). This similarity is not reflected in the scrambling rates, which suggests that the way lipids approach the groove does directly influence the protein’s scrambling competence. The membrane profiles near the groove for non-scrambling competent WT TMEM16F structures (PDB IDs 6P48 and 8TAG) are also similar to 6QP6* and PDB ID 8B8J, but they lack lipid density along the full length of the groove, and they do not scramble at the groove (Fig. 3C). We noticed that the Ca^2+^-bound fungal nhTMEM16 and afTMEM16 structures create more drastic and long-range deformations than the active mammalian TMEM16K and TMEM16F structures, which induce a sharp local thinning but leave the rest of the membrane relatively flat (Fig. 3B-F, Fig. S10-14).

### Water and ion content in the groove

**Figure 4.**
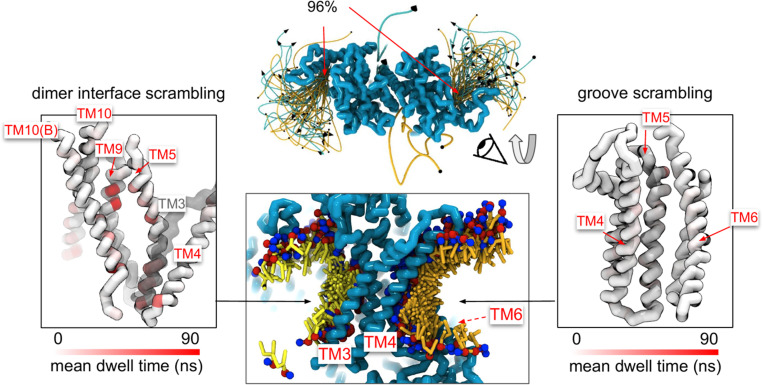
Lipid scrambling events and lipid-protein residue contact in the dimer interface and canonical TM4/TM6 groove. Traces of all scrambling lipids in a TMEM16F (PDB ID 8B8J) simulation (center top). Lipids scramble from inner to outer leaflet are illustrated as cyan traces and from outer to inner leaflet as yellow traces. A cartoon depiction of two inward individual scrambling events along the TM4/TM6 groove (orange) and the dimer interface (yellow) with multiple snapshots over time (center bottom). Only headgroup, first and second tail beads are shown for clarity. Protein backbone colored by mean lipid headgroup interaction (dwell) time at the TMEM16F dimer interface (left) and TM4/TM6 groove (right).

To quantify how hydration of the groove or pore relates to scrambling, we measured the number of water permeation events along the pathway of maximum water density at the grooves (Fig. 4A, Table S2 and Methods for details). As expected, the number of water permeation events for most closed scramblase structures was low, < 30 events per μs on average, while dilated TM4/TM6 grooves (5 out of 6 Ca^2+^-bound) support 300–550 permeation events per μs on average. Nonetheless, even when the groove is inaccessible to lipids in closed and intermediate states, including the TMEM16A ion channel path, it remains hydrated but the stream of water is shielded from the hydrophobic core of the membrane (Fig. S4A*, closed*). As the groove opens, water is exposed to the membrane core and lipid headgroups insert themselves in the water-filled groove to bridge the leaflets (Fig. S4A, *open*) as observed in fully atomistic simulations (12, 35–37, 39–42, 52, 55, 58).

We also observed spontaneous permeation of Na^+^ and Cl^−^ ions through the scramblase TMEM16 grooves and TMEM16A pore (Fig. 4B, Table S2), in line with the known ion-conducting capacity of these proteins (8–15, 41, 42). Of the fungal structures, only the scrambling competent open states sampled multiple ion permeation events with Ca^2+^-bound nhTMEM16 (PDB ID 4WIS) showing highest conductance followed by Ca^2+^-free nhTMEM16 (PDB ID 6QM6), which was 3 times lower, and then Ca^2+^-bound afTMEM16 (PDB ID 7RXG), which was another 3 times lower again. We also measured cation-to-anion selectivity ratios of 5.1, 3.2, and 6 for each simulation, respectively, computed from the ratio of total counts (P*_Na_*/P*_Cl_*). Our simulations are consistent with experiments showing that both fungal scramblases transport anions and cations (13), and both are weakly cation selective (P*_K_*/P*_Cl_* = 1.5 for afTMEM16 based on experiment (11) and P*_Na_*/P*_Cl_* = 8.7 for nhTMEM16 based on AAMD (42)). Our CGMD simulations also sample ion conduction through open Ca^2+^-bound TMEM16F F518H (PDB ID 8B8J, P*_Na_*/P*_Cl_* = 1.3), which had the most ion permeation events (102) across the family, simulated open TMEM16F (6QP6*, P*_Na_*/P*_Cl_* = 0.33), and open TMEM16K (PDB ID 5OC9, P*_Na_*/P*_Cl_* = 1.8). TMEM16K was experimentally shown to have a slight cation preference (12), while results for TMEM16F are at odds with some showing cation selectivity (P*_Na_*/P*_Cl_* = 6.8 (8) or P*_Na_*/P*_Cl_* = 2.3–4.8 depends on [Ca^2+^] (75)) while others show anion selectivity (76).

Finally, TMEM16A (7ZK3*^8^) had 4 Cl^−^ and no Na^+^ permeation events, consistent with its experimentally measured anion selectivity (P*_Na_*/P*_Cl_* = 0.1 (45)). Interestingly, we did not observe Cl^−^ permeation in any of the other computationally predicted TMEM16A structures (5OYB*, 7ZK3*^8^ and 7ZK3*^10^), while AAMD simulations of these structures all reported Cl^−^ conduction (48).

### Scrambling also occurs out-of-the-groove

A minority of our observed scrambling events (8%) occurred outside of the hydrophilic groove between TM4 and TM6. Surprisingly, most of these events happened at the dimer interface with lipids inserting their headgroups into the cavity outlined by TM3 and TM10 (Fig. 4, Fig. S6-9). We only observed scrambling at this location in simulations of the mammalian homologs. In atomistic simulations of a closed Ca^2+^-bound TMEM16F (PDB ID 6QP6), we observed a similar flipping event for a POPC lipid into the dimer interface (Fig. S23). Although the dimer interface is largely hydrophobic, there are a few polar and charged residues in the cavity near the membrane core and water is present in the lower half of the cavity (Fig. S24). In fact, the headgroup of the lipid in our atomistic simulation of TMEM16F interacts with a glutamate (E843) and lysine (K850) on TM10 near the membrane midplane (Fig. S23). Lipids that scramble at the dimer interface tend to interact for up to 10-fold longer periods of time on average than those in the canonical groove (Fig. 4). The most prolonged interactions occur at sites containing aromatic residues into which the lipid tails intercalate (Fig. S25).

There were five more out-of-the-groove events including one that occurred across a closed TM4/TM6 groove of Ca^2+^-bound TMEM16F (PDB ID 6P47). From all our observed scrambling events, this is the only one that fits the postulated out-of-the-groove definition where scrambling is expected to take place near TM4/TM6 but without inserting into the groove (77) (Fig. S26A). Two events occurred concurrently along TM6 and TM8 again near the hydrophilic groove of a Ca^2+^-bound closed TMEM16F (PDB ID 8TAG) (Fig. S26B). Lastly, two events occurred along TM3 and TM4, near the canonical TM4/TM6 groove of an open nhTMEM16 (PDB ID 4WIS) and pore of predicted conductive state of TMEM16A (7ZK3*^8^) (Fig. S26C, D). In each of these five out-of-the-groove events, the scrambling lipid transverses with 2–4 water molecules around its headgroup.

## DISCUSSION

Previous all-atom simulations of TMEM16 have captured partial translocations or – at most – a handful of complete scrambling events (e.g., (36, 40, 55)) due to the challenges inherent in simulating molecular events on the low microseconds time scale. Although these small number of AAMD-derived scrambling events yielded key insights into specific protein-lipid interactions and scrambling pathways, they cannot provide rigorous statistics on scrambling rates, nor can they be leveraged to perform a large high-throughput comparison between the various family members. To circumvent sampling issues, we used CGMD to systematically quantify lipid scrambling by five TMEM16 family members and relate their scrambling competence to their structural characteristics and ability to distort the membrane. Our simulations correctly differentiate between open and closed conformations across the five family members, consistent with a recent study (78) that showed good agreement between *in vitro* and *in silico* lipid scrambling using the same Martini 3 force field on a diverse set of proteins, including some TMEM16s (78). In addition to lipid scrambling ability, our results are in accord with the general finding that TMEM16s show very little to no ion selectivity, although permittivity ratios are predicted to vary depending on ion concentrations and lipid environments (42). Because the simulation conditions and system setups were identical in all our simulations, we are in a unique position to directly compare a host of biophysical properties between different TMEM16 family members and their structures to answer ongoing questions in the field.

Our results show that although there is a positive correlation between membrane thinning and scrambling (Fig. 3A), it is not the only basis for scrambling. For example, our simulations show that two structures: closed Ca^2+^-free afTMEM16 (PDB ID 7RXB) and simulated ion-conductive Ca^2+^ -bound TMEM16A (7ZK3*^8^), thin the membrane as much as the Ca^2+^-bound afTMEM16 structure (PDB ID 7RXG), which has the second highest scrambling rate (Fig. 2A), but neither of them scrambles lipids out of the groove (Table 1, Fig. S8). However, unlike the open afTMEM16 simulation (PDB ID 7RXG), the closed structure thins the membrane the most outside of the groove in the dimer interface, and we do not observe scrambling there. Most notably, both of these structures have closed grooves with TM4/TM6 separation less than 5 Å. In fact, we *only* saw in-the-groove scrambling events when the groove was wider than 5 Å on average. However, once the groove is sufficiently wide to permit lipids, we see increased scrambling for proteins that thin the membrane to a larger extent, as evident from the trend between 5OYB*, 6QP6*, and PDB ID 8B8J (Fig. 3A). This is in line with the notion that scrambling rates have experimentally been determined to be higher in thinner membranes (12, 59). Additionally, open fungal TMEM16s (PDB IDs 4WIS, 6QM6 and 7RXG) and mammalian TMEM16K (PDB ID 5OC9) structures thin the membrane to similar levels as PDB ID 8B8J (~12 Å) and achieve similar or higher scrambling rates but have 1.5-fold wider grooves than PDB ID 8B8J. This indicates that once the groove is sufficiently open, the exact width has less influence over the scrambling rate than the membrane deformation. We have thus determined that a key feature that accounts for most scrambling events is the groove being wide enough to allow lipid headgroups to insert into the water-filled cavity, but that the degree of membrane thinning can additionally tune the rate of scrambling.

Of the scrambling competent TMEM16 structures, the open groove nhTMEM16 (PDB ID 4WIS) is the fastest with a scrambling rate double the other homologs (Fig. 2–3). Yet on average its groove width and membrane thinning are similar (with 1–2 Å) to the other robust scramblers nhTMEM16 (PDB ID 6QM6), afTMEM16 (PDB ID 7RXG), and TMEM16K (PDB ID 5OC9) (Fig. 3). This suggest that there are other features that impact the rates, e.g., the shape of the distortion and residues lining the groove. Another feature we have not explored are mixed membranes and membranes of shorter or longer chain length. TMEM16K resides in the endoplasmic reticulum (ER) membrane which is thinner than the plasma membrane (12, 33, 79), and TMEM16K scrambling rates increase tenfold in thinner membranes (12), which may be related to our finding that the rates are fastest for proteins that induce greater thinning.

Experimental scrambling assays performed by different groups have reported basal level scramblase activity in the absence of Ca^2+^ for fungal and mammalian dual-function scramblases (11–13, 19, 40, 43, 57). It is unknown where closed-groove scrambling takes place on the protein (62) and simulations have never reported such events despite Li and co-workers reporting scrambling events from simulations initiated from closed TMEM16A, TMEM16K, and TMEM16F (78), which may have also sampled them. In aggregate, we observed 60 scrambling events that do not follow the credit-card model and occur “out-of-the-groove” (Table 1). Nearly all these events (56/60) happen at the dimer interface between TM3 and TM10 of the opposite subunit, here on referred to as the dimer cleft. Curiously, we do not observe scrambling at this location for any of the fungal structures. Although mammalian TMEM16s have a ~4–5 Å wider gap on average at the lower leaflet dimer cleft entrance than the open fungal TMEM16s, we do not always observe scrambling at such distances and sometimes do not observe any scrambling when the cleft is at its widest (Fig. S27). For all structures we see lipids from both leaflets intercalate between TM3 and TM10 (Fig. S24), which is consistent with lipid densities in cryo-EM nanodiscs images of fungal TMEM16s (59, 62) and TMEM16F (66). Based on our simulations, this interface may be a source for Ca^2+^-independent scrambling (78)

It is unclear whether the out-of-the-groove events we have observed reflect the same closed-groove scrambling activity seen in experimental assays (40, 43, 59, 62). One way to assess this is to ask whether the relative scrambling rates observed in +/− Ca^2+^ are similar to the relative rates from our simulation with open/closed hydrophilic grooves. Feng *et al.*(62) reported a 7–18 fold increase in scrambling rate by nhTMEM16 in the presence of Ca^2+^ compared to Ca^2+^-free conditions (62). Based on our open groove count of 220, we would expect 12–30 events for the closed groove states, but we observe no scrambling and no strong lipid headgroup density at the groove (Table S2, Fig. S3). Watanabe *et al.* (57) reported a 6–7 fold increase in scrambling rate by TMEM16F in the presence of Ca^2+^ compared to Ca^2+^-free conditions. The 4 and 3 events we observed for the closed Ca^2+^-free TMEM16F structures (PBD IDs 6P47 and 6QPB, respectively) in comparison to the 27 events observed for WT simulated open Ca^2+^-bound TMEM16F (6QP6*) corroborate well with this experimental data. It is possible that out-of-the-groove scrambling is highly dependent on the membrane composition, as discussed earlier, and the scrambling ratios we observe in DOPC may be different than the experimental rates determined in different lipids. This cannot be addressed without additional studies. That said, we are encouraged by the high level correspondence – we observe much higher scrambling rates through the open grooves and much smaller flipping rates elsewhere on the protein or with closed groove structures, suggesting that our simulations may be revealing aspects of Ca^2+^-independent scrambling.

With regard to predicting rates, it is a challenge to quantitatively compare CGMD scrambling rates to experiment, which tend to be 2–3 orders of magnitude slower. For example, single-molecule analysis yielded a scrambling rate of 0.04 events per μs for TMEM16F (57), whereas we find 3 and 11.3 events per μs for our scrambling-competent TMEM16F structures 6QP6* and PDB ID 8B8J, respectively. We will highlight three potential explanations for such discrepancy. First, it is well established that the Martini model increases diffusion dynamics by a factor ~4 due to the lower friction between CG beads and reduced configurational entropy compared to more chemically accurate representations (80). Second, the energy barrier for a PC headgroup to traverse the DOPC bilayer in absence of protein is reduced in Martini 3 compared to Martini 2 and AAMD (81). It is not trivial to predict how this behavior affects protein-mediated lipid scrambling, but it is likely to increase observed flipping rates. Last, the Martini 3 elastic network used to restrain the protein back bone in our simulations allows a small degree of flexibility during simulations, which may increase scrambling. For instance, the groove of the open nhTMEM16 structure 4WIS enlarges by ~3 Å during our Martini 3 simulations compared to the starting experimental structure and our Martini 2 simulations, and this dilation correlates with greater scrambling (Fig. S28A-B). We also analyzed previously published CHARMM36 AAMD trajectories starting from the same structure (36) and observed that these simulations generally stay closer to the experimental structure, but also visit dilated conformations similar to our Martini 3 results (Fig. S28B). In addition to the open nhTMEM16 structure, we observed similar subtle movements in the TM4 helix for open Ca^2+^-bound structures of afTMEM16, TMEM16K, TMEM16F, and TMEM16A that appear to enlarge the TM4/TM6 outer vestibule (Fig. S22). Others have reported that AAMD simulations sample spontaneous dilation of the groove/pore to confer either scramblase activity for WT (52, 55) and mutant (58) TMEM16F or ion channel activity for TMEM16A structures (47, 50, 82). These movements away from the experimentally solved structures may be due to the inaccuracy of our atomistic and CG forcefields or differences in the model and experimental membrane/detergent environments but more work would be needed to assess whether these dilations reflect physiologically relevant conformational states. CG simulations of closed-groove structures lack such dilations, because the backbones of TM4 and TM6 are in close enough proximity to each other (< 10 Å) to be connected by the elastic network that the Martini model requires to maintain proper secondary and tertiary structure (e.g., PDB ID 6QM4, see Fig. S28C-D). The recent GōMartini 3 model (83) in which the harmonic bonds of the elastic network (V(r)~r^2^) are replaced by Lennard-Jones potentials V(r) → 0 for large r), may be explored in future simulations to allow sampling of both closed and open configurations starting from the same structure (Fig. S28E).

Finally, we end by discussing the observed in- and out-of-the-groove scrambling for the putative ion conducting states of TMEM16A. The total number of events (11 for the highest and 1 for the lowest) was so low that our simulations may still be consistent with the lack of scramblase activity detected in experiments (54, 73), for all of the reasons discussed in the last paragraph. In fact, the energy barrier for lipids in TMEM16A groove is up to 5.5-fold larger than for the scramblases (Fig. S19B). In simulations of our three predicted conductive states of TMEM16A (7ZK3*^6,8^ and ^10^) lipid headgroups insert into the lower and upper vestibule of the pore. Compared to the inhibitor bound structured (PDB ID 7ZK3), the outer vestibule of these conductive states is notably more dilated. We observe 4 Cl^−^ permeation events by 7ZK3*^8^ which demonstrates that lipids partially line the ion conduction pathway. Surprisingly, our simulation of the predicted TMEM16A conductive states from Jia & Chen (48) did at times feature a fully-lipid lined groove, similar to how they form the proteolipidic pore in dual-function members (Fig. 1D Fig. S15, Video S1); however, we did not observe any ion permeation events from this configuration, which may be a consequence of the configuration not being physiologically relevant, the Martini 3 forcefield not being ideal for Cl^−^/lipid/protein interactions, or something else. It is intriguing that while TMEM16A has lost experimentally discernable scrambling activity, it still deforms and thins the membrane (Fig. 3A). Coupled with our observation that groove widening allows lipids to enter, we wonder if it retains thinning capabilities to facilitate partial lipid insertion to promote Cl^−^ permeation. This hypothesis has been stated before (84), and structural evidence for this proteolipidic ion channel pore has recently been reported for the OSCA1.2 mechanosensitive ion channel (85), which adopts the TMEM16 fold (86), yet it does not scramble lipids (51).

## MATERIALS and METHODS

### Starting structure selection

We simulated 23 out of the 62 cryo-EM and X-ray TMEM16 homodimer structures available at the time (Table S1). We chose not to include structures with greater than 4 Å global resolution, apart from TMEM16A PDB ID 5OYG which is the only TMEM16A apo representative and structures with either large terminus truncations (PDB IDs 6BGI, 6BGJ, 8BC1) or largely unmodelled C-terminus domains (PDB ID 6QPI). We further narrowed our final set of structures by selecting the higher resolution of structures sharing similar backbone conformations and number of Ca^2+^ ions bound (i.e. PDB ID: 6QM9 and PDB ID: 6QM5 to PDB ID: 4WIS, PDB ID: 6OY3 to PDB ID: 6QMA, PDB ID: 6R65 to PDB ID: 5OC9, PDB ID: 7RX3, PDB ID: 7RXA, and PDB ID: 6DZ7 to PDB ID: 7RXB, PDB ID: 7RX2, PDB ID: 7RWJ and PDB ID: 6E0H, and PDB ID: 6E1O to PDBID: 7RXG, PDB ID: 8B8M chain B to PDB ID: 8B8J, PDB ID: 8SUR, PDB ID: 8SUN, PDB ID: 8TAI and 8 PDB ID: 8TAL to PDB ID: 8TAG, PDB ID: 6P49 to PDB ID: 6P48, PDB ID: 7B5C and PDB ID: 7B5E to PDBID: 5OYB, PDB ID: 7B5D to PDB ID: 5OYG). One structure from TMEM16A (PDB ID 8QZC) and TMEM16F (PDB ID 6P46) were excluded because they share a similar conformation to other structures of the same homolog but differ in the number of bound Ca^2+^ ions at the orthosteric site and by a slight elevation the TM6 C-terminus. In total, we selected 5 nhTMEM16, 2 afTMEM16, 2 TMEM16K, and 11 TMEM16F dimers. We also include 2 cryo-EM structures of TMEM16A. We also chose to simulate a computationally predicted scrambling competent TMEM16F structure based on 6QP6 (55) which has a dilated groove similar to nhTMEM16, afTMEM16 and TMEM16K. We simulated several computationally predicted conductive states of TMEM16A: one based on Ca2+-bound TMEM16A (PDB ID 5OYB (48)) and three based on simulations of 1PBC-bound TMEM16A (PDB ID 7ZK3) after removing 1PBC from the pore (unpublished work) which have significant changes to TM3 and TM4 compared to their experimentally determined starting structures. Several new nhTMEM16 structures were recently published by Feng *et al*. (62) but not released until after completion of our simulation work and therefore not included here.

### Coarse-grained system preparation and simulation details

For each simulated structure, missing loops with less than 16 residues were modeled using the loop building and refinement procedures MODELLER (version 10.2 (86)). Further details on which loops were included are in Table S1. For each stretch of *N* missing residues 10x*N* models were generated. We then manually assessed the 10 lowest DOPE scoring predictions and selected the most biochemically/structurally? reasonable model. Models were inserted symmetrically into the original experimental dimer structure except for PDB IDs 8BC0, 8TAG, and 5OC9 which were published as asymmetric structures.

Setup of the coarse-grained (CG) simulation systems was automated in a python wrapper adapted from MemProtMD (35). After preparing the atomistic structure using pdb2pqr (87), the script predicted protein orientation with respect to a membrane with memembed (88). Then, martinize2 (89) was employed to build a Martini 3 CG protein model. Secondary structure elements were predicted by DSSP (90) and their inter- and intra-orientations within a 5–10 Å distance were constrained by an elastic network with a 500 kJ mol^−1^ nm^−2^ force constant (unless specified otherwise). CG Ca^2+^ ions (bead type “SD” in Martini 3) were inserted at their respective positions based on the experimental protein structure and connected to coordinating (<= 6 Å) Asp and/or Glu side chains by a harmonic bond with a 100 kJ mol^−1^ nm^−2^ force constant. A DOPC membrane was built around the CG protein structure using *insane* (91) in a solvated box of 220×220×180 Å^3^, with 150 mM NaCl. Systems were charge-neutralized by adding Cl^−^ or Na^+^ ions. For each system, energy minimization and a 2 ns NPT equilibration were performed. All systems were simulated for 10 μs in the production phase and the first microsecond was excluded from all analyses for equilibration.

All CG molecular dynamics simulations were performed with Gromacs (version 2020.6 (92)) and the Martini 3 force field (version 3.0.0 (72)). A 20 fs time step was used. Reaction-field electrostatics and Van der Waals potentials were cut-off at 1.1 nm (93). As recommended by Kim *et al.* (94), the neighbor list was updated every 20 steps using the Verlet scheme with a 1.35 nm cut-off distance. Temperature was kept at 310 K using the velocity rescaling (95) thermostat (τ_T_=1 ps). The pressure of the system was semi-isotropically coupled to a 1 bar reference pressure by the Parrinello-Rahman (96) barostat (τ_P_=12 ps, compressibility=3×10^−4^).

### Atomistic simulation details

TMEM16A atomistic simulations were initiated from a Ca^2+^/1PBC-bound structure (PDB ID: 7ZK3) after removal of the inhibitor. The missing residues 260–266, 467–482, 526–527, 669–682 were built and refined using MODELLER (version 10.2 (86)), see more details above. Simulations were performed with Gromacs (version 2020.6 (92)) and the CHARMM36 (97) and CHARMM36m (98) force fields for lipids and protein respectively. PROPKA3 was used to check the protonation state of protein residues. E624 and D405 are both weakly protonatable at neutral pH, but likely well solvated and therefore left in their negatively charged states (99). The protein was embedded in a 155×155 Å^2^ POPC bilayer and solvated in 150 mM KCl and the CHARMM TIP3P water model using CHARMM-GUI’s Membrane Builder (100). System charges were neutralized using the same ions. During minimization, equilibration, and production distance restraints with 418.4 kJ mol^−1^ nm^−2^ force constants were applied between the Cɑ atoms of residues 465 and 489, 454 and 566, 169 and 278, 126 and 176, 196 and 189, 123 and 282, 185 and 200 to stabilize the cytosolic domain. Simulations were run using a 2 fs time step in an NPT ensemble. Temperature was kept at 303.15 K using the Nosé-Hoover (101) thermostat (τ_T_=1 ps). The pressure of the system was semi-isotropically coupled to a 1 bar reference pressure by the Berendsen (102) and Parrinello-Rahman (96) barostat (τ_P_=5 ps, compressibility=4.5×10^−5^) for equilibration and production respectively. All bonds to H were constrained by the LINCS algorithm (103). Particle mesh Ewald (104)-calculated electrostatic and Van der Waals (VdW) interactions were cut off at 1.2 nm. A Verlet cut-off scheme was used for non-bonded interactions. VdW interactions were smoothly switched to zero between 1.0 and 1.2 nm. The protein with its bound Ca^2+^ ions, the membrane, and solvent bath were treated as separate groups for the thermostat coupling and center of mass removal. Harmonic restraints of the protein backbone, sidechains, lipids and dihedrals were applied and slowly reduced over 8 equilibration steps totaling ~32 ns. The equilibrated box size was ~143×143×148 Å^3^. An identical simulation protocol was used for our atomistic simulations of TMEM16F (6QP6) but used Gromacs version 2018.7. Missing residues 84–88, 143–206, 225–228, 428–444, 490–505, 588–590, 641–644, and 792–794 were modeled using MODELLER (version 10.2 (86)). Simulation details for TMEM16A performed by the Chen group are detailed in Jia & Chen 2021. Simulation details for atomistic simulations of TMEM16F (6QP6) performed by the Weinstein group are detailed in Khelashvili *et al.* 2022 (55). Simulation details for atomistic simulations for nhTMEM16 are detailed in Bethel & Grabe 2016 (36).

### Simulated TMEM16A structure selection

TM4/5/6 residue pair distances from aggregate atomistic trajectories of TMEM16A were submitted to time-independent component analysis (tICA) (105) and subsequent K-medoids clustering. 100 clusters were then used to construct a Markov-state model and microstates were grouped into macrostates using the improved Perron-cluster cluster analysis (PCCA+) method (106, 107). The above analysis was performed using MSMBuilder (108). We selected the medoids from three of these macrostates (cluster 6, 8, and 10) which are more dilated that the starting structure and predicted to represent different conductive states (Fig. S1).

### Density & maximum density path calculations

First, each protein subunit was individually aligned in x, y and z to their starting coordinates. Atomistic simulations were filtered for trajectory frames with T333-Y439 Cɑ distance >15 Å giving a total of ~2085 ns of aggregate simulation time. Then the positions of all PC headgroup beads was tracked overtime and binned in a 100×100×150 Å grid with 0.5 Å spacing centered on two residues near the membrane midplane on TM4 and TM6 using a custom script that includes MDAnalysis methods (109, 110). Density for water beads was calculated in the same way. Density in each cell was then averaged from each chain and for atomistic simulations averaged from all 8 independent simulations. One-dimensional paths through the density were calculated by first selecting a single grid cell near the center of the box, totaling the density of all cells within a 4.7 Å cutoff and then repeating this step for each cell within a 9.4 Å of the first, until a maximum density total is identified. We then saved the centroid of this final set of grid cells and repeated the search using this centroid, or node, as the starting position. This process was continued until either the path length (sum of distances between subsequent nodes) reached 80 Å for lipids (30 Å for water), no new nodes were found, or the next selected node caused the path to deviate sharply (<90°). We only included cells with densities >=0.0005 for lipids or >=0.002 for water for this calculation. Alternative paths were calculated by repeating the search but excluding cells used to define the nodes of the maximum density path. Path nodes where then interpolated using a B-spline representation (111) and final nodes were selected from this path to given 0.5 Å gaps between nodes for the lipid paths (4.7 Å for water).

### Water permeation analysis

For each simulation the position of water CG beads within 9.4 Å search radius of the maximum density pathway were tracked overtime using a custom script that includes MDAnalysis methods (109, 110). Water beads were assigned to path nodes if that fell within the bounds of a cylindrical disc (4.7 Å height, 9.4 Å diameter) centered on the bead with its face normal defined by the vector between the current and subsequent node. Permeation events were counted if a water bead left the search radius, and its last bin assignment was in the opposite half of nodes as when it entered the path. The maximum density path was mirrored symmetrically on both subunits and each pathway was tracked independently. We also calculated the flux of water between nodes by counting the net number of waters entering and leaving a cylinder (of the same proportions above) centered on each path node at each 1 ns timestep. The residues chosen for measuring the distance between TM4 and TM6 were located within ~6 Å in z (1–2 𝛼-helix turns) of the path node with the minimum net flux of water. The residues used for each homolog were as follows: 327–339 and 430–452 for nhTMEM16, 319–331 and 426–438 for afTMEM16, 365–377 and 434–446 for TMEM16K, 512–424 and 613–625 for TMEM16F, and 541–553 and 635–647 for TMEM16A.

### Scrambling analysis

Lipid scrambling was analyzed as described by Li *et al.* (70). For every simulation frame (1 ns sampling rate), the angle between each individual DOPC lipid and the z-axis was calculated using the average of the vectors between the choline (NC3) bead and the two last tail beads (C4A and C4B), see Fig. S5A. We applied a 100 ns running average to denoise the angle traces. Lipids that reside in the upper leaflet are characterized by a 150° angle, and lipids in the lower leaflet have a 30° angle. Scrambling events were counted when a lipid from the upper leaflet passed the lower threshold at 35° or, *vice versa*, when a lipid from the lower leaflet passed the upper threshold at 145° (see Fig. S5B). These settings are more stringent than the thresholds used by Li *et al.* (55° and 125°, respectively) to prevent falsely counted partial transitions (70). A 1 μs block averaging was applied to obtain averages and standard deviations for the scrambling rates.

Diffusion coefficients for lipids were calculated by first making a 3D interpolated path for each scrambling event and then measuring the minimum path distance to the starting position at each time point. We used a linear least squared regression to fit the slope of each squared displacement curve (squared minimum path distance) and divided the slope by 2 (for 1D diffusion) to obtain the final diffusion coefficient for each lipid. We wrote custom scripts for this analysis using MDAnalysis (109, 110) and Scipy methods (111).

### Quantification of membrane deformations

First, using Gromacs (gmx trjconv), MD trajectories were aligned in the xy-plane such that the longest principal axis defined by the initial positions of TM7 and TM8 aligned to the global y axis. Average membrane surfaces were calculated from the aligned MD trajectories as outlined previously (36) using a custom python script based on MDAnalysis (109) and SciPy (111). The positions of each lipid’s glycerol beads (GL1 and GL2) were linearly interpolated to a rectilinear grid with 1 Å spacing. Averaging over all time frames (again, discarding the first 1 μs for equilibration) yielded a representative upper and lower leaflet surface. Grid points with a lipid occupancy below 2% were discarded. The minimal membrane thickness was calculated as the minimal distance between any two points on the opposing ensemble-averaged surfaces (e.g., Fig. 3B). Crucially, in the case of lipid scrambling simulations like the ones described here, lipids were assigned to the upper/lower leaflet separately for every time frame.

### Protein-lipid contact and dwell time analysis

Using the full 10 μs simulation where each protein subunit was individually aligned in x, y, and z, we analyzed protein-lipid interactions by measuring distances between the protein’s outermost sidechain bead (except for glycine, which only has backbone bead) and the lipid’s choline (NC3) or phosphate (PO4) bead for every nanosecond using custom scripts with Scipy methods (111). Contacts were defined as distances below 7 Å. Contact frequency was calculated as the fraction of simulation frames where a contact occurred, averaged over two monomers. Dwell time was measured as the duration of consecutive contacts, allowing breaks up to 6 ns to account for transient fluctuations of lipid configuration. For each residue, we selected either the choline or phosphate bead based on which yielded the higher average dwell time. To visualize the result, we used averaged dwell time of the top 50% longest dwelling events at each residue to generate a color-coded representation of the protein structure (Fig. 4 and Fig S17).

### PMF calculations

To calculate the PMFs for each pathway, we assigned each cell from the average PC density to its closest respective mean-lipid pathway node. We then summed the density of all cells assigned to a given node and divided by the total volume of those cells. We used the following equation to convert the density values to energies or PMF.


$$E=\frac{-R*T*\ln(\rho /\rho_{0})}{1000}$$

P_0_, is the density of PC in a 10 μs protein-free membrane simulation, R is the gas constant.

## Supplementary Material

Supplement 1

Supplement 2

Supplement 3

Supplement 4

Supplement 5

Supplement 6

Supplement 7

## Figures and Tables

**Table 1.** Number of scrambling events in and out of the canonical groove pathway. Scrambling events where the lipid headgroup transitions between leaflets within 4.7 Å of the DOPC maximum density pathway. All other events were considered “out-of-the-groove”. For the full list of simulations and scrambling rates see Table S2.

homolog	PDB code	# of in-the-groove events	# of out-of-the-groove events	total	average scrambling rate (*μ*s^−1^)
nhTMEM16	4WIS	219	1	220	24.4±5.2
nhTMEM16	6QM6	141	0	141	15.7±3.9
afTMEM16	7RXG	96	0	96	10.7±2.9
TMEM16K	5OC9	66	8	74	8.2±2.9
TMEM16K	6R7X	0	4	4	0.4±0.7
TMEM16F F518H	8B8J	98	4	102	11.3±4.1
TMEM16F	6QP6*	24	3	27	3.0±1.6
TMEM16F T137Y	8TAG	0	9	9	1.0±0.7
TMEM16F	6P47	1	3	4	0.4±0.5
TMEM16F	6P48	0	4	4	0.4±0.5
TMEM16F F518H/Q623A	8BC0	0	4	4	0.4±0.5
TMEM16F F518H	8B8Q	0	4	4	0.4±0.7
TMEM16F F518H	8B8G	2	0	2	0.2±0.4
TMEM16F	6QPB	0	3	3	0.3±0.7
TMEM16A	7ZK3*^6^	0	11	11	1.2±1.6
TMEM16A	5OYB*	2	0	2	0.2±0.4
TMEM16A	7ZK3*^10^	0	1	1	0.1±0.3
TMEM16A	7ZK3*^8^	0	1	1	0.1±0.3

## SIGNIFICANCE STATEMENT

Mammalian members of the Ca^2+^-activated TMEM16 protein family of ion channels and lipid scramblases play roles in a range of biological phenomena such as blood coagulation and mucus secretion, and they have been implicated in several human cancers, neuronal disorder SCAR10, and SARS-CoV2 infection. Thus, a complete understanding of how each member facilitates ion and lipid movement would greatly augment our ability to treat these diseases. However, there is a major ongoing debate over whether an open protein-delineated pathway (“groove”) is necessary for scrambling, how much the TMEM16-induced membrane deformation contributes to scrambling, and whether all family members utilize the same mechanism for scrambling. Our coarse-grained molecular dynamics (MD) simulations of 27 TMEM16 structures from five family members settles each of these open questions. Consistently for all family members the most robust scrambling activity occurs when the groove is open, and the membrane is strongly thinned at the groove. We also observe scrambling events outside the groove, which are independent of the groove openness and membrane thinning; however, importantly, these events occur at much lower rates. Taken together, the molecular insights gained in this comprehensive computational survey improve our understanding of the biophysics of protein-mediated lipid scrambling and may aid the development of potential therapeutics that modulate TMEM16 activity.
