# Supplementary material for: Simulation-based survey of TMEM16 family reveals that robust lipid scrambling requires an open groove
Source: bioRxiv. 2024 Nov 13:2024.09.25.615027. Originally published 2024 Sep 27. Preprint. [Version 2] doi: 10.1101/2024.09.25.615027 (PMC11463437; doi:10.1101/2024.09.25.615027)
Supplement: Supplementary file 1 [file NIHPP2024.09.25.615027v2-supplement-1.pdf]

bioRxiv preprint doi: https://doi.org/10.1101/2024.09.25.615027; this version posted November 13, 2024. The copyright holder for this preprint (which was not certified by peer review) is the author/funder, who has granted bioRxiv a license to display the preprint in perpetuity. It is made available under aCC-BY 4.0 International license.

Figure Supplements

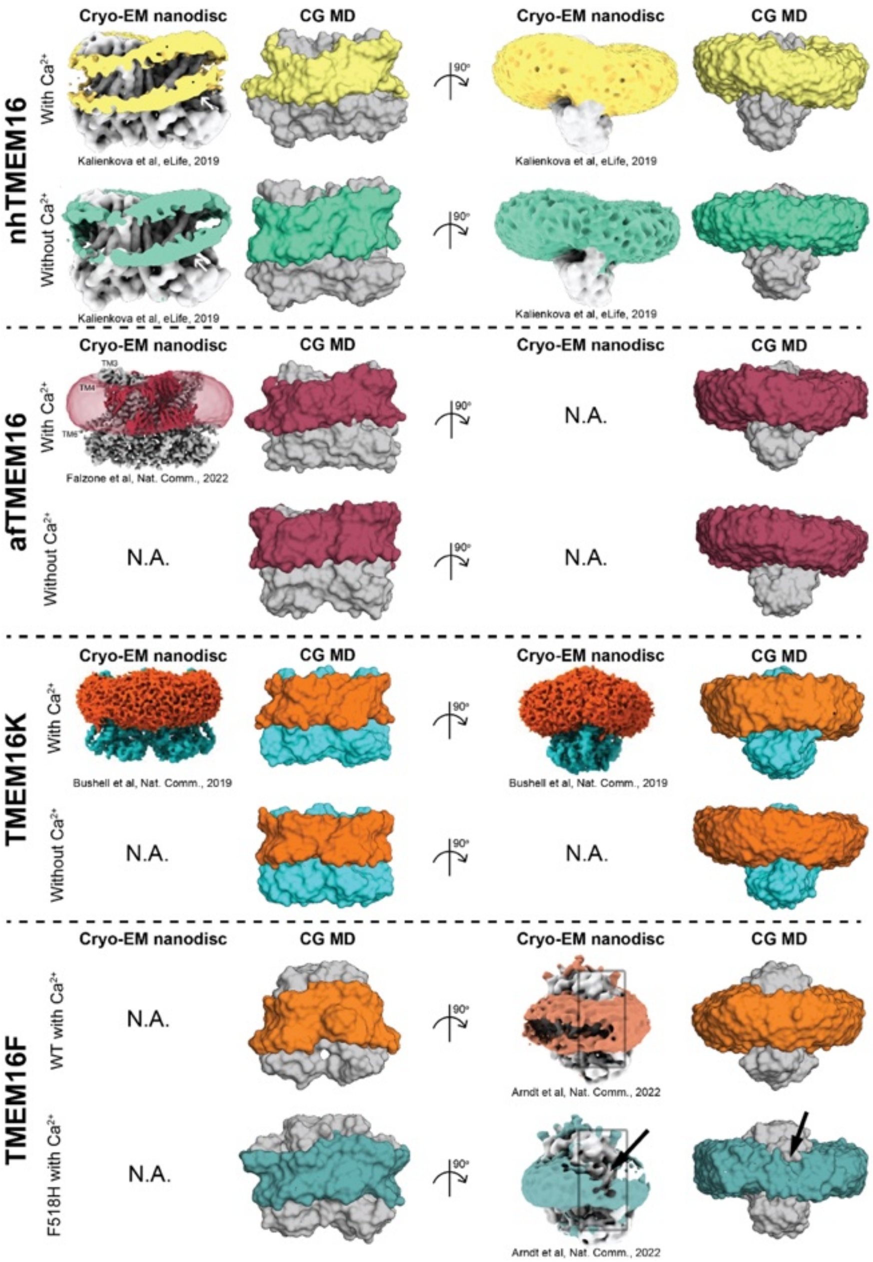

Figure 1-figure supplement 1. Comparison between membrane deformations in cryo-EM nanodiscs and CG MD. Front and side views of cryo-EM maps (left) and the final frames of our CG MD simulations (right). nhTMEM16: cryo-EM images (PDB IDs 6QM9 and 6QM4) were adapted from © 2019, Kalienkova *et al*, published by eLife (61). The CG MD structures are PDB IDs 4WIS (with Ca^{2+}) and 6QM4 (without Ca^{2+}). afTMEM16: cryo-EM image (PDB ID 7RXG) was adapted from © 2022, Falzone *et al*, published by Springer Nature (59). The CG MD structures are PDB IDs 7RXG (with Ca^{2+}) and 7RXB (without Ca^{2+}). TMEM16K: cryo-EM images (PDB ID 5OC9) were adapted from © 2019, Bushell *et al*, published by Springer Nature (12). The CG MD structures are PDB IDs 5OC9 (with Ca^{2+}) and 6R7X (without Ca^{2+}). TMEM16F: cryo-EM images (PDB IDs 6QPC and 8B8J) were adapted from © 2022, Arndt *et al*, published by Springer Nature (60). The CG MD structures are also PDB IDs 6QPC (WT with Ca^{2+}) and 8B8J (F518H with Ca^{2+}). Black arrows indicate a dip in lipid density near the groove entrance. All CG MD snapshots were rendered with PyMOL 2.5.0 (104), after selecting all lipid beads within 12 Å of the protein and matching the coloring to the colors used in the original cryo-EM images.

bioRxiv preprint doi: https://doi.org/10.1101/2024.09.25.615027; this version posted November 13, 2024. The copyright holder for this preprint (which was not certified by peer review) is the author/funder, who has granted bioRxiv a license to display the preprint in perpetuity. It is made available under aCC-BY 4.0 International license.

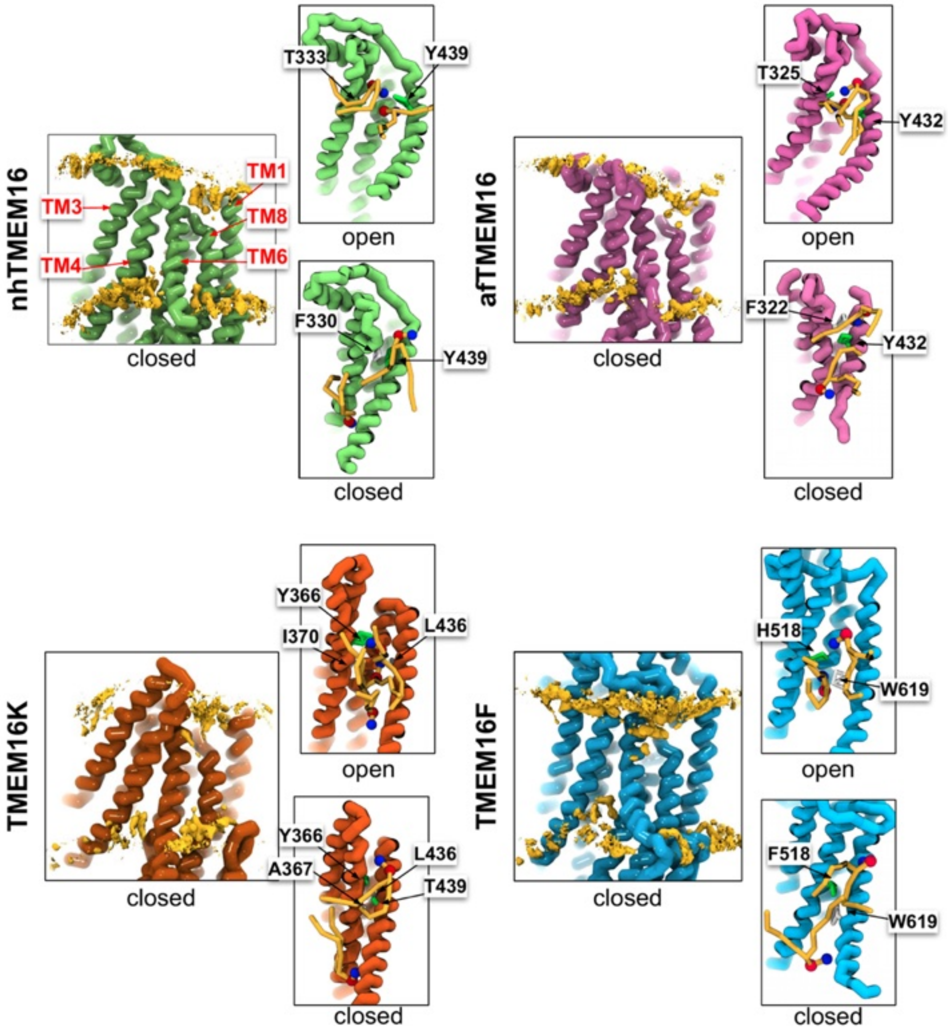

Figure 1-figure supplement 2. CG simulations of multiple TMEM16 structures with closed grooves lack lipid density in the TM4/TM6 pathway. Snapshots from CG MD simulations of nhTMEM16 (PDB IDs 6QM4 (closed) and 4WIS (open), green), afTMEM16 (PDB IDs 7RXB (closed) and 7RXG (open), violet), TMEM16K (PDB IDs 6R7X (closed) and 5OC9 (open), gold), and TMEM16F (PDB IDs 6QPB (closed) and 6QP6* (open), blue) with phosphatidylcholine (PC) lipid headgroup density (yellow) and nearby lipids (yellow). Residues forming the closest distance between TM4 and TM6 (colored by residue type: basic (red), acidic (blue), and polar (green)) and lipids near the groove also shown. Each density is averaged over both chains.

bioRxiv preprint doi: https://doi.org/10.1101/2024.09.25.615027; this version posted November 13, 2024. The copyright holder for this preprint (which was not certified by peer review) is the author/funder, who has granted bioRxiv a license to display the preprint in perpetuity. It is made available under aCC-BY 4.0 International license.

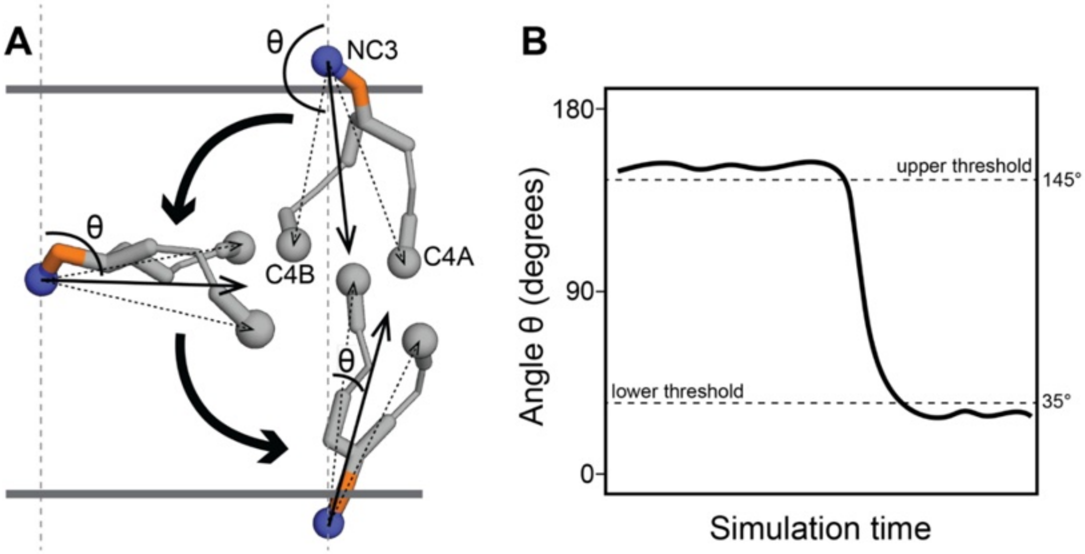

Figure 2-figure supplement 1. Measuring lipid angles to detect scrambling events. (A) For every time frame, for every lipid in the system, we defined a vector between the choline bead (NC3) and the two tail beads (C4A, C4B; dashed arrows) and calculated the angle θ between the average of those two vectors (solid arrow) with the z axis. **(B)** A schematic representation of a typical time trace for a lipid that scrambles from the upper membrane leaflet (θ ≈ 150°) to the lower membrane leaflet (θ ≈ 30°). A scrambling event is only counted when θ passes the threshold at the opposite leaflet with respect to its original location (35° for the lower, 145° for the upper).

bioRxiv preprint doi: https://doi.org/10.1101/2024.09.25.615027; this version posted November 13, 2024. The copyright holder for this preprint (which was not certified by peer review) is the author/funder, who has granted bioRxiv a license to display the preprint in perpetuity. It is made available under aCC-BY 4.0 International license.

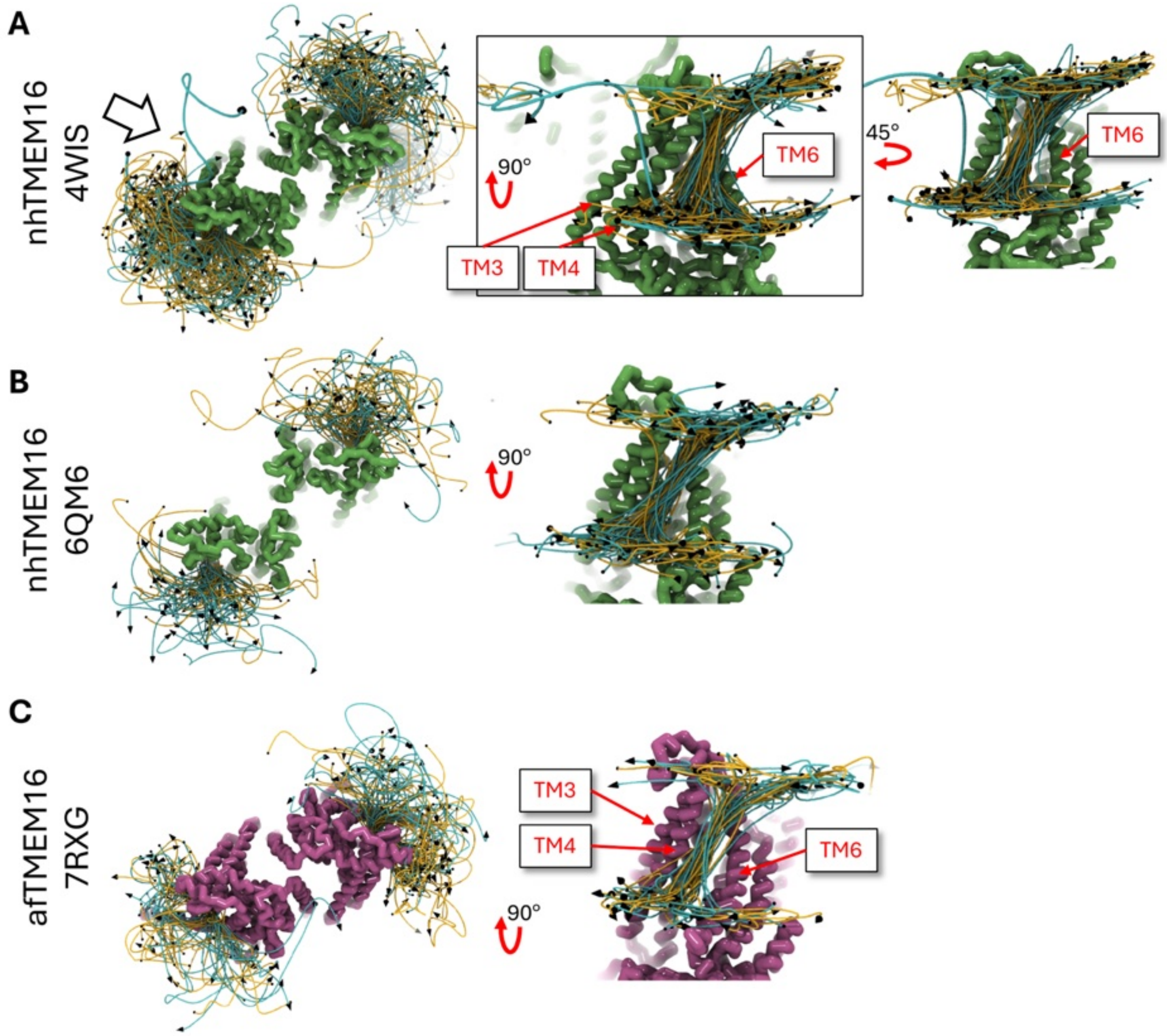

Figure 2-figure supplement 2. Position traces of scrambling lipids in fungal TMEM16 simulations. **(A)** Lipid traces for Ca^{2+}-bound open nhTMEM16 (PDB ID 4WIS). **(B)** Lipid traces for Ca^{2+}-bound open nhTMEM16 (PDB ID 6QM6). **(C)** Lipid traces for Ca^{2+} -bound open afTMEM16 (PDB ID 7RXG). Lipid traces are generated by fitting raw lipid headgroup center of mass positions to a smooth spline curve.

bioRxiv preprint doi: https://doi.org/10.1101/2024.09.25.615027; this version posted November 13, 2024. The copyright holder for this preprint (which was not certified by peer review) is the author/funder, who has granted bioRxiv a license to display the preprint in perpetuity. It is made available under aCC-BY 4.0 International license.

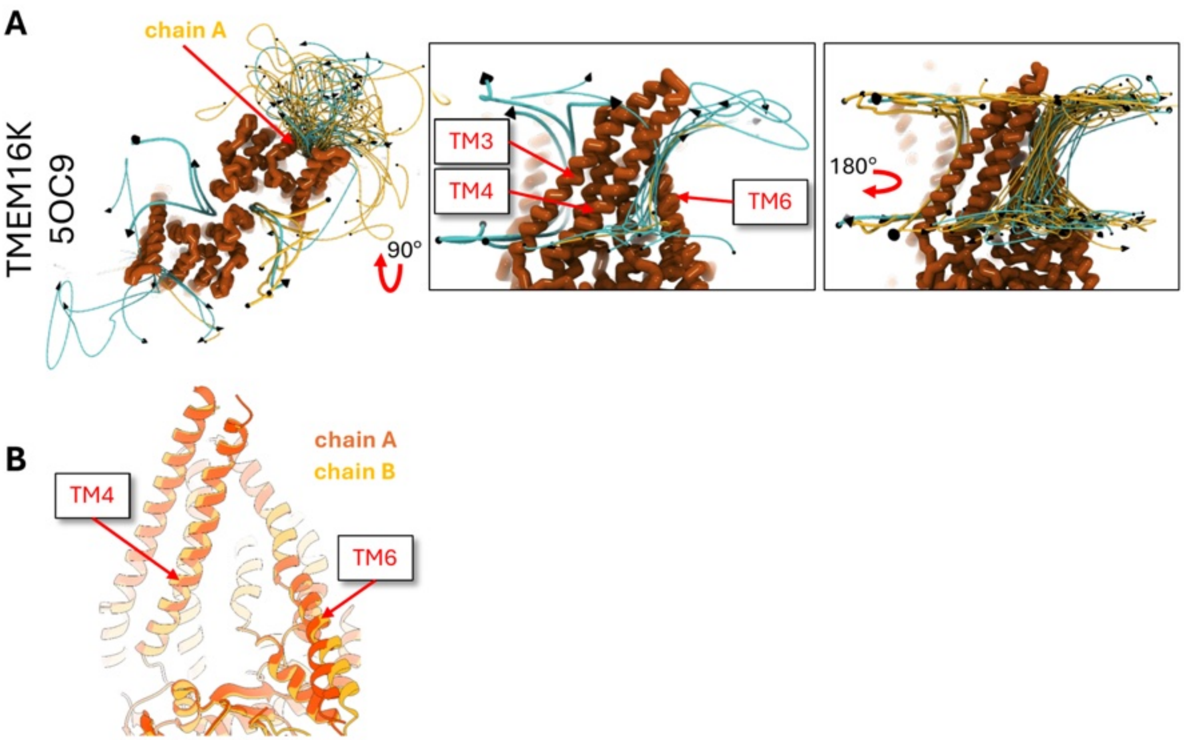

Figure 2-figure supplement 3. Position traces of scrambling lipids in the open TMEM16K simulation. (A) Lipid traces for Ca^{2+}-bound open TMEM16K (PDB ID 5OC9). **(B)** Cartoon representation of aligned subunits of Ca^{2+}-bound TMEM16K (PDB ID 5OC9). Lipid traces are generated by fitting raw lipid headgroup center of mass positions to a smooth spline curve.

bioRxiv preprint doi: https://doi.org/10.1101/2024.09.25.615027; this version posted November 13, 2024. The copyright holder for this preprint (which was not certified by peer review) is the author/funder, who has granted bioRxiv a license to display the preprint in perpetuity. It is made available under aCC-BY 4.0 International license.

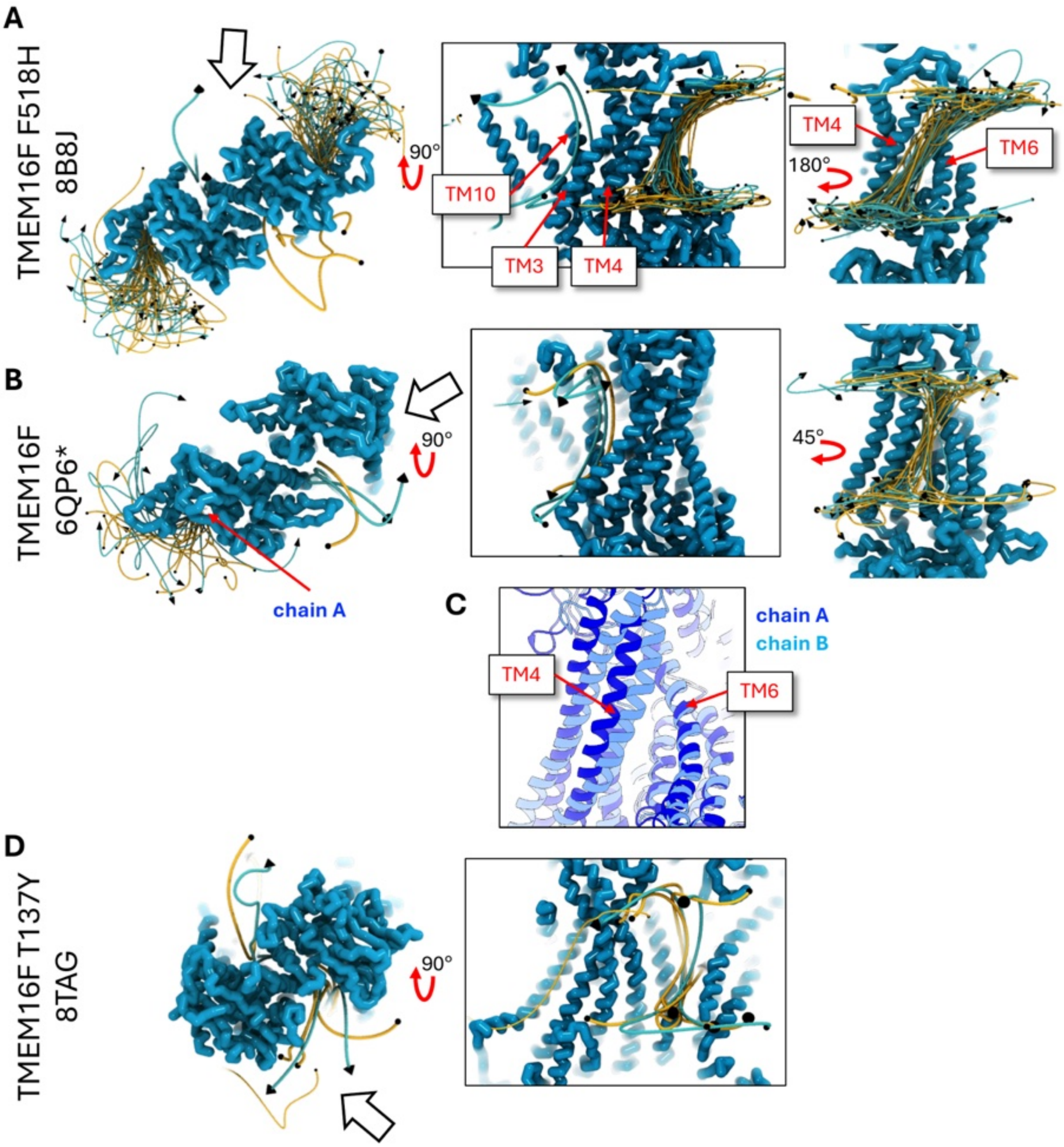

Figure 2-figure supplement 4. Position traces of scrambling lipids in TMEM16F simulations. (A) Lipid traces for Ca^{2+} -bound open TMEM16F F518H mutant (PDB ID 8B8J). **(B)** Lipid traces for Ca^{2+} -bound simulated open TMEM16F (6QP6*) **(C)** Cartoon representation of aligned subunits of Ca^{2+}-bound simulated open TMEM16F (6QP6*). **(D)** Lipid traces for Ca^{2+} -bound simulated closed TMEM16F (PDB ID 8TAG). Lipid traces are generated by fitting raw lipid headgroup center of mass positions to a smooth spline curve.

bioRxiv preprint doi: https://doi.org/10.1101/2024.09.25.615027; this version posted November 13, 2024. The copyright holder for this preprint (which was not certified by peer review) is the author/funder, who has granted bioRxiv a license to display the preprint in perpetuity. It is made available under aCC-BY 4.0 International license.

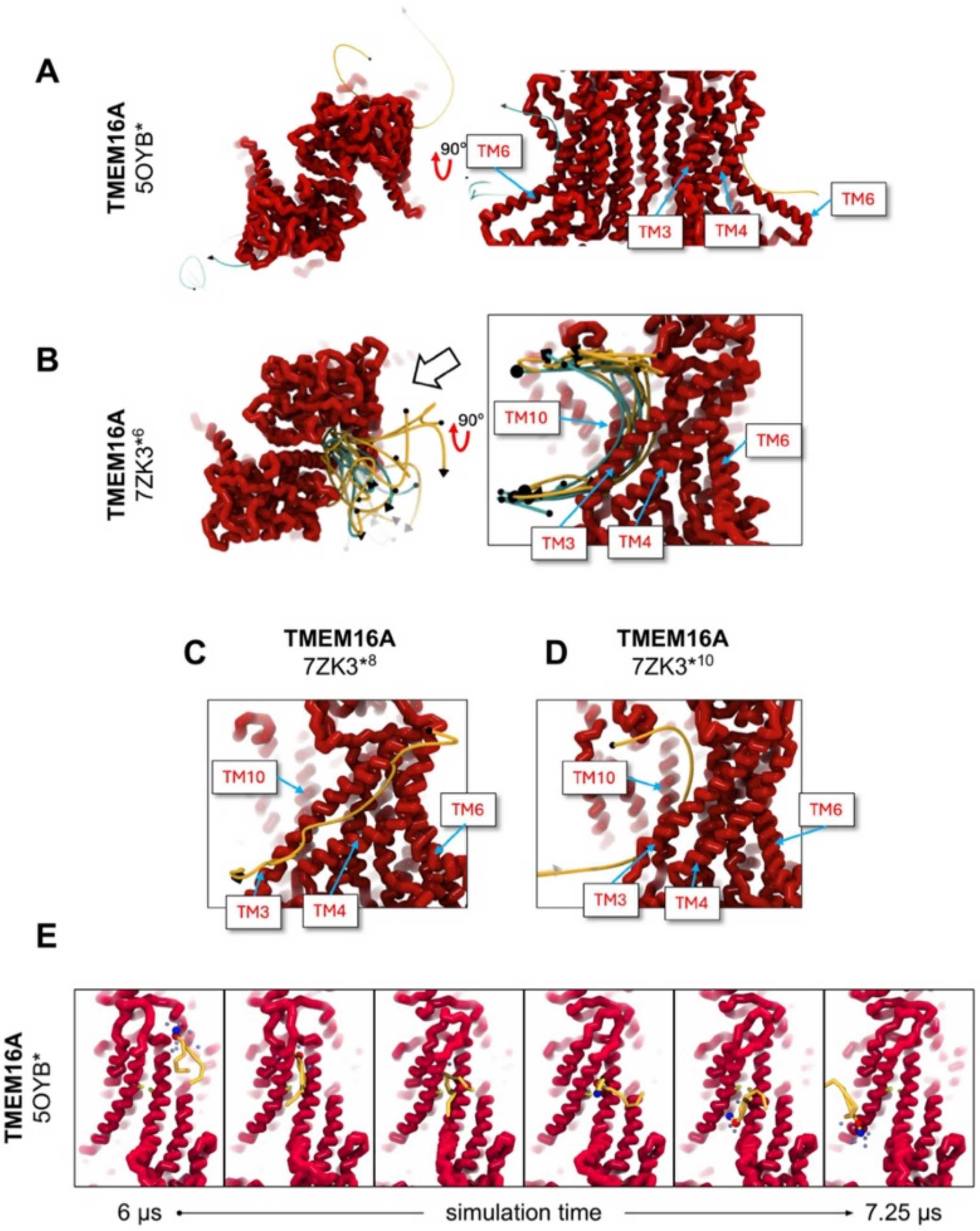

Figure 2-figure supplement 5. Position traces of scrambling lipids in TMEM16A simulations. (A) Lipid traces for Ca^{2+} -bound simulated conductive TMEM16A (5OYB*). **(B)** Lipid traces for Ca^{2+} -bound simulated open TMEM16A (7ZK3*^{6}). **(C)** Lipid traces for Ca^{2+} -bound simulated open TMEM16A (7ZK3*^{10}). **(D)** Lipid traces for Ca^{2+} -bound simulated open TMEM16A (7ZK3*^{8}). **(E)** Snapshots from simulation of 5OYB* during lipid scrambling event (trace in **A**). Lipid traces are generated by fitting raw lipid headgroup center of mass positions to a smooth spline curve.

bioRxiv preprint doi: https://doi.org/10.1101/2024.09.25.615027; this version posted November 13, 2024. The copyright holder for this preprint (which was not certified by peer review) is the author/funder, who has granted bioRxiv a license to display the preprint in perpetuity. It is made available under aCC-BY 4.0 International license.

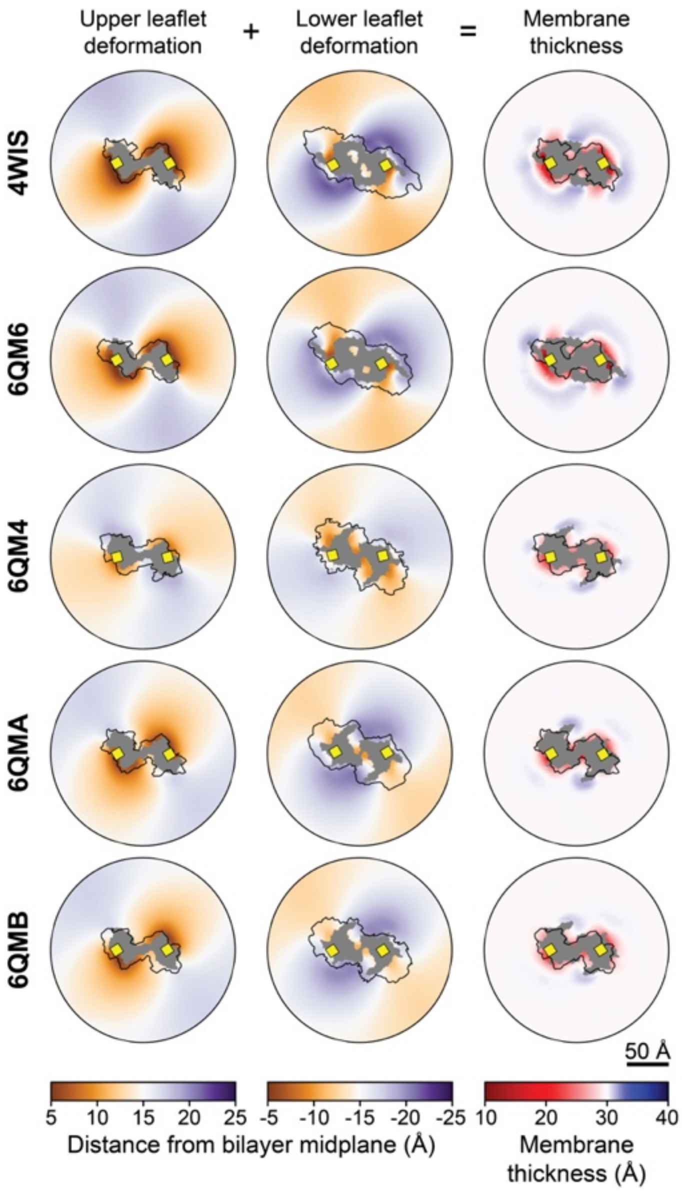

Figure 3-figure supplement 1. Membrane deformations for simulated nhTMEM16 structures. Left column: xy-map of the distance along the z-axis from the bilayer midplane to the ensemble averaged positions of the glycerol linker (GL1 and GL2 beads). Middle column: xy-map of the distance along the z-axis from the bilayer midplane to the ensemble averaged positions of the glycerol linker (GL1 and GL2 beads). Right column: the sum of the upper and lower leaflet deformations, representing the bilayer thickness along z. In all plots, grey areas indicate grid points with lipid occupancy <2%. The black outline is the projected surface of the upper (z>0) or lower (z<0) portion of the protein dimer.

bioRxiv preprint doi: https://doi.org/10.1101/2024.09.25.615027; this version posted November 13, 2024. The copyright holder for this preprint (which was not certified by peer review) is the author/funder, who has granted bioRxiv a license to display the preprint in perpetuity. It is made available under aCC-BY 4.0 International license.

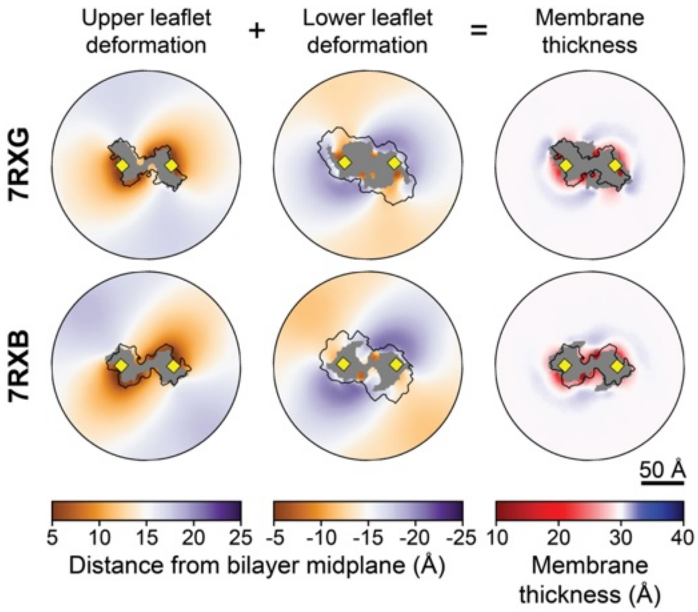

Figure 3-figure supplement 2. Membrane deformations for simulated afTMEM16 structures. Left column: xy-map of the distance along the z-axis from the bilayer midplane to the ensemble averaged positions of the glycerol linker (GL1 and GL2 beads). Middle column: xy-map of the distance along the z-axis from the bilayer midplane to the ensemble averaged positions of the glycerol linker (GL1 and GL2 beads). Right column: the sum of the upper and lower leaflet deformations, representing the bilayer thickness along z. In all plots, grey areas indicate grid points with lipid occupancy <2%. The black outline is the projected surface of the upper (z>0) or lower (z<0) portion of the protein dimer.

bioRxiv preprint doi: https://doi.org/10.1101/2024.09.25.615027; this version posted November 13, 2024. The copyright holder for this preprint (which was not certified by peer review) is the author/funder, who has granted bioRxiv a license to display the preprint in perpetuity. It is made available under aCC-BY 4.0 International license.

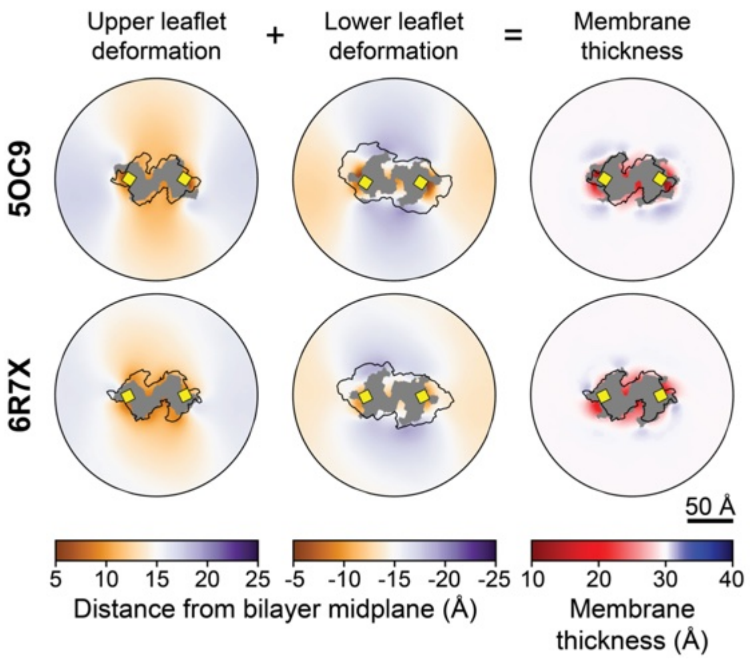

Figure 3-figure supplement 3. Membrane deformations for simulated TMEM16K structures. Left column: xy-map of the distance along the z-axis from the bilayer midplane to the ensemble averaged positions of the glycerol linker (GL1 and GL2 beads). Middle column: xy-map of the distance along the z-axis from the bilayer midplane to the ensemble averaged positions of the glycerol linker (GL1 and GL2 beads). Right column: the sum of the upper and lower leaflet deformations, representing the bilayer thickness along z. In all plots, grey areas indicate grid points with lipid occupancy <2%. The black outline is the projected surface of the upper (z>0) or lower (z<0) portion of the protein dimer.

bioRxiv preprint doi: https://doi.org/10.1101/2024.09.25.615027; this version posted November 13, 2024. The copyright holder for this preprint (which was not certified by peer review) is the author/funder, who has granted bioRxiv a license to display the preprint in perpetuity. It is made available under aCC-BY 4.0 International license.

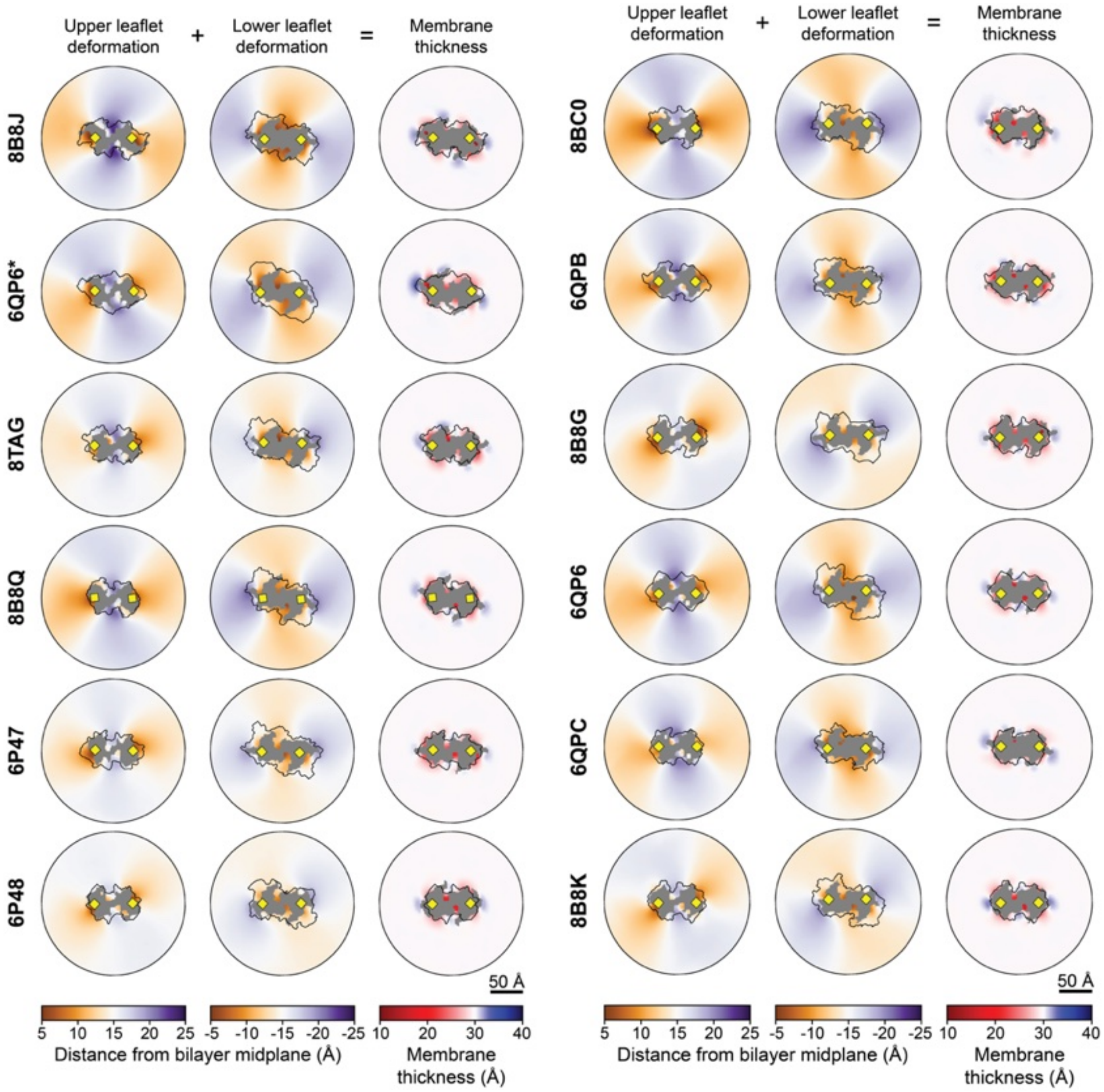

Figure 3-figure supplement 4. Membrane deformations for simulated TMEM16F structures. Left column: xy-map of the distance along the z-axis from the bilayer midplane to the ensemble averaged positions of the glycerol linker (GL1 and GL2 beads). Middle column: xy-map of the distance along the z-axis from the bilayer midplane to the ensemble averaged positions of the glycerol linker (GL1 and GL2 beads). Right column: the sum of the upper and lower leaflet deformations, representing the bilayer thickness along z. In all plots, grey areas indicate grid points with lipid occupancy <2%. The black outline is the projected surface of the upper (z>0) or lower (z<0) portion of the protein dimer.

bioRxiv preprint doi: https://doi.org/10.1101/2024.09.25.615027; this version posted November 13, 2024. The copyright holder for this preprint (which was not certified by peer review) is the author/funder, who has granted bioRxiv a license to display the preprint in perpetuity. It is made available under aCC-BY 4.0 International license.

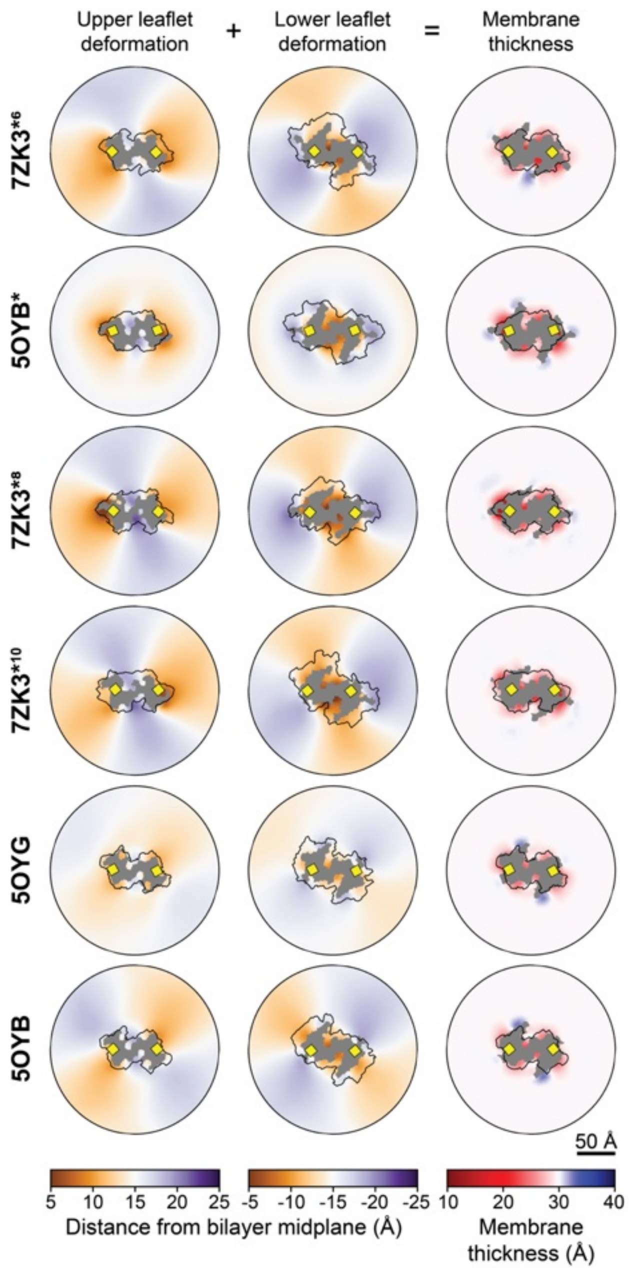

Figure 3-figure supplement 5. Membrane deformations for simulated TMEM16A structures. Left column: xy-map of the distance along the z-axis from the bilayer midplane to the ensemble averaged positions of the glycerol linker (GL1 and GL2 beads). Middle column: xy-map of the distance along the z-axis from the bilayer midplane to the ensemble averaged positions of the glycerol linker (GL1 and GL2 beads). Right column: the sum of the upper and lower leaflet deformations, representing the bilayer thickness along z. In all plots, grey areas indicate grid points with lipid occupancy <2%. The black outline is the projected surface of the upper (z>0) or lower (z<0) portion of the protein dimer.

bioRxiv preprint doi: https://doi.org/10.1101/2024.09.25.615027; this version posted November 13, 2024. The copyright holder for this preprint (which was not certified by peer review) is the author/funder, who has granted bioRxiv a license to display the preprint in perpetuity. It is made available under aCC-BY 4.0 International license.

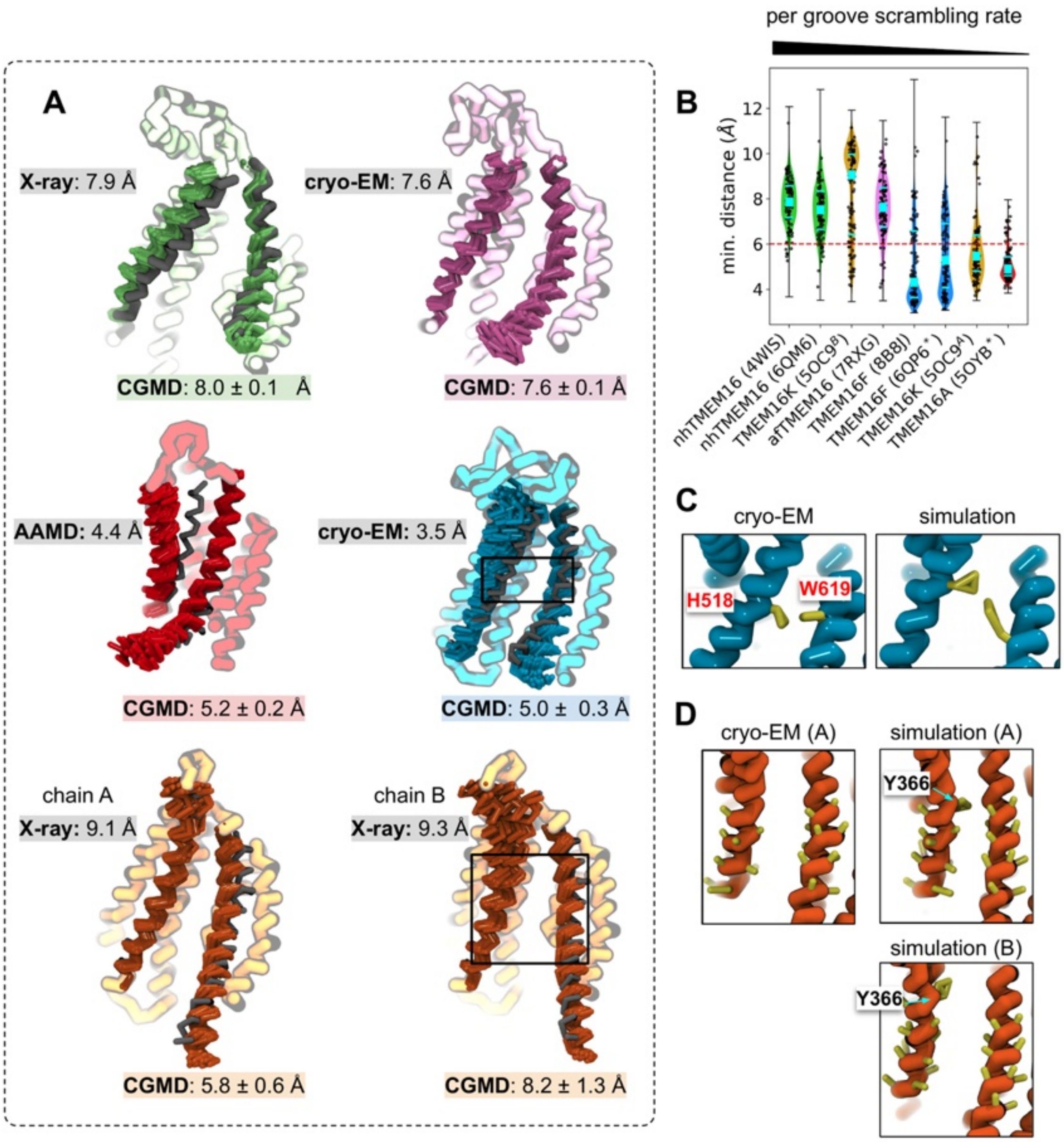

Figure 3-figure supplement 6. TM4 moves away from starting structure coordinates in open states. **(A)** Overlayed CG representations of experimentally determined or simulated starting structures (grey) and snapshots from CG simulations. nhTMEM16 4WIS (green), afTMEM16 7RXG (violet), TMEM16K 5OC9 (orange), TMEM16F 8B8J (blue), and TMEM16A 5OYB* (red) with minimum TM4-6 distances for the starting CG structure (grey) and mean values with standard deviation from simulations. **(B)** Violin plots of minimum distances between TM4 and TM6 in a single groove with median value (cyan square) and 25-75% quartiles (cyan bars). **(C)** TMEM16F TM4/TM6 groove constriction point at residues F518H (on TM4) and W619 (on TM6). **(D)** TMEM16K TM4/TM6 groove constriction point at Y366 (on TM4).

bioRxiv preprint doi: https://doi.org/10.1101/2024.09.25.615027; this version posted November 13, 2024. The copyright holder for this preprint (which was not certified by peer review) is the author/funder, who has granted bioRxiv a license to display the preprint in perpetuity. It is made available under aCC-BY 4.0 International license.

Figure 3-video 1. Lipid scrambling by open Ca^{2+}-bound nhTMEM16 (PDB ID 4WIS). Stick representation of TM3-8 (green, rest of protein not shown) and lipid headgroups (red and blue spheres) within 7 Å of the protein. Video shows ~600 ns of the last 1 μs of simulation with positional averaging over 3 frames. Residues T333, L336 and Y439 shown as spheres colored by residue type: hydrophobic (white) and polar (green).

Figure 3-video 2. Lipid scrambling by open Ca^{2+}-bound TMEM16K chain B (PDB ID 5OC9). Stick representation of TM3-8 (orange, rest of protein not shown) and lipid headgroups (red and blue spheres) within 7 Å of the protein. Video shows ~600 ns of the last 1 μs of simulation with positional averaging over 3 frames. Residues Y366, I370, T435, L436 and T439 shown as spheres colored by residue type: hydrophobic (white) and polar (green).

Figure 3-video 3. Lipid scrambling by open Ca^{2+}-bound TMEM16F F518H (PDB ID 8B8J). Stick representation of TM3-8 (cyan, rest of protein not shown) and lipid headgroups (red and blue spheres) within 7 Å of the protein. Video shows ~600 ns of the last 1 μs of simulation with positional averaging over 3 frames. Residues H518, M522 and W619 shown as spheres colored by residue type: hydrophobic (white), and polar (green).

Figure 3-video 4. Lipid scrambling event 1/2 by simulated ion conductive Ca^{2+}-bound TMEM16A (5OYB*). Stick representation of TM3-8 (pink, rest of protein not shown) and lipid headgroups (red and blue beads) within 7 Å of the protein. Scrambling lipid tail colored yellow. Residues I550, I551, K645, Q649 and E633 shown as spheres colored by residue type: acidic (red), basic (blue), polar (green) and hydrophobic (white). Video shows 3900-4780 ns of simulation with positional averaging over 3 frames.

Figure 3-video 5. Lipid scrambling by simulated open Ca^{2+}-bound TMEM16F (PDB ID 6QP6*). Stick representation of TM3-8 (cyan, rest of protein not shown) and lipid headgroups (red and blue spheres) within 7 Å of the protein. Video shows ~600 ns of the last 1 μs of simulation with positional averaging over 3 frames. Residues F518, M522 and W619 shown as spheres colored by residue type: hydrophobic (white).

Figure 3-video 6. Lipid scrambling by open Ca^{2+}-bound TMEM16K chain A (PDB ID 5OC9). Stick representation of TM3-8 (orange, rest of protein not shown) and lipid headgroups (red and blue spheres) within 7 Å of the protein. Video shows ~600 ns of the last 1 μs of simulation with positional averaging over 3 frames. Residues Y366, I370, T435, L436 and T439 shown as spheres colored by residue type: hydrophobic (white) and polar (green).

bioRxiv preprint doi: https://doi.org/10.1101/2024.09.25.615027; this version posted November 13, 2024. The copyright holder for this preprint (which was not certified by peer review) is the author/funder, who has granted bioRxiv a license to display the preprint in perpetuity. It is made available under aCC-BY 4.0 International license.

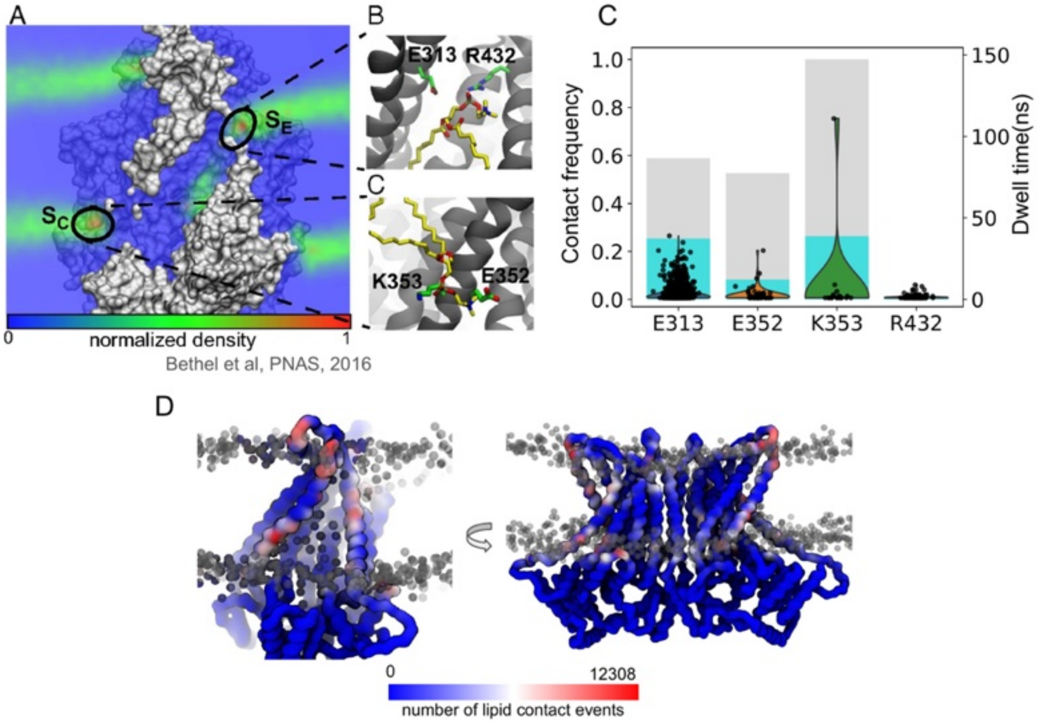

Figure 4-figure supplement 1. Contact analysis of lipid headgroup high density sites identified from previous AA simulation. (A-C) Charged residues and nearby POPC lipids near two high lipid phosphate density sites at the intracellular and extracellular entry of the open nhTMEM16 (PDB ID 4WIS) canonical groove ("S_C", "S_E") identified from previous AA simulation © 2016, Bethel & Grabe, published by PNAS (36). **(C)** Contact frequency with any lipid (grey bars) and only scrambling lipids (cyan bars). Dwell times with scrambling lipids shown as black points. **(D)** The nhTMEM16 CG backbone colored by total number of contact events with any lipid. Grey spheres indicate lipid headgroup positions in a single snapshot.

bioRxiv preprint doi: https://doi.org/10.1101/2024.09.25.615027; this version posted November 13, 2024. The copyright holder for this preprint (which was not certified by peer review) is the author/funder, who has granted bioRxiv a license to display the preprint in perpetuity. It is made available under aCC-BY 4.0 International license.

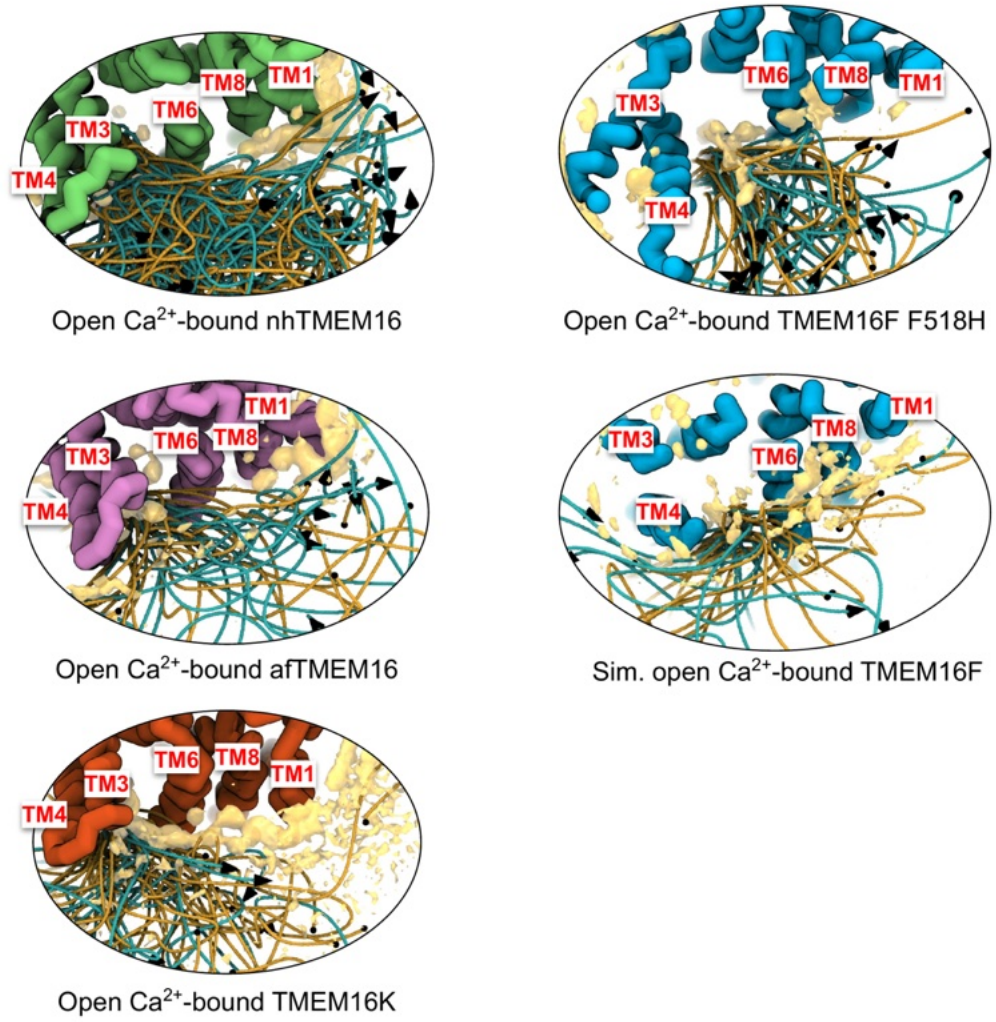

Figure 4-figure supplement 2. Position traces of scrambling lipids with total lipid headgroup density. Lipid traces and lipid headgroup density (yellow) for Ca^{2+} -bound scrambling competent TMEM16s: nhTMEM16 (PDB ID 4WIS, green), afTMEM16 (PDB ID 7RXG, purple), TMEM16K (PDB ID 5OC9, orange), TMEM16F F518H (PDB ID 8B8J, blue) and simulated TMEM16F (6QP6*). Each image is viewed from the extracellular or cytosolic (TMEM16K) space. Lipid traces are generated by fitting raw lipid headgroup center of mass positions to a smooth spline curve.

bioRxiv preprint doi: https://doi.org/10.1101/2024.09.25.615027; this version posted November 13, 2024. The copyright holder for this preprint (which was not certified by peer review) is the author/funder, who has granted bioRxiv a license to display the preprint in perpetuity. It is made available under aCC-BY 4.0 International license.

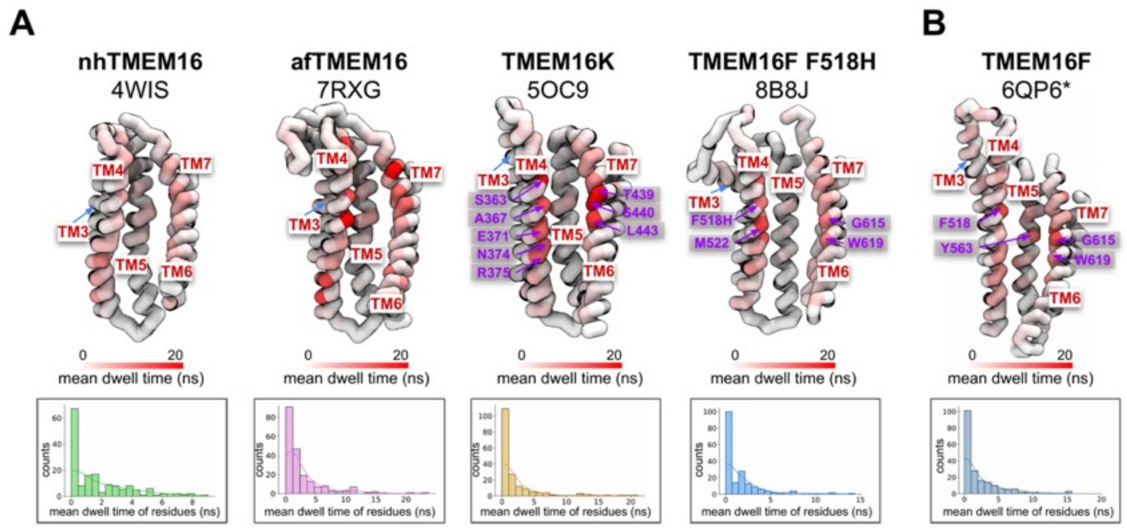

Figure 4-figure supplement 3. Average duration of interaction between scrambling lipids and TM4/TM6 groove lining residues. The canonical groove of each experimentally solved **(A)** and simulated **(B)** open scramblase structure is colored by the average duration of each interaction (dwell time) between scrambling lipids and groove lining residues. The distribution of average dwell times at individual residues is shown as a histogram below each structure.

bioRxiv preprint doi: https://doi.org/10.1101/2024.09.25.615027; this version posted November 13, 2024. The copyright holder for this preprint (which was not certified by peer review) is the author/funder, who has granted bioRxiv a license to display the preprint in perpetuity. It is made available under aCC-BY 4.0 International license.

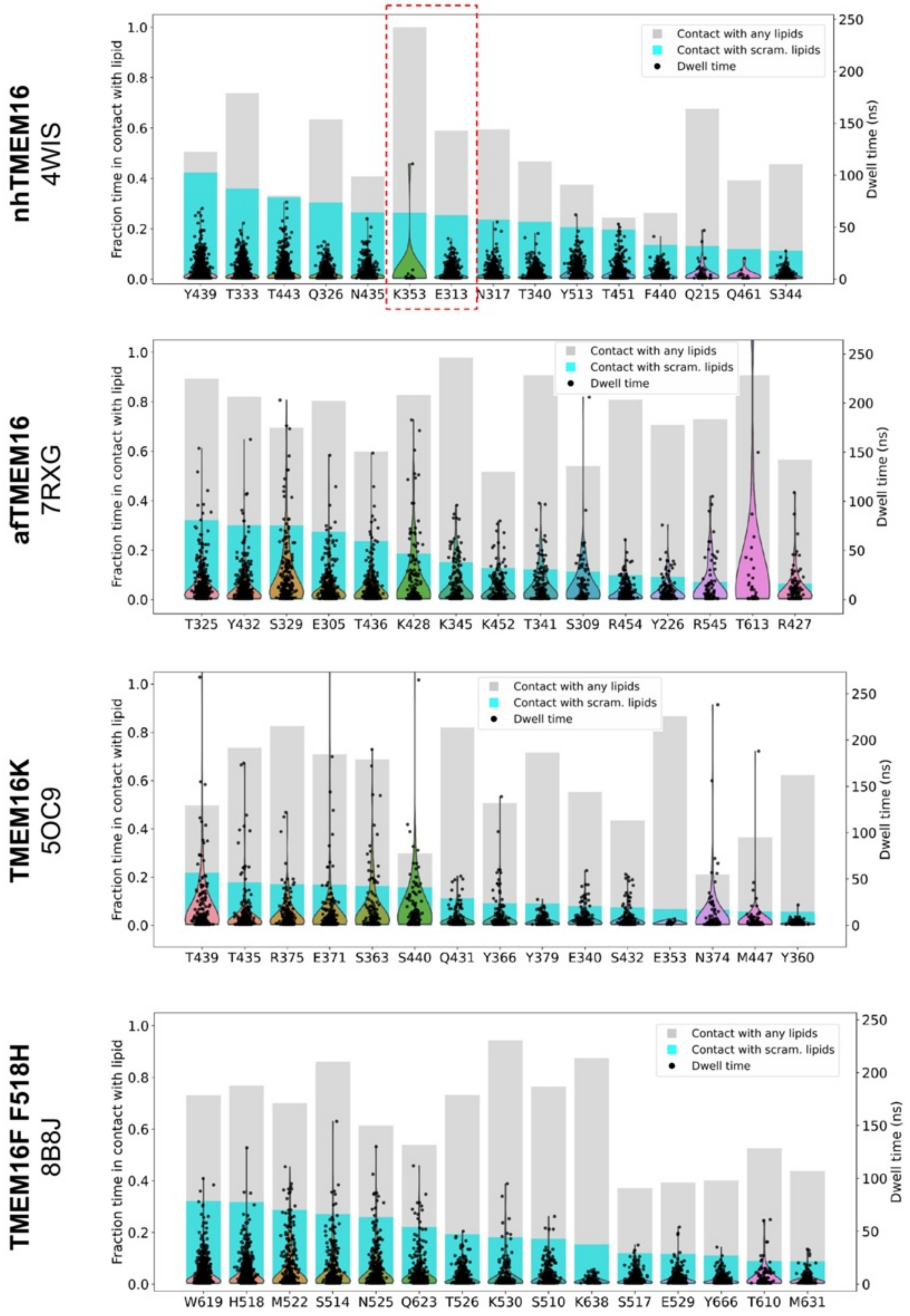

Figure 4-figure supplement 4. Dwell time distribution and contact frequency for TM4/TM6 groove lining residue across homologs. Contact frequency (cyan bar, left y-axis) and distribution of interaction dwell times (black scatter dots, right y-axis) between scrambling lipids and canonical groove lining

bioRxiv preprint doi: https://doi.org/10.1101/2024.09.25.615027; this version posted November 13, 2024. The copyright holder for this preprint (which was not certified by peer review) is the author/funder, who has granted bioRxiv a license to display the preprint in perpetuity. It is made available under aCC-BY 4.0 International license.

residues with the 15 longest average interaction dwell times. Residues are sorted by the contact frequency. Frequency of contact with any lipid (scrambling and non-scrambling lipids taken together) is shown as grey bar. The red dashed-line rectangle indicates two previously identified residues near high lipid phosphate density in an all-atom (AA) simulation (36).

bioRxiv preprint doi: https://doi.org/10.1101/2024.09.25.615027; this version posted November 13, 2024. The copyright holder for this preprint (which was not certified by peer review) is the author/funder, who has granted bioRxiv a license to display the preprint in perpetuity. It is made available under aCC-BY 4.0 International license.

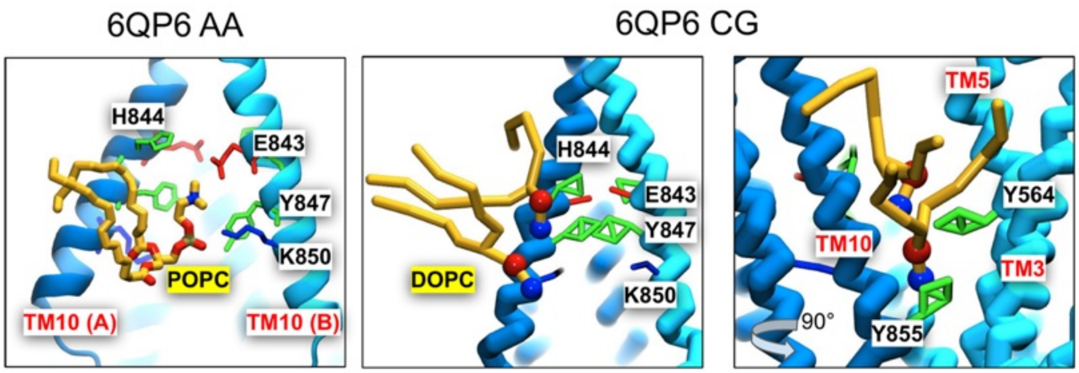

Figure 4-figure supplement 5. Lipids enter the dimer interface in atomistic and CG simulations of TMEM16F. Left: a snapshot of a 1-palmitoyl-2-oleoyl-glycero-3-phosphocholine (POPC) lipid that entered the TMEM16F (PDB ID 6QP6) dimer interface from the outer leaflet during an all-atom (AA) simulation. Right: snapshots at the same timepoint of 1,2-dioleoyl-sn-glycero-3-phosphocholine (DOPC) lipids that entered the TMEM16F (PDB ID 6QM6) dimer interface during a CG simulation. Nearby side chains are colored by residue type: basic (red), acidic (blue), and polar (green).

bioRxiv preprint doi: https://doi.org/10.1101/2024.09.25.615027; this version posted November 13, 2024. The copyright holder for this preprint (which was not certified by peer review) is the author/funder, who has granted bioRxiv a license to display the preprint in perpetuity. It is made available under aCC-BY 4.0 International license.

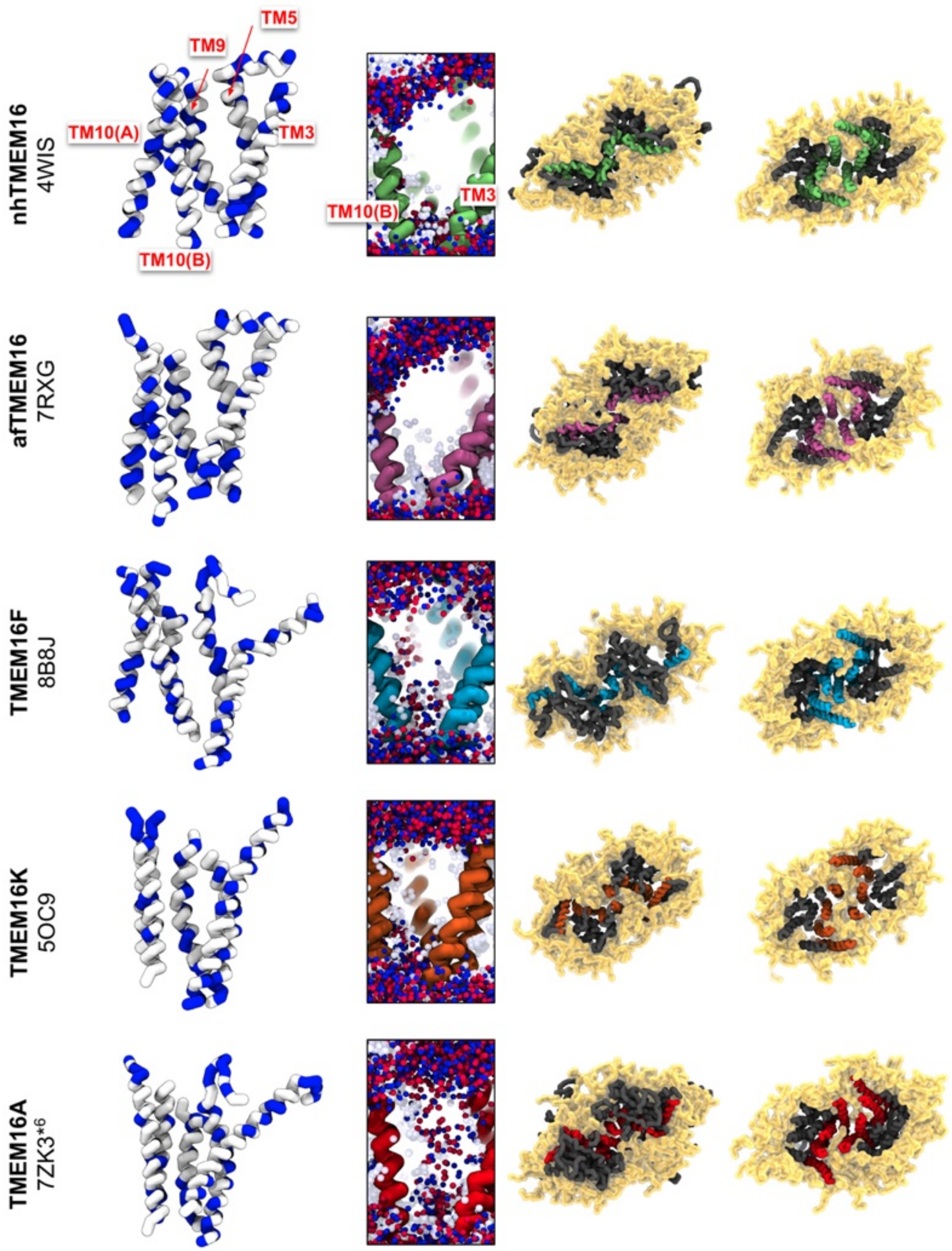

Figure 4-figure supplement 6. Dimer interface hydrophobicity and lipid positions. Images in each row were taken from CG MD simulations of 5 different TMEM16s. The first column depicts TM helices forming one half of the dimer interface (rest of the protein not shown) colored by residue type (small/hydrophobic: white, charge/polar: blue). The second column depicts snapshots of the same dimer interface helices with overlayed positions of water (light blue spheres) and lipid head groups (red and blue spheres) every 100 frames of the last 9 μs of each simulation. The last two columns are two views of the same snapshot showing the protein with its annulus of lipids (yellow). The dimer interface-forming helices are colored green (nhTMEM16), purple (afTMEM16), blue (TMEM16F), orange (TMEM16K), and red (TMEM16A).

bioRxiv preprint doi: https://doi.org/10.1101/2024.09.25.615027; this version posted November 13, 2024. The copyright holder for this preprint (which was not certified by peer review) is the author/funder, who has granted bioRxiv a license to display the preprint in perpetuity. It is made available under aCC-BY 4.0 International license.

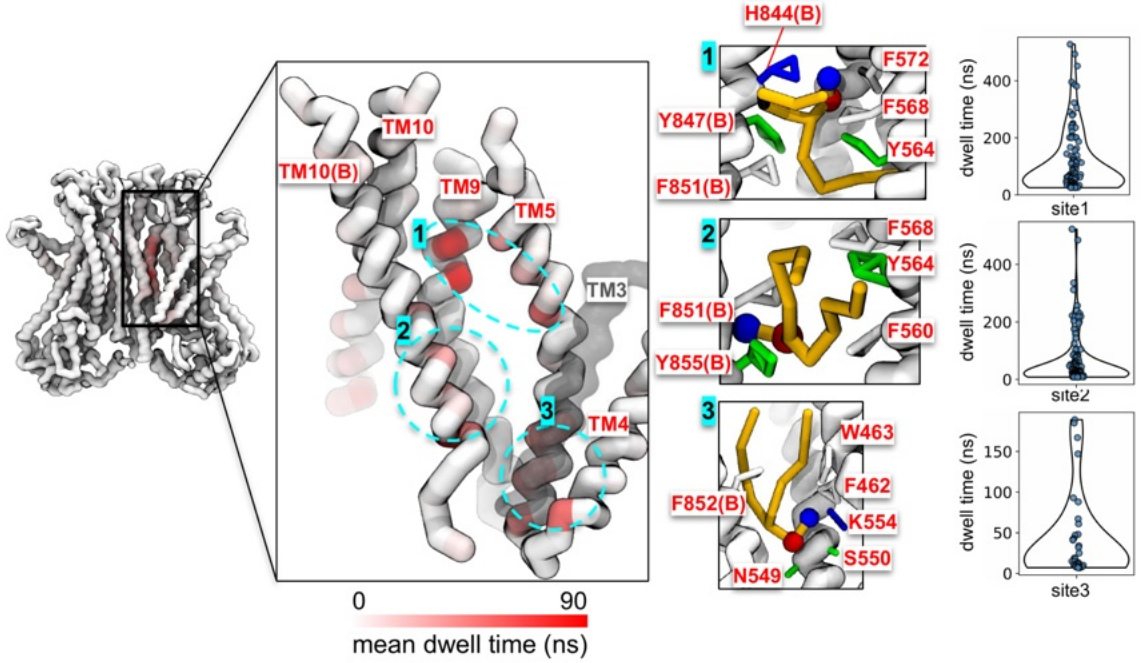

Figure 4-figure supplement 7. Dwell time analysis for scrambling events observed at TMEM16F F518H mutant dimer interface. The scrambling region is delineated by TM3, TM4, TM5, TM9, and TM10 both monomers. TM3 and part of TM5 are transparent for clarity. The backbone region of the dimer interface is colored by dwell time of the scrambling lipids. Sites with prolonged dwell time are circled in cyan (center left) and shown in zoomed-in images (center right). Distributions of dwell times at each site are shown as violin plots (far right).
