# Supplementary material for: Robust lipid scrambling by TMEM16 proteins requires an open groove and thin membrane
Source: bioRxiv. 2024 Sep 27:2024.09.25.615027. Preprint. [Version 1] doi: 10.1101/2024.09.25.615027 (PMC11463437; doi:10.1101/2024.09.25.615027)
Supplement: Supplementary file 1 [file media-1.pdf]

SUPPLEMENTAL INFORMATION

Robust lipid scrambling by TMEM16 proteins requires an open groove and thin membrane

Christina A. Stephens[1,2,3*], Niek van Hilten[1,3*], Lisa Zheng[1,2,3*], Michael Grabe[1,3,#]

[1]Cardiovascular Research Institute
[2]Graduate Group in Biophysics
[3]Department of Pharmaceutical Chemistry
University of California, San Francisco, CA 94158

[*] These authors contributed equally
[#]Corresponding author (michael.grabe@ucsf.edu)

Supplemental Video S1. Lipid scrambling event 1/2 by simulated ion conductive Ca^{2+}-bound TMEM16A (5OYB*). Cartoon representation of TM3-8 (pink) and lipid headgroups (red and blue beads) within 7 Å of the protein. Scrambling lipid tail colored yellow. Residues I550 and K645 shown as spheres colored by colored by residue type: acidic (blue) and hydrophobic (white). Video shows 3900-4780 ns of simulation with positional averaging over 3 frames.

Supplemental Video S2. Lipid scrambling by open Ca^{2+}-bound nhTMEM16 (PDB ID 4WIS). Cartoon representation of TM3-8 (green) and lipid headgroups (red and blue beads) within 7 Å of the protein. Video shows last 1000 ns of simulation with positional averaging over 3 frames. Residues L336, T333 and Y439 shown as spheres colored by colored by residue type: hydrophobic (white) and polar (green).

Supplemental Video S3. Lipid scrambling by open Ca^{2+}-bound TMEM16F F518H (PDB ID 8B8J). Cartoon representation of TM3-8 (cyan) and lipid headgroups (red and blue beads) within 7 Å of the protein. Video shows last 1000 ns of simulation with positional averaging over 3 frames. Residues H518 and W619 shown as spheres colored by colored by residue type: hydrophobic (white), and polar (green).

Supplemental Video S4. Lipid scrambling by simulated open Ca^{2+}-bound TMEM16F (PDB ID 6QP6*). Cartoon representation of TM3-8 (cyan) and lipid headgroups (red and blue beads) within 7 Å of the protein. Video shows last 1000 ns of simulation with positional averaging over 3 frames. Residues F518 and W619 shown as spheres colored by colored by residue type: hydrophobic (white).

Supplemental Video S5. Lipid scrambling by open Ca^{2+}-bound TMEM16K chain B (PDB ID 5OC9). Cartoon representation of TM3-8 (orange) and lipid headgroups (red and blue beads) within 7 Å of the protein. Video shows last 1000 ns of simulation with positional averaging over 3 frames. Residues I370, Y366 and L436 shown as spheres colored by colored by residue type: hydrophobic (white) and polar (green).

Supplemental Video S6. Lipid scrambling by open Ca^{2+}-bound TMEM16K chain A (PDB ID 5OC9). Cartoon representation of TM3-8 (orange) and lipid headgroups (red and blue beads) within 7 Å of the protein. Video shows last 1000 ns of simulation with positional averaging over 3 frames. Residues I370, Y366 and L436 shown as spheres colored by colored by residue type: hydrophobic (white) and polar (green).

homolog	PDB ID	structure method	organism	modeled loops
**nhTMEM16**	4WIS	xray	fusarium vanettenii 77-13-4	128-140,465-482,586-593,657-659,685-691
nhTMEM16	4WIS	simulaion, Amber, c36m		128-140,465-482,586-593,657-659,685-691,
**nhTMEM16**	6QM4	Cryo-EM	fusarium vanettenii 77-13-4	1-14,417-424,586-594,651-664,685-687
**nhTMEM16**	6QM6	Cryo-EM	fusarium vanettenii 77-13-4	1-14,416-418,468-476,588-594,653-663,685-687
**nhTMEM16**	6QMA	Cryo-EM	fusarium vanettenii 77-13-4	1-14,417-424,469-476,586-594,651-664,685-687
**nhTMEM16**	6QMB	Cryo-EM	fusarium vanettenii 77-13-4	1-14,417-424,468-476,586-594,651-664,685-687
**afTMEM16**	7RXB	Cryo-EM	Aspergillus fumigatus	1-10,25-31,90-102,256-268,312-317,443-463, 594-603
**afTMEM16**	7RXG	Cryo-EM	Aspergillus fumigatus	1-11,725-735
**TMEM16K**	5OC9	X-ray diffraction, LCP	Homo sapiens	ASYMMETRIC 1-13A,57-66A,188-191A,350-351A, 472-474A,1-13B,188-191B, 348-353B
**TMEM16K**	6R7X	Cryo-EM	Homo sapiens	1-12
**TMEM16F**	6P47	Cryo-EM	Mus musculus	82-88,197-203,257-258,431-444,490-498,639-649
**TMEM16F**	6P48	Cryo-EM	Mus musculus	77-89,131-133,225-231
**TMEM16F**	6QPB	Cryo-EM	Mus musculus	80-89,491-501,633-645,791-792
**TMEM16F**	6QP6	Cryo-EM	Mus musculus	82-86,197-201,224-227,489-502,588-590,641-644,792-794
**TMEM16F**	6QPC	Cryo-EM	Mus musculus	108-114,258-263,639-646,765-771
**TMEM16F**	8TAG	Cryo-EM	Mus musculus	ASYMMETRIC 52-58A, 83-90A, 223-231A, 639-644A, 490-502B, 869-871B
**TMEM16F**	8B8G	Cryo-EM	Mus musculus	80-89,491-501,637-645,790-794
**TMEM16F**	8B8J	Cryo-EM	Mus musculus	82-88,120,221-232,489-502,641-644,792-794
**TMEM16F**	8B8Q	Cryo-EM	Mus musculus	82-88,120,221-232,639-644,789-794
**TMEM16F**	8B8K	Cryo-EM	Mus musculus	82-86,197-201,224-227,489-502,588-590,641-644,792-794
**TMEM16F**	8BC0	Cryo-EM	Mus musculus	ASYMMETRIC 79-90A, 220-232A,254-263A,588-590A, 640-644A,792-794A, 79-90B,120B,220-232B,254-263B,639-644B
TMEM16F	6QP6, simulated, OpenMM+c36m	Cryo-EM+simulation	Mus musculus	1–42, 150–186, 428–444, 489–502, 876–911
**TMEM16A**	5OYG	Cryo-EM	Mus musculus	260-266
**TMEM16A**	5OYB	Cryo-EM	Mus musculus	260-266,669-682
TMEM16A	5OYB - simulation, GROMACS and Amber, c36m	Cryo-EM	Mus musculus	260-266,467-487,669-682
TMEM16A	7ZK3 - simulated, cluster 6, Gromacs+c36m	Cryo-EM+simulation	Mus musculus	260-266,467-482,526-527,669-682
TMEM16A	7ZK3 - simulated, cluster 8, Gromacs+c36m	Cryo-EM+simulation	Mus musculus	260-266,467-482,526-527,669-682
TMEM16A	7ZK3 - simulated, cluster 10, Gromacs+c36m	Cryo-EM+simulation	Mus musculus	260-266,467-482,526-527,669-682

Table S1. TMEM16 structures selected for molecular dynamics (MD) simulations and modeled missing residues.

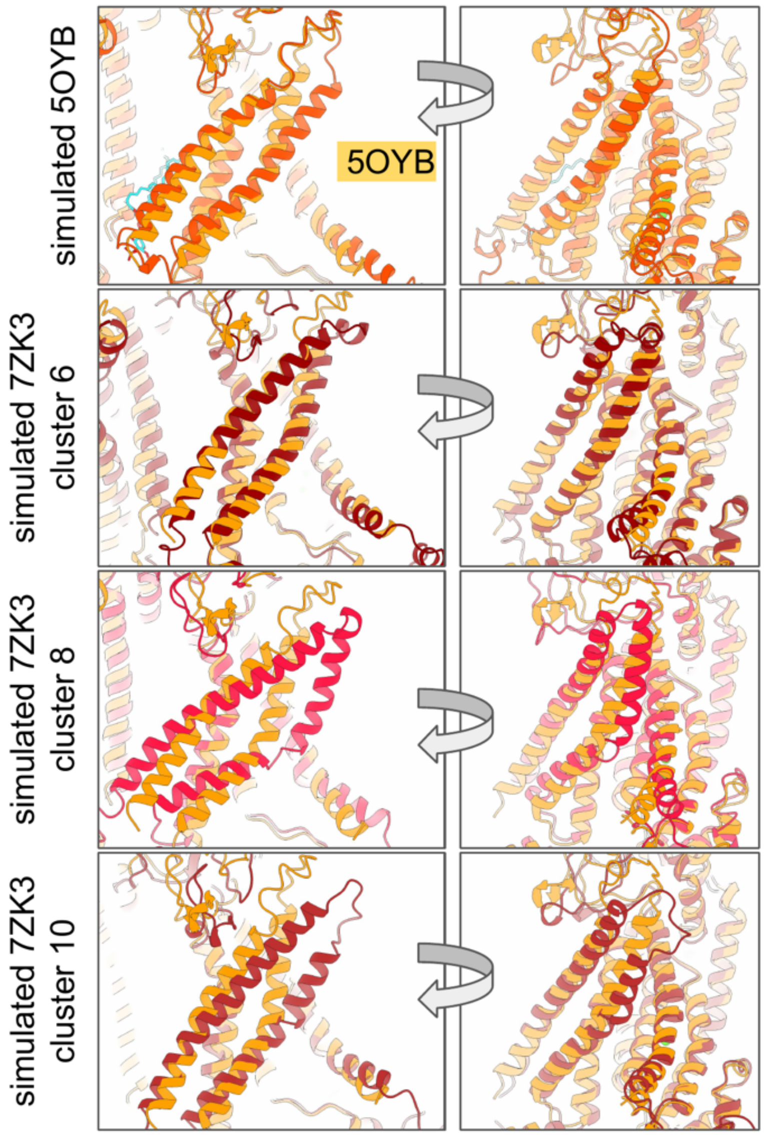

Figure S1. Predicted alternative and ion conductive states of TMEM16A. Starting coordinates for simulated conductive states of TMEM16A generated from atomistic MD simulations (darker colors). Each predicted open subunit is aligned to a Ca^{2+}-bound TMEM16A cryo-EM structure (PDB ID 5OYB, light orange).

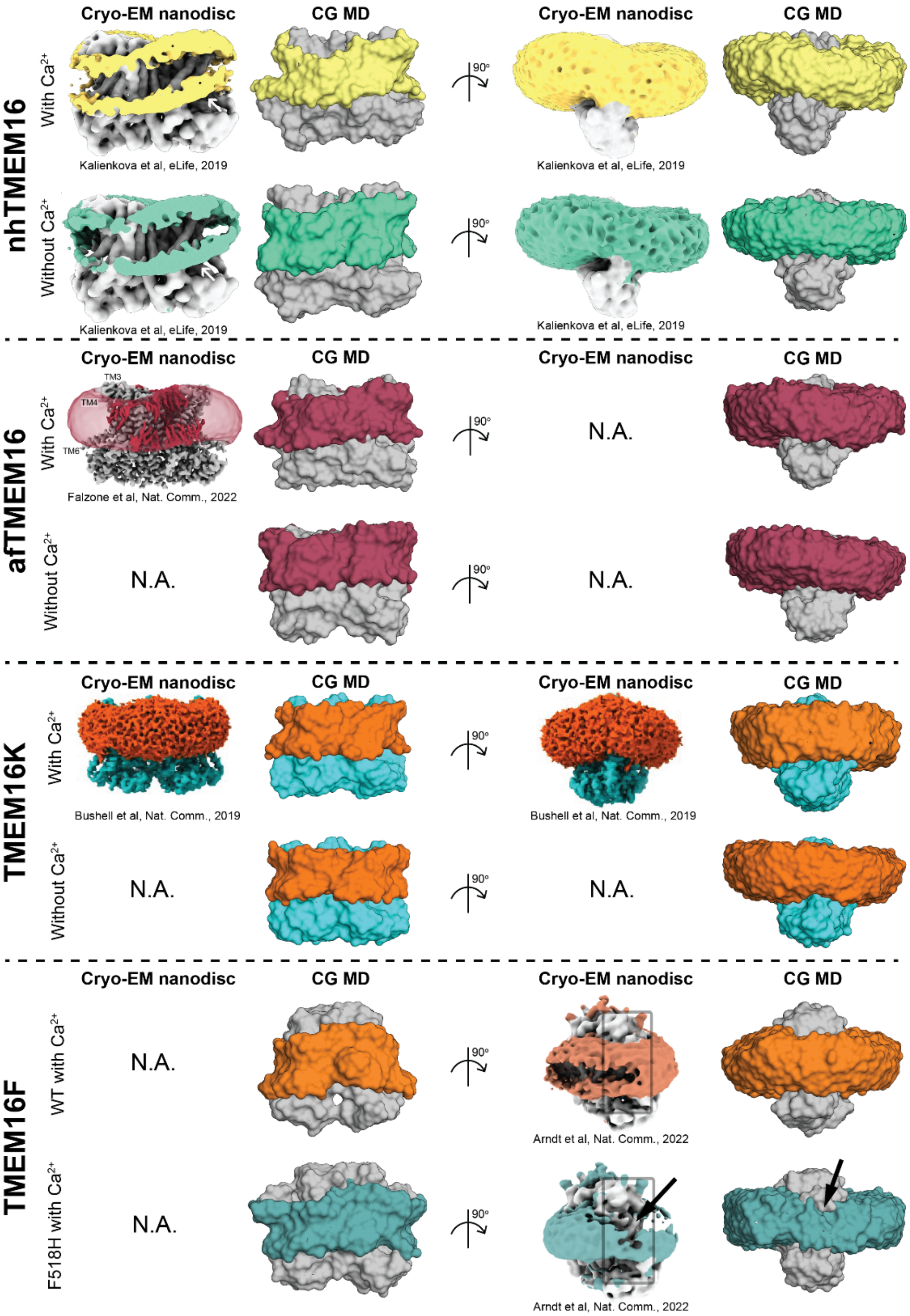

Figure S2. Comparison between membrane deformations in cryo-EM nanodiscs and CG MD. Front and side views of cryo-EM maps (left) and the final frames of our CG MD simulations (right). nhTMEM16: cryo-EM images (PDB IDs 6QM9 and 6QM4) were adapted from © 2019, Kalienkova *et al*, published by eLife (1). The CG MD structures are PDB IDs 4WIS (with Ca^{2+}) and 6QM4 (without Ca^{2+}). afTMEM16: cryo-EM image (PDB ID 7RXG) was adapted from © 2022, Falzone *et al*, published by Springer Nature (2). The CG MD structures are PDB IDs 7RXG (with Ca^{2+}) and 7RXB (without Ca^{2+}). TMEM16K: cryo-EM images (PDB ID 5OC9) were adapted from © 2019, Bushell *et al*, published by Springer Nature (3). The CG MD structures are PDB IDs 5OC9 (with Ca^{2+}) and 6R7X (without Ca^{2+}). TMEM16F: cryo-EM images (PDB IDs 6QPC and 8B8J) were adapted from © 2022, Arndt *et al*, published by Springer Nature (4). The CG MD structures are also PDB IDs 6QPC (WT with Ca^{2+}) and 8B8J (F518H with Ca^{2+}). Black arrows indicate a dip in lipid density near the groove entrance. All CG MD snapshots were rendered with PyMOL 2.5.0 (5), after selecting all lipid beads within 12 Å of the protein and matching the coloring to the colors used in the original cryo-EM images.

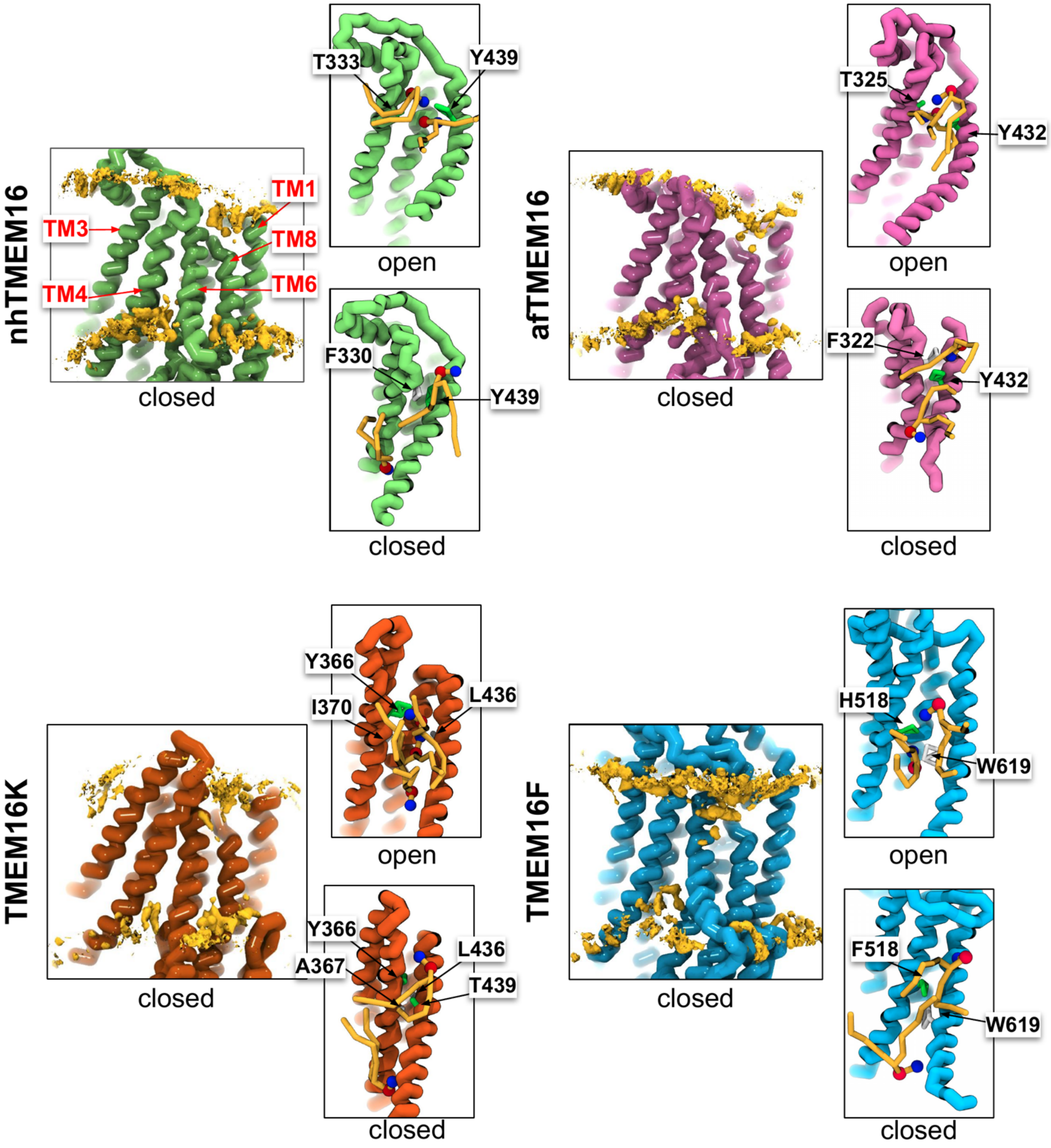

Figure S3. CG simulations of multiple TMEM16 structures with closed grooves lack lipid density in the TM4/TM6 pathway. Snapshots from CG MD simulations of nhTMEM16 (PDB IDs 6QM4 (closed) and 4WIS (open), green), afTMEM16 (PDB IDs 7RXB (closed) and 7RXG (open), violet), TMEM16K (PDB IDs 6R7X (closed) and 5OC9 (open), gold), and TMEM16F (PDB IDs 6QPB (closed) and 6QP6* (open), blue) with phosphatidylcholine (PC) lipid headgroup density (yellow) and nearby lipids (yellow). Residues forming the closest distance between TM4 and TM6 (colored by residue type: basic (red), acidic (blue), and polar (green)) and lipids near the groove also shown. Each density is averaged over both chains.

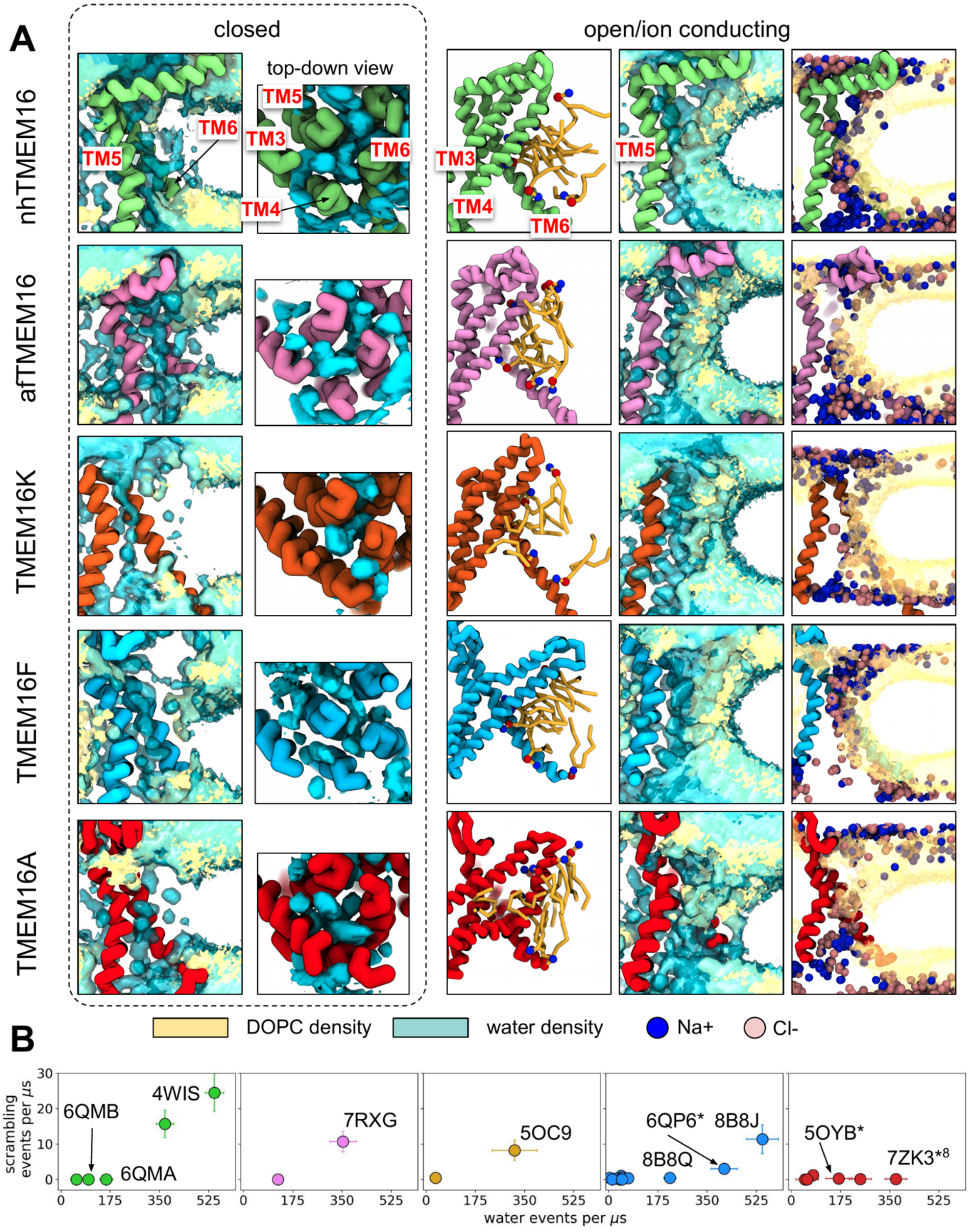

Figure S4. Simulated lipid scrambling correlates with water permeation through the TM4/TM6 groove. (**A**) Water and lipid headgroup density in CG simulations of closed (**left**) and open/ion conducting (**right**) structures: nhTMEM16 (PDB IDs 6QM4 (closed) and 4WIS (open), green), afTMEM16 (PDB IDs 7RXB (closed) and 7RXG (open), violet), TMEM16K (PDB IDs 6R7X (closed) and 5OC9 (open), gold), and TMEM16F (PDB IDs 6QPB (closed) and 8B8J (open), blue). Ions positions (cyan and green beads) shown every 100 frames. TM4 not shown in the first and two rightmost columns. Density is shown at the same isovalue contour for all images. (**B**) The number of scrambling events plotted against the average number of water permeation events per μs. Due to the presence of asymmetric structures the maximum water permeation rate of the two subunits is shown. Water beads were tracked within a 9.4 Å radius of the maximum density pathway (see Methods). Density is shown at the same isovalue contour for all images.

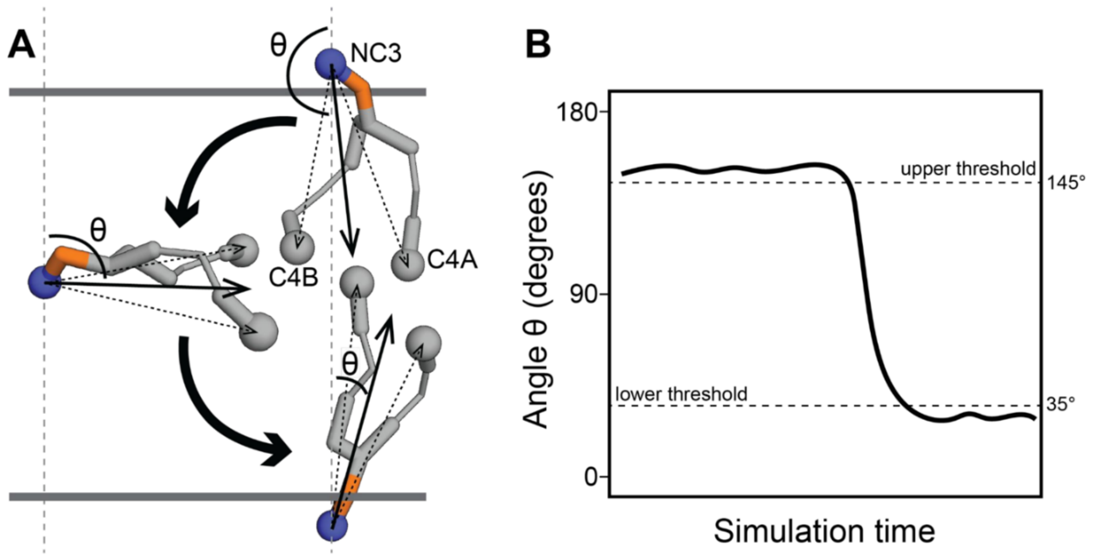

Figure S5. Measuring lipid angles to detect scrambling events. (A) For every time frame, for every lipid in the system, we defined a vector between the choline bead (NC3) and the two tail beads (C4A, C4B; dashed arrows) and calculated the angle θ between the average of those two vectors (solid arrow) with the z axis. **(B)** A schematic representation of a typical time trace for a lipid that scrambles from the upper membrane leaflet (θ ≈ 150°) to the lower membrane leaflet (θ ≈ 30°). A scrambling event is only counted when θ passes the threshold at the opposite leaflet with respect to its original location (35° for the lower, 145° for the upper).

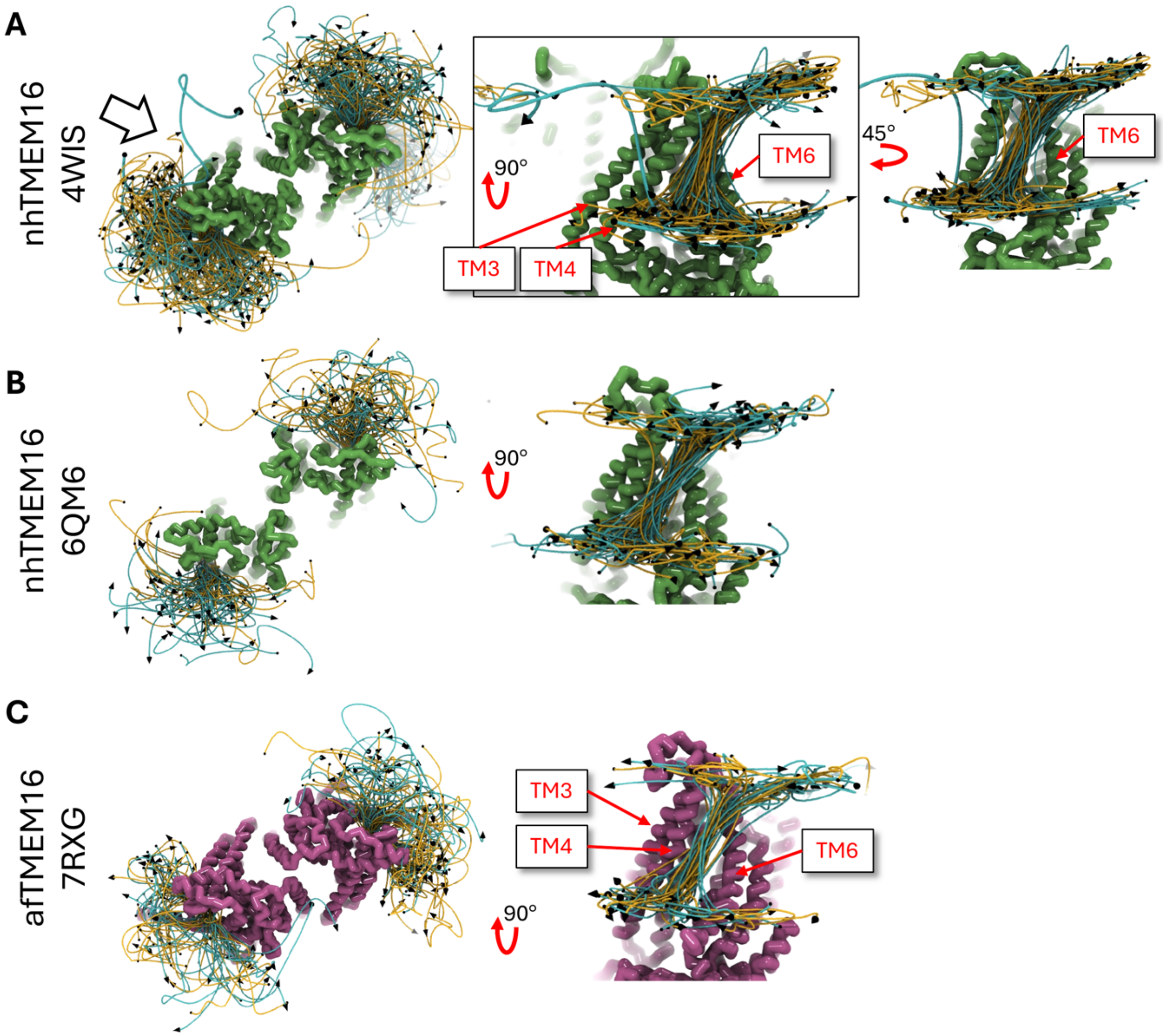

Figure S6. Position traces of scrambling lipids in fungal TMEM16 simulations. (A) Lipid traces for Ca^{2+}-bound open nhTMEM16 (PDB ID 4WIS). **(B)** Lipid traces for Ca^{2+}-bound open nhTMEM16 (PDB ID 6QM6). **(C)** Lipid traces for Ca^{2+} -bound open afTMEM16 (PDB ID 7RXG). Lipid traces are generated by fitting raw lipid headgroup center of mass positions to a smooth spline curve.

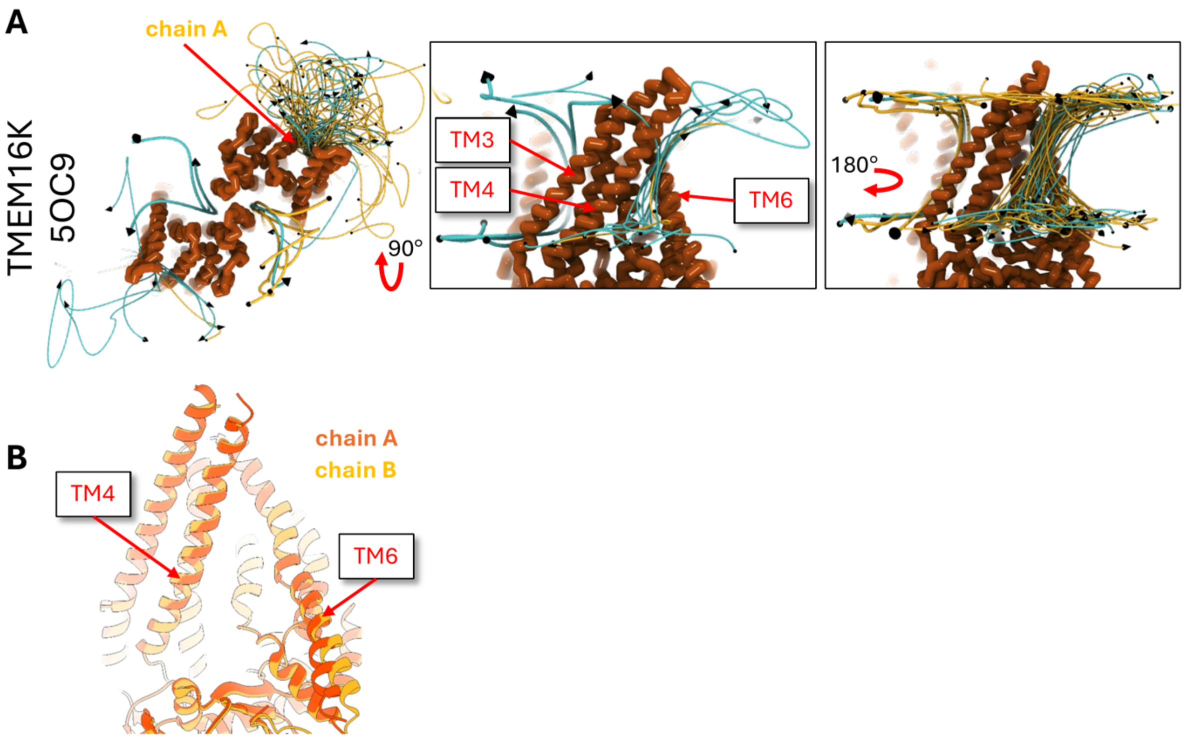

Figure S7. Position traces of scrambling lipids in the open TMEM16K simulation. (A) Lipid traces for Ca^{2+}-bound open TMEM16K (PDB ID 5OC9). **(B)** Cartoon representation of aligned subunits of Ca^{2+}-bound TMEM16K (PDB ID 5OC9). Lipid traces are generated by fitting raw lipid headgroup center of mass positions to a smooth spline curve.

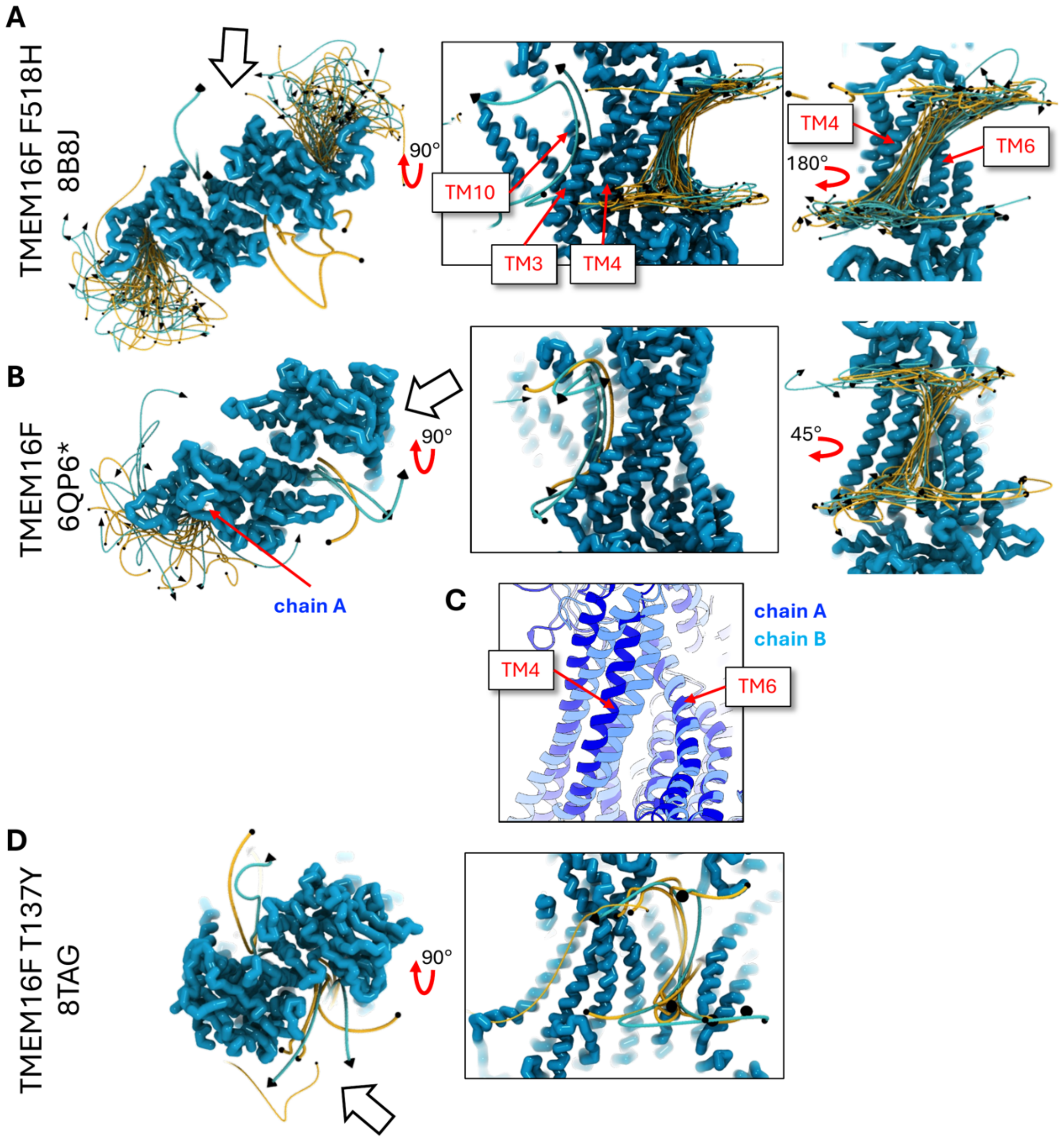

Figure S8. Position traces of scrambling lipids in TMEM16F simulations. (A) Lipid traces for Ca^{2+} -bound open TMEM16F F518H mutant (PDB ID 8B8J). **(B)** Lipid traces for Ca^{2+} -bound simulated open TMEM16F (6QP6*) **(C)** Cartoon representation of aligned subunits of Ca^{2+}-bound simulated open TMEM16F (6QP6*). **(D)** Lipid traces for Ca^{2+} -bound simulated closed TMEM16F (PDB ID 8TAG). Lipid traces are generated by fitting raw lipid headgroup center of mass positions to a smooth spline curve.

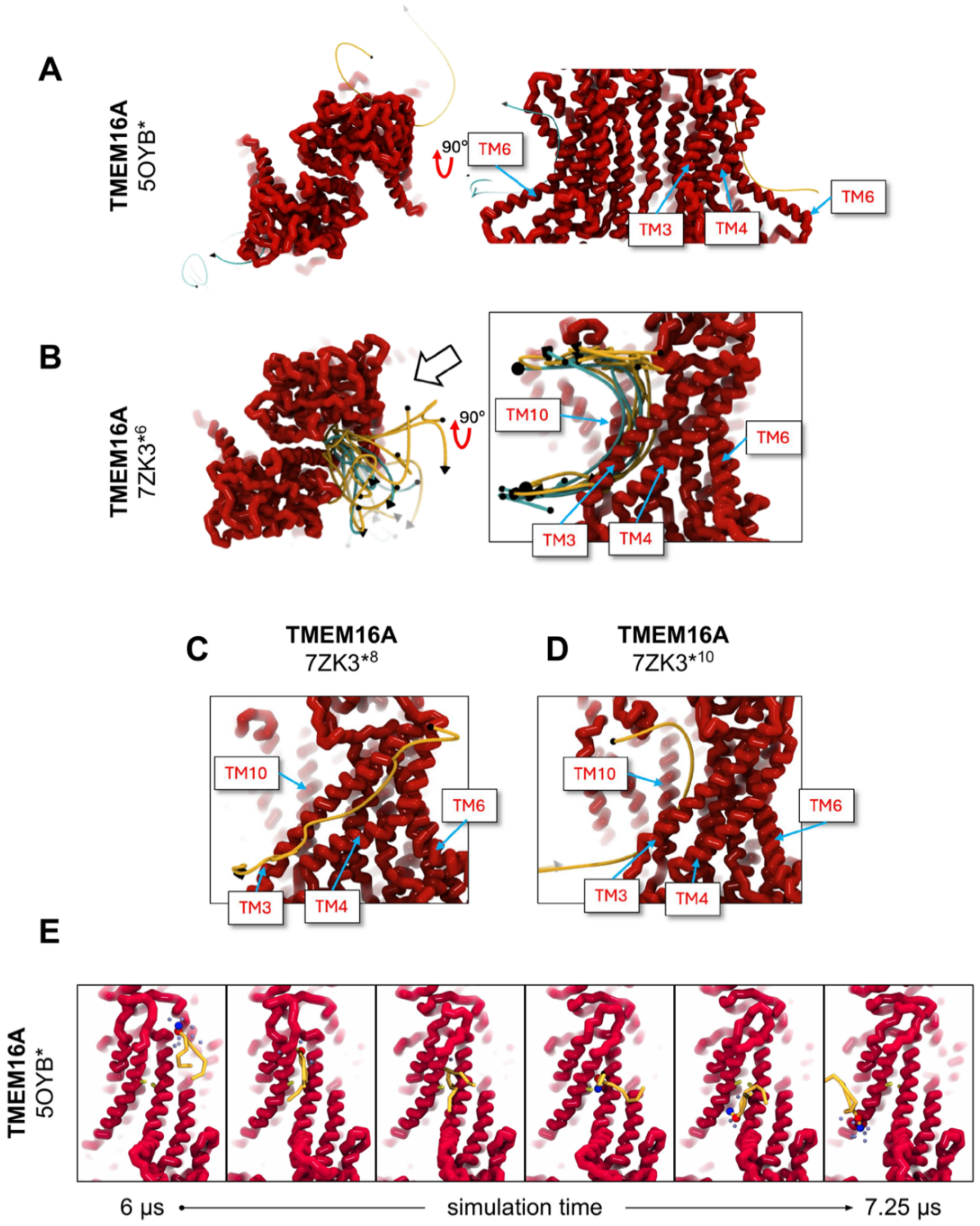

Figure S9. Position traces of scrambling lipids in TMEM16A simulations. (A) Lipid traces for Ca^{2+} -bound simulated conductive TMEM16A (5OYB*). **(B)** Lipid traces for Ca^{2+} -bound simulated open TMEM16A (7ZK3*[6]). **(C)** Lipid traces for Ca^{2+} -bound simulated open TMEM16A (7ZK3*[10]). **(D)** Lipid traces for Ca^{2+} -bound simulated open TMEM16A (7ZK3*[8]). **(E)** Snapshots from simulation of 5OYB* during lipid scrambling event (trace in **A**). Lipid traces are generated by fitting raw lipid headgroup center of mass positions to a smooth spline curve.

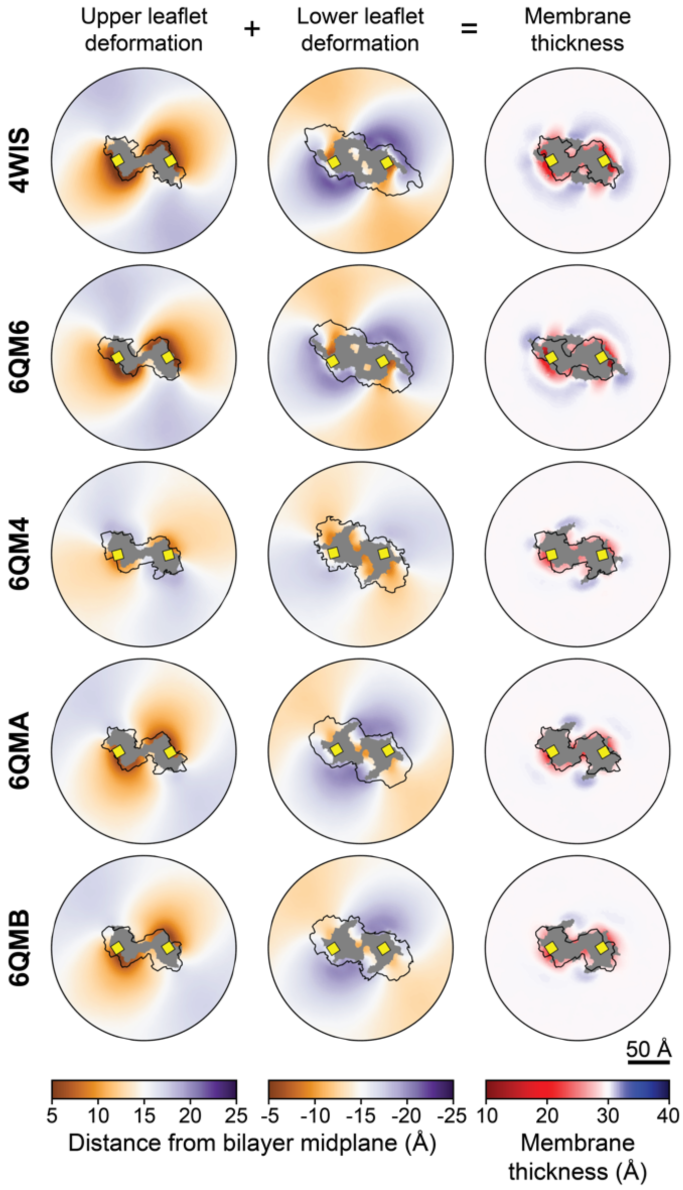

Figure S10. Membrane deformations for simulated nhTMEM16 structures. Left column: xy-map of the distance along the z-axis from the bilayer midplane to the ensemble averaged positions of the glycerol linker (GL1 and GL2 beads). Middle column: xy-map of the distance along the z-axis from the bilayer midplane to the ensemble averaged positions of the glycerol linker (GL1 and GL2 beads). Right column: the sum of the upper and lower leaflet deformations, representing the bilayer thickness along z. In all plots, grey areas indicate grid points with lipid occupancy <2%. The black outline is the projected surface of the upper (z>0) or lower (z<0) portion of the protein dimer.

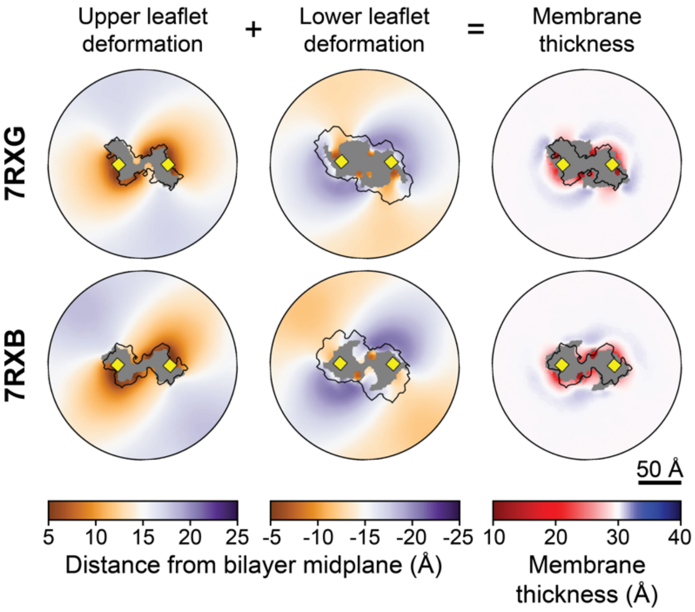

Figure S11. Membrane deformations for simulated afTMEM16 structures. Left column: xy-map of the distance along the z-axis from the bilayer midplane to the ensemble averaged positions of the glycerol linker (GL1 and GL2 beads). Middle column: xy-map of the distance along the z-axis from the bilayer midplane to the ensemble averaged positions of the glycerol linker (GL1 and GL2 beads). Right column: the sum of the upper and lower leaflet deformations, representing the bilayer thickness along z. In all plots, grey areas indicate grid points with lipid occupancy <2%. The black outline is the projected surface of the upper (z>0) or lower (z<0) portion of the protein dimer.

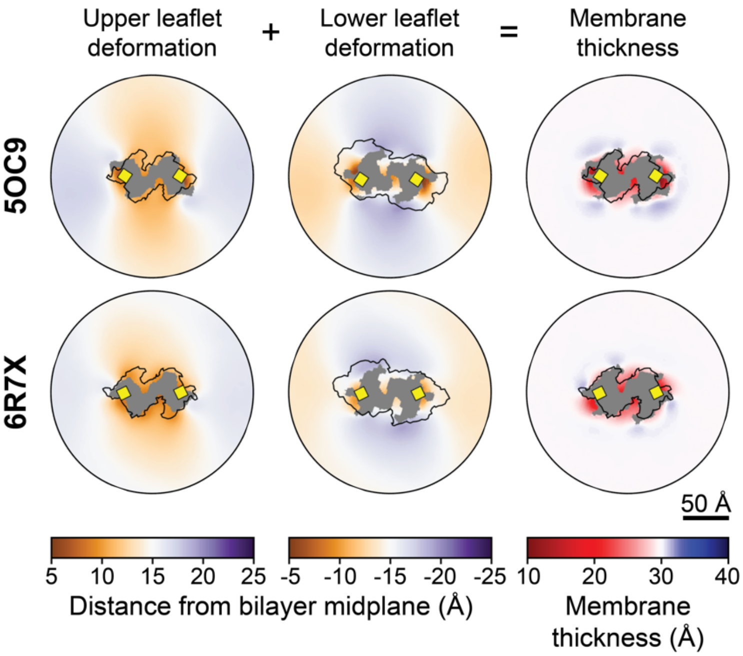

Figure S12. Membrane deformations for simulated TMEM16K structures. Left column: xy-map of the distance along the z-axis from the bilayer midplane to the ensemble averaged positions of the glycerol linker (GL1 and GL2 beads). Middle column: xy-map of the distance along the z-axis from the bilayer midplane to the ensemble averaged positions of the glycerol linker (GL1 and GL2 beads). Right column: the sum of the upper and lower leaflet deformations, representing the bilayer thickness along z. In all plots, grey areas indicate grid points with lipid occupancy <2%. The black outline is the projected surface of the upper (z>0) or lower (z<0) portion of the protein dimer.

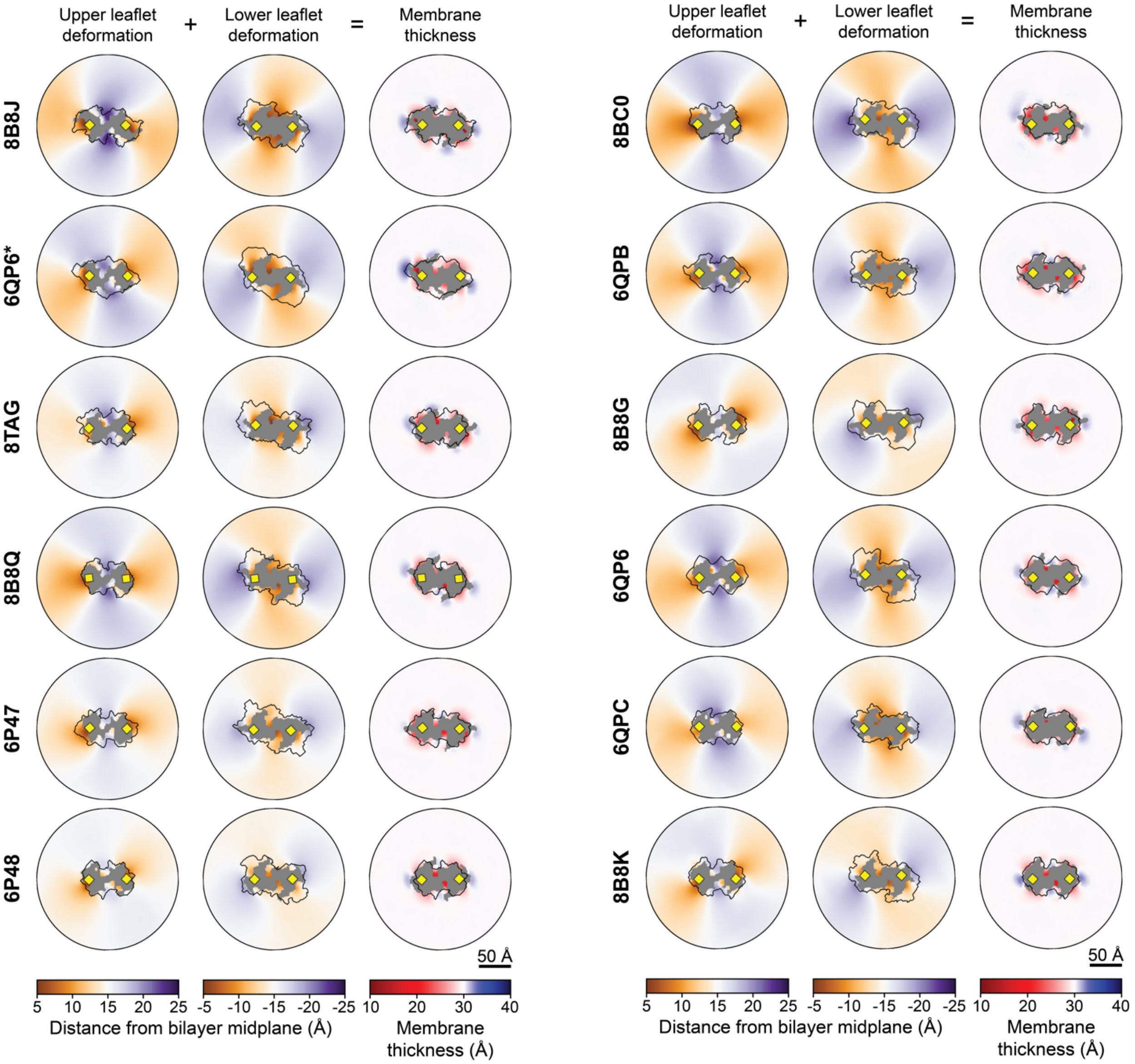

Figure S13. Membrane deformations for simulated TMEM16F structures. Left column: xy-map of the distance along the z-axis from the bilayer midplane to the ensemble averaged positions of the glycerol linker (GL1 and GL2 beads). Middle column: xy-map of the distance along the z-axis from the bilayer midplane to the ensemble averaged positions of the glycerol linker (GL1 and GL2 beads). Right column: the sum of the upper and lower leaflet deformations, representing the bilayer thickness along z. In all plots, grey areas indicate grid points with lipid occupancy <2%. The black outline is the projected surface of the upper (z>0) or lower (z<0) portion of the protein dimer.

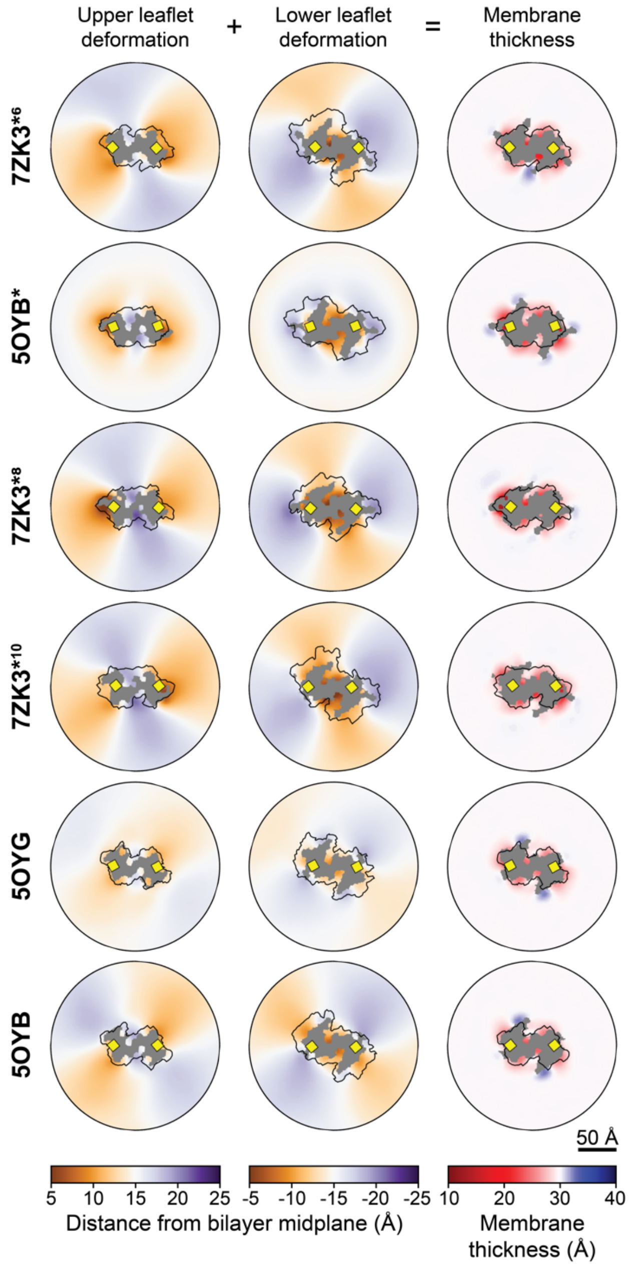

Figure S14. Membrane deformations for simulated TMEM16A structures. Left column: xy-map of the distance along the z-axis from the bilayer midplane to the ensemble averaged positions of the glycerol linker (GL1 and GL2 beads). Middle column: xy-map of the distance along the z-axis from the bilayer midplane to the ensemble averaged positions of the glycerol linker (GL1 and GL2 beads). Right column: the sum of the upper and lower leaflet deformations, representing the bilayer thickness along z. In all plots, grey areas indicate grid points with lipid occupancy <2%. The black outline is the projected surface of the upper (z>0) or lower (z<0) portion of the protein dimer.

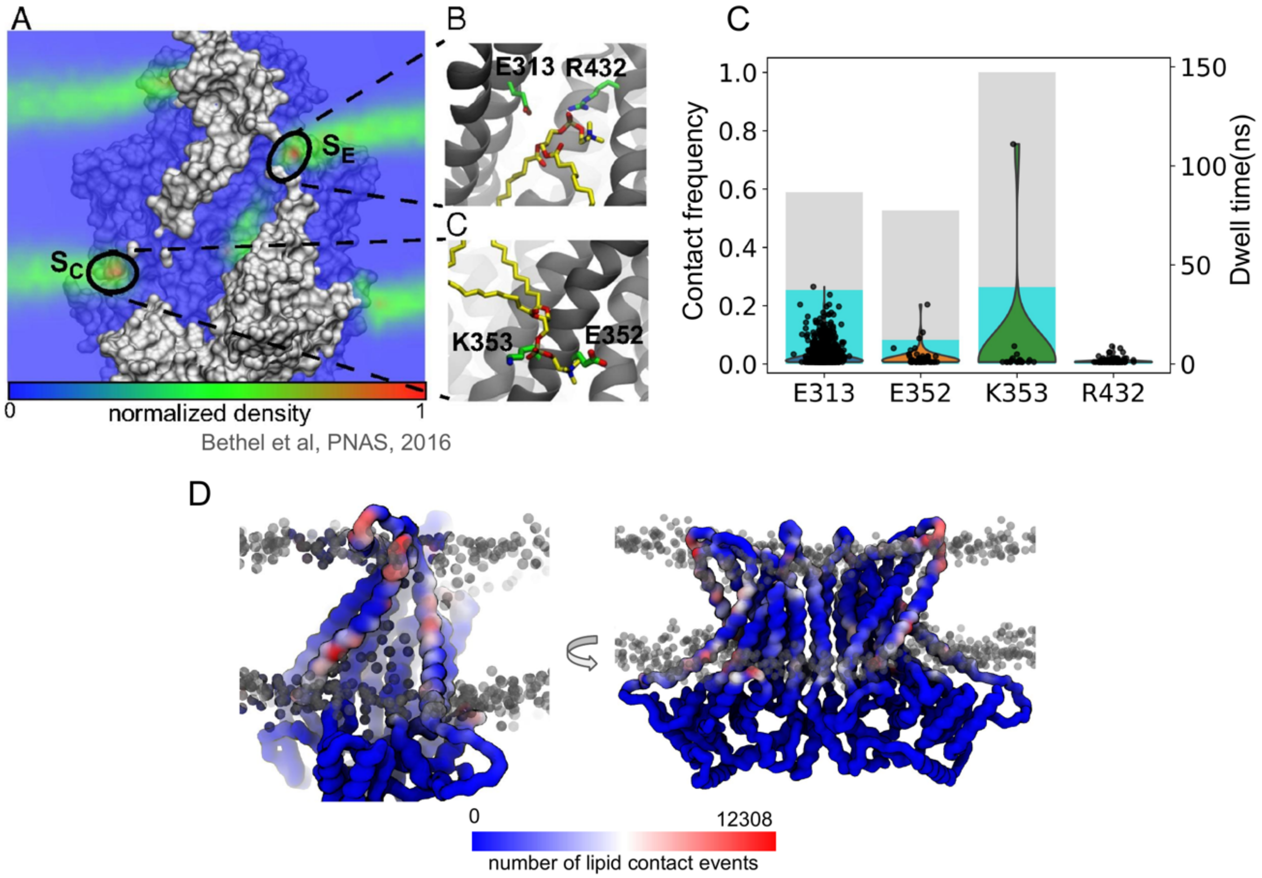

Figure S15. Contact analysis of lipid headgroup high density sites identified from previous AA simulation. (A-C) Charged residues and nearby POPC lipids near two high lipid phosphate density sites at the intracellular and extracellular entry of the open nhTMEM16 (PDB ID 4WIS) canonical groove ("S_C", "S_E") identified from previous AA simulation © 2016, Bethel & Grabe, published by *PNAS* (6). **(C)** Contact frequency with any lipid (grey bars) and only scrambling lipids (cyan bars). Dwell times with scrambling lipids shown as black points. **(D)** The nhTMEM16 CG backbone colored by total number of contact events with any lipid. Grey spheres indicate lipid headgroup positions in a single snapshot.

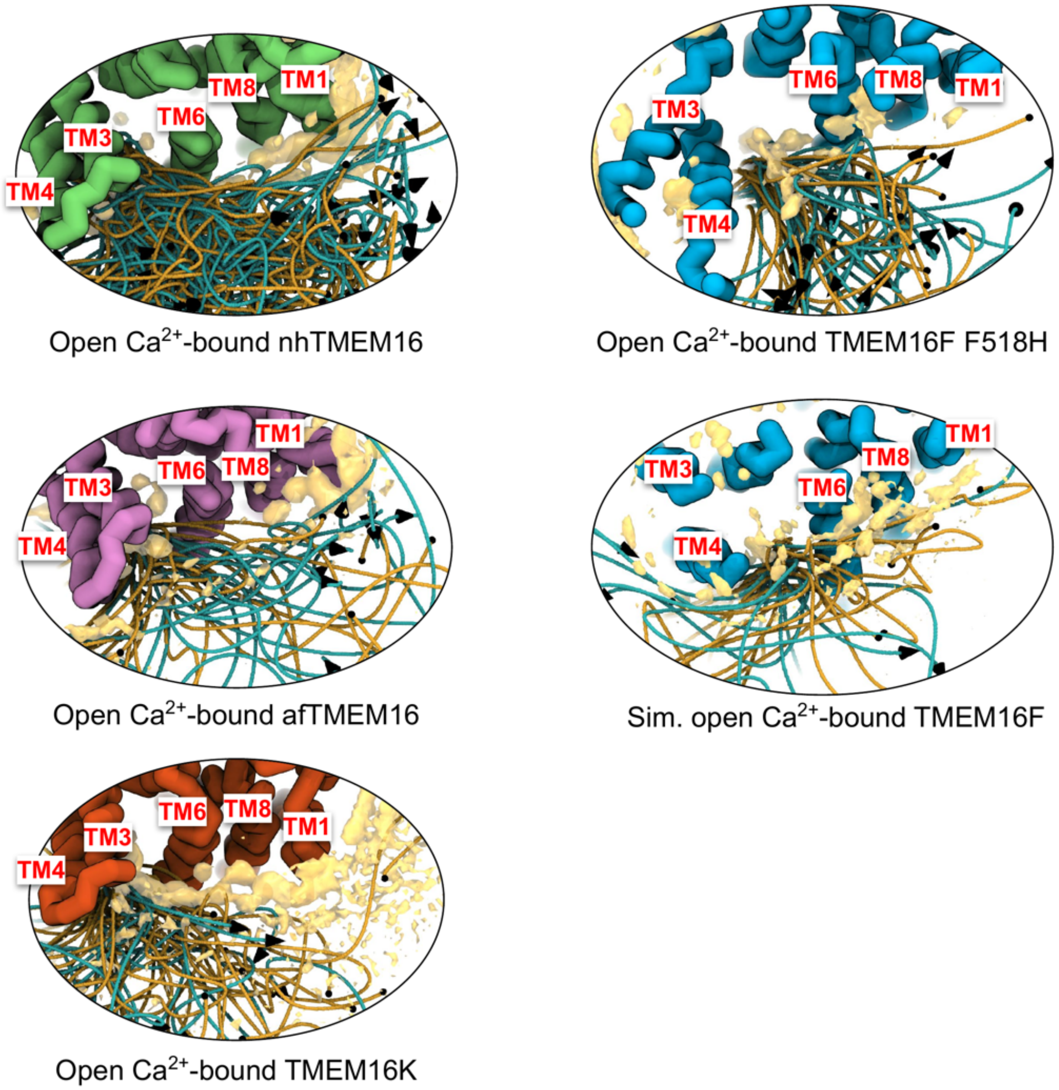

Figure S16. Position traces of scrambling lipids with total lipid headgroup density. Lipid traces and lipid headgroup density (yellow) for Ca^{2+} -bound scrambling competent TMEM16s: nhTMEM16 (PDB ID 4WIS, green), afTMEM16 (PDB ID 7RXG, purple), TMEM16K (PDB ID 5OC9, orange), TMEM16F F518H (PDB ID 8B8J, blue) and simulated TMEM16F (6QP6*). Each image is viewed from the extracellular or cytosolic (TMEM16K) space. Lipid traces are generated by fitting raw lipid headgroup center of mass positions to a smooth spline curve.

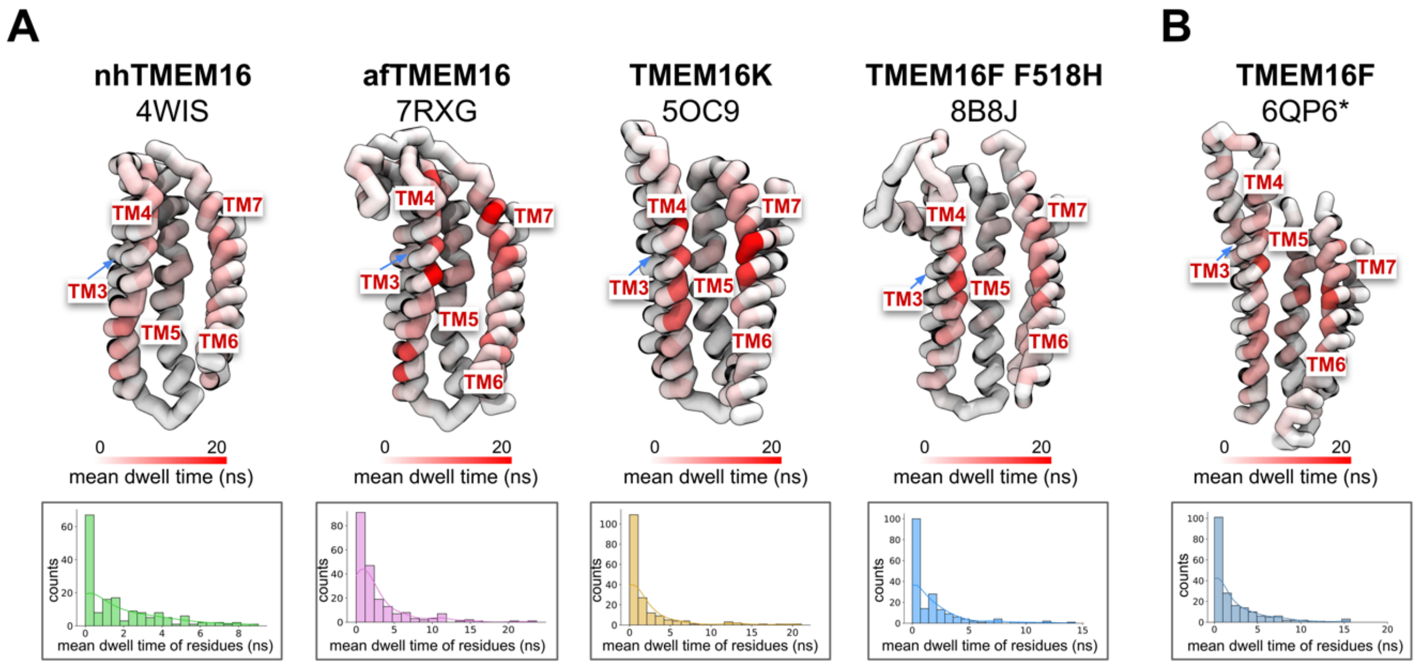

Figure S17. Average duration of interaction between scrambling lipids and TM4/TM6 groove lining residues. The canonical groove of each experimentally solved **(A)** and simulated **(B)** open scramblase structure is colored by the average duration of each interaction (dwell time) between scrambling lipids and groove lining residues. The distribution of average dwell times at individual residues is shown as a histogram below each structure.

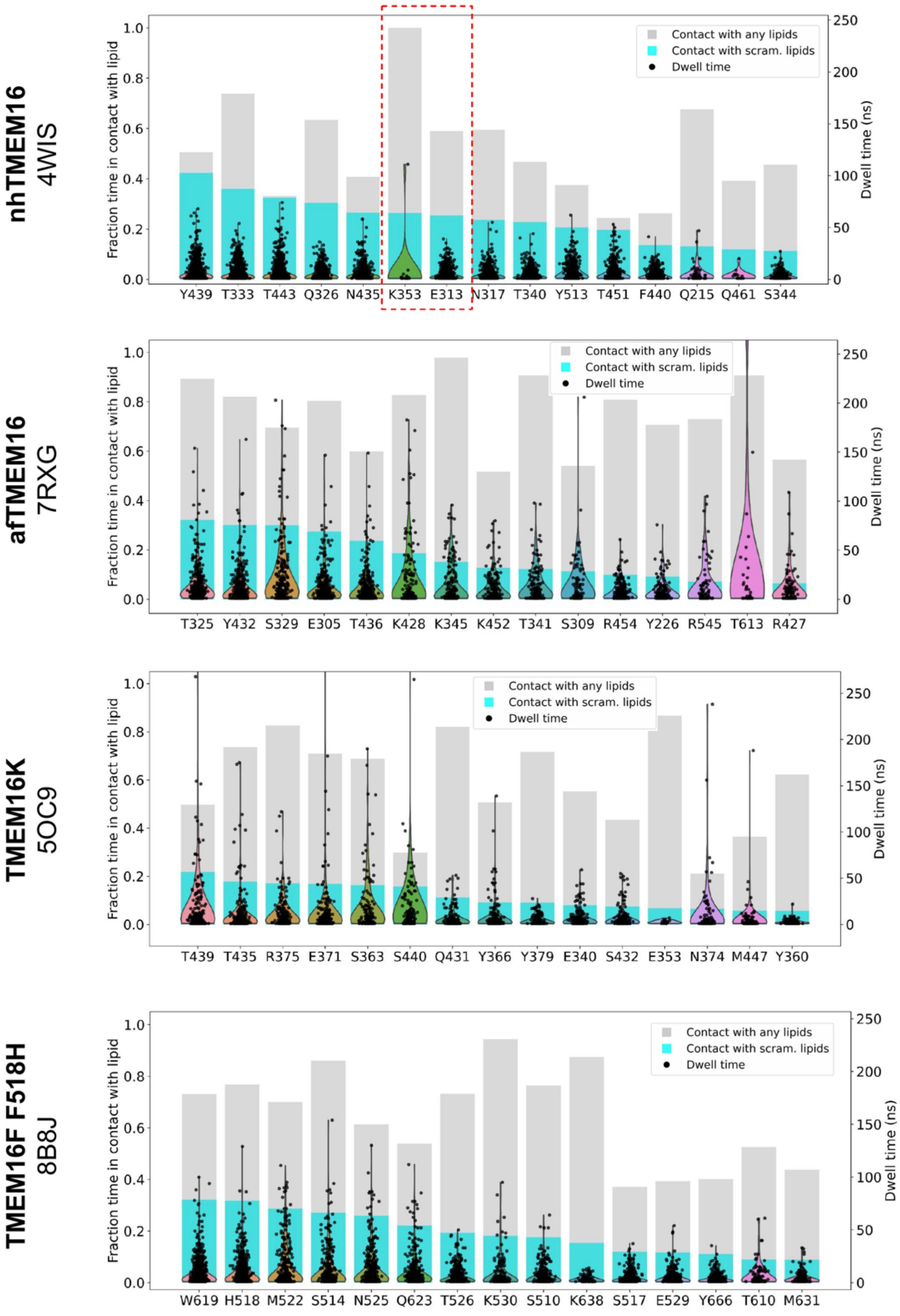

Figure S18. Dwell time distribution and contact frequency for TM4/TM6 groove lining residue across homologs. Contact frequency (cyan bar, left y-axis) and distribution of interaction dwell times (black scatter dots, right y-axis) between scrambling lipids and canonical groove lining residues with the 15 longest average interaction dwell times. Residues are sorted by the contact frequency. Frequency of contact with any lipid (scrambling and non-scrambling lipids taken together) is shown as grey bar. The red dashed-line rectangle indicates two previously identified residues near high lipid phosphate density in an all-atom (AA) simulation (6).

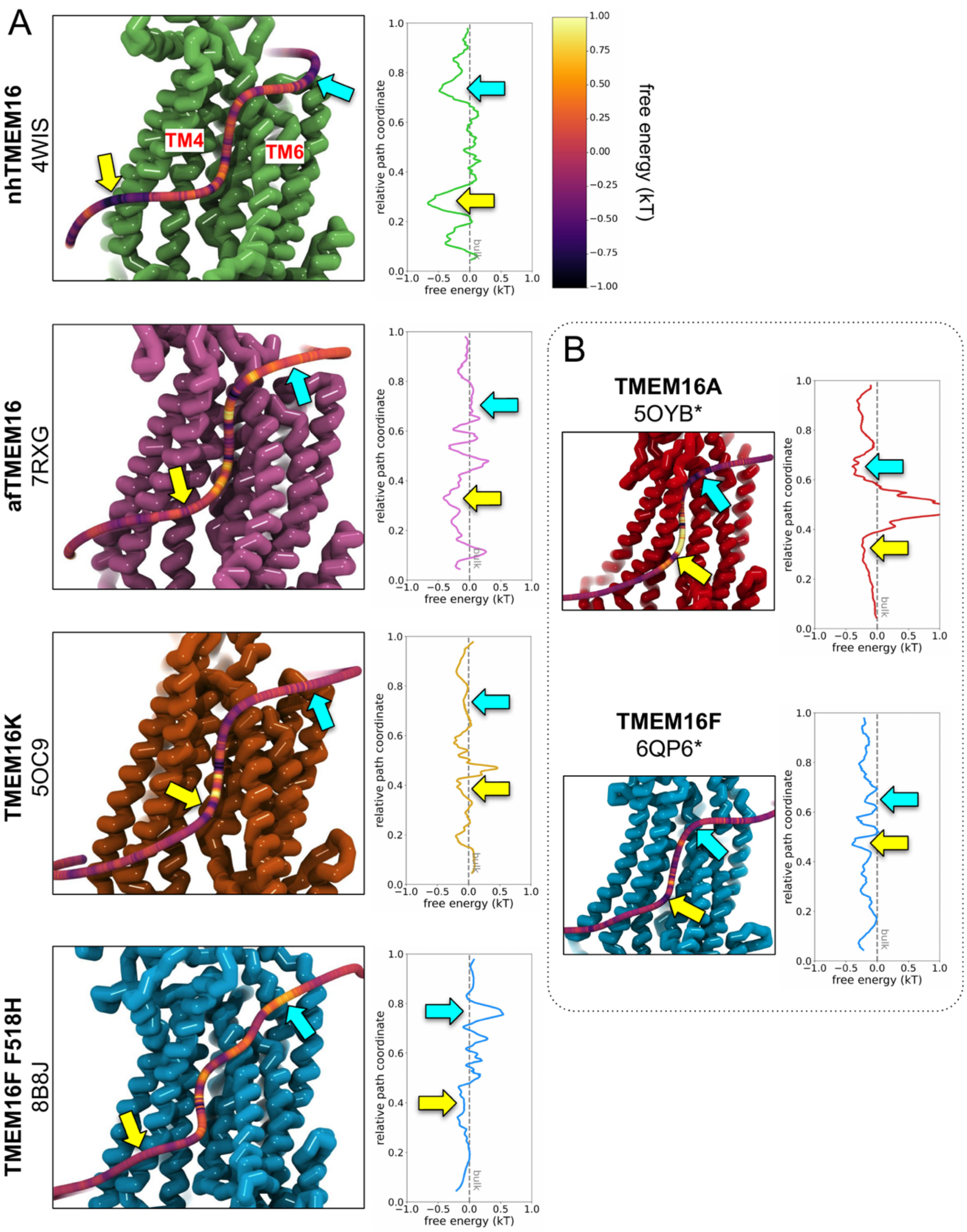

Figure S19. PMF estimates from DOPC headgroup density. (A) Left: Snapshots of nhTMEM16 (PDB ID 4WIS, green), afTMEM16 (PDB ID 7RXG, violet), TMEM16K (PDB ID 5OC9, orange), and TMEM16F F518H (PDB ID 8B8J, blue), overlaid with a 3D maximum density path (see methods) calculated from DOPC headgroup positions averaged from both subunits, except for asymmetric TMEM16K and WT TMEM16F paths on subunit with higher scrambling events. Each path is colored by the free energy (kT) calculated from the total density within 4.7 Å of a given node on the path normalized by the lipid headgroup density from simulation of a protein-free DOPC bilayer. Right: the same PMF plotted against its relative position along the maximum density pathway. Colored arrows indicate the same locations along the path. **(B)** PMF-colored 3D maximum lipid density paths for simulated ion conducting TMEM16A (5OYB*, red) and a simulation open WT TMEM16F (6QP6*, blue) simulation with corresponding PMF curve (right).

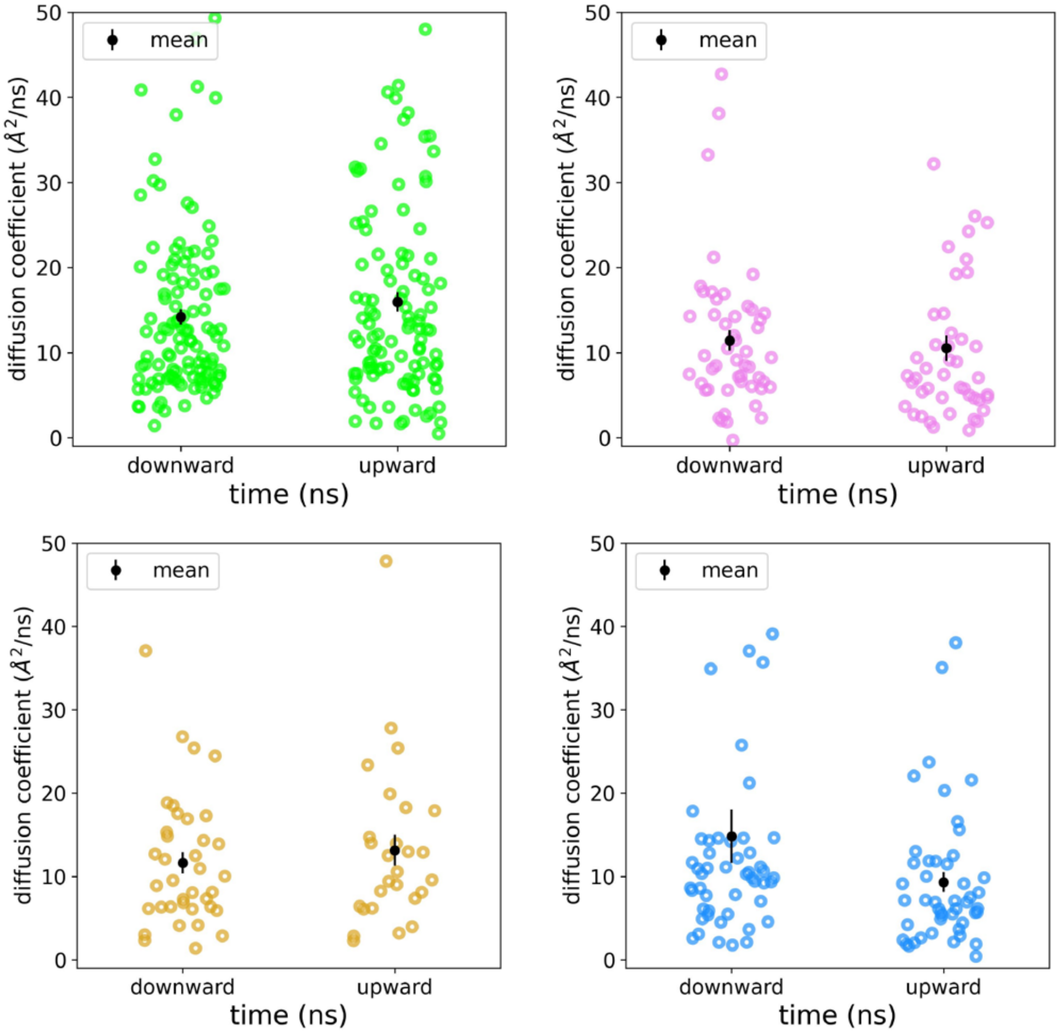

Figure S20. Diffusion coefficients for scrambling lipids. Diffusion coefficients were fit to individual mean squared displacement curves for individual DOPC lipids during their scrambling event in simulations of nhTMEM16 (PDB ID 4WIS, green), afTMEM16 (PDB ID 7RXG, violet), TMEM16K (PDB ID 5OC9, gold), and TMEM16F (8B8J, blue). The mean with standard deviation shown in black.

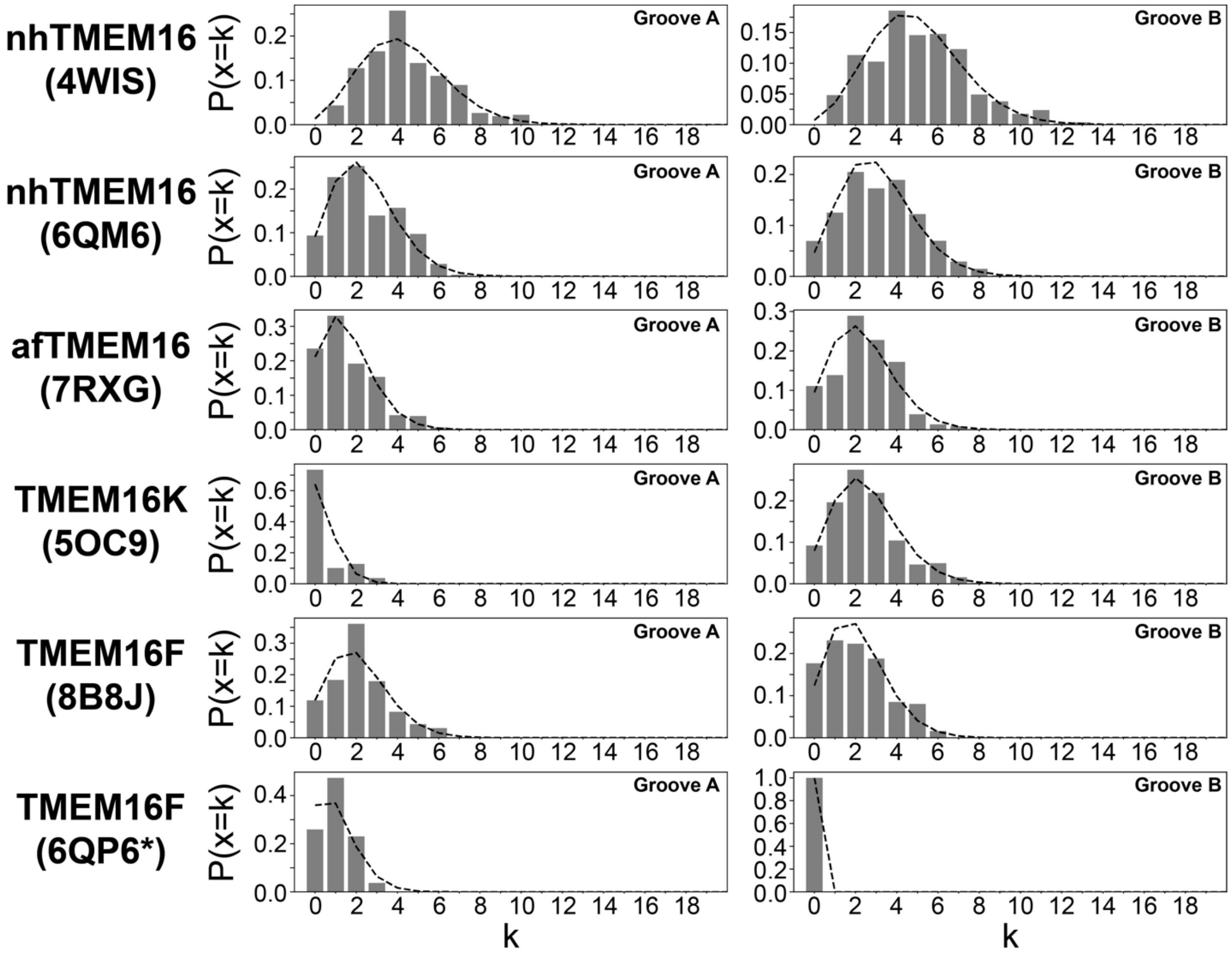

Figure S21. Scrambling events are Poisson distributed. Poisson distributions calculated on 400 ns intervals for all scrambling events in groove A (left) and groove B (right) of scrambling-competent structures. Bars represent data, dashed line is the theoretical distribution $P(x=k) = \lambda^k e^{-\lambda}/k!$, with λ being the average number of events in the 400 ns interval.

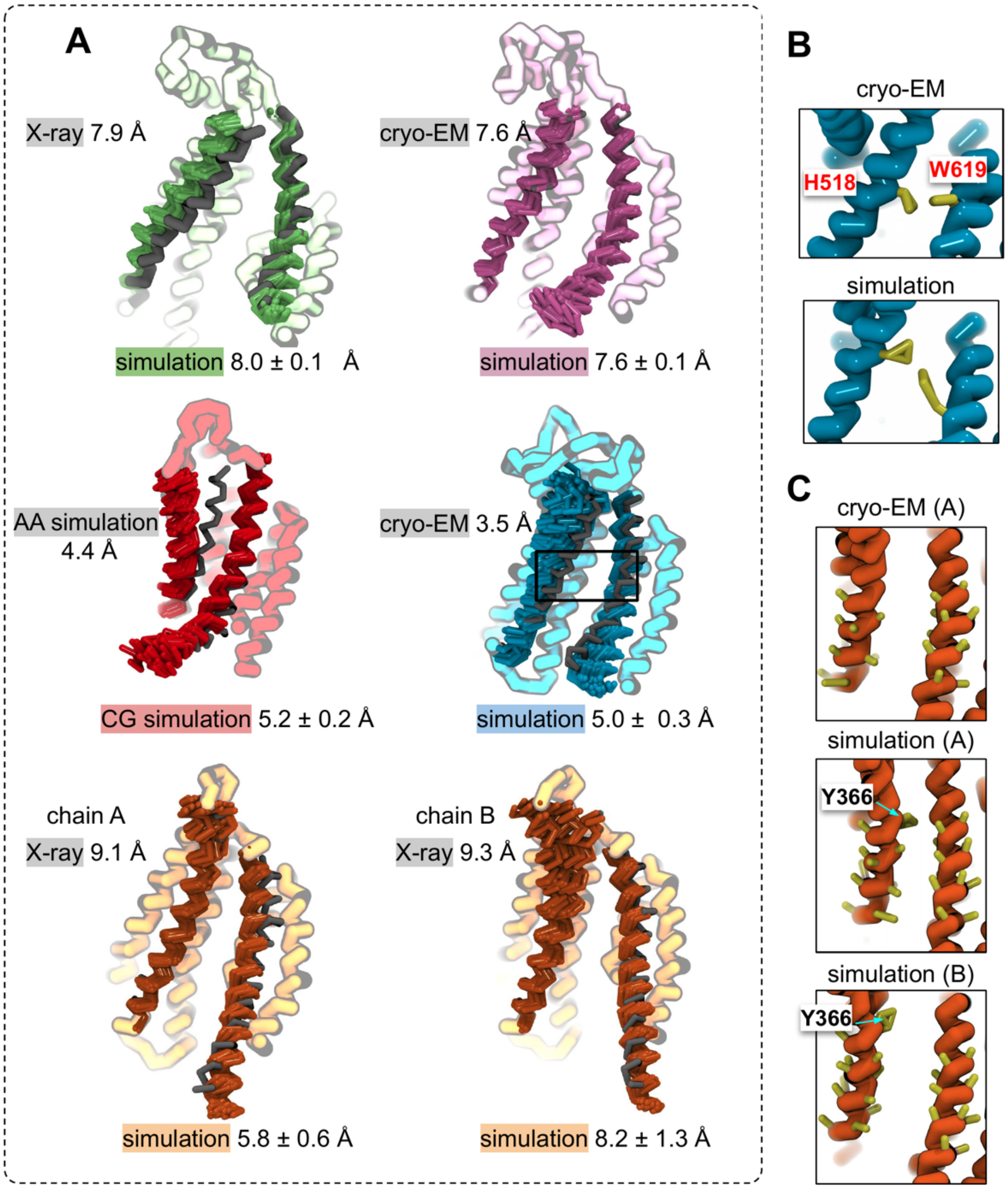

Figure S22. TM4 moves away from starting structure coordinates in open states. (A) Overlayed CG representations of experimentally determined or simulated starting structures and snapshots from CG simulations. nhTMEM16 4WIS (green), afTMEM16 7RXG (violet), TMEM16K 5OC9 (orange), TMEM16F 8B8J (blue), and TMEM16A 5OYB* (red). (**B)** TMEM16F TM4/TM6 groove constriction point at residues F518H (on TM4) and W619 (on TM6). **C)** TMEM16K TM4/TM6 groove constriction point at Y366 (on TM4).

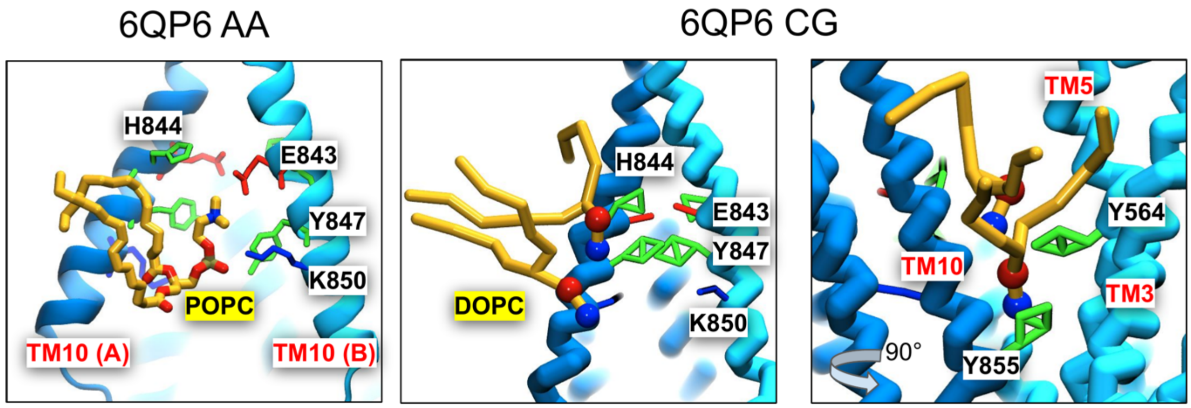

Figure S23. Lipids enter the dimer interface in atomistic and CG simulations of TMEM16F. Left: a snapshot of a 1-palmitoyl-2-oleoyl-glycero-3-phosphocholine (POPC) lipid that entered the TMEM16F (PDB ID 6QP6) dimer interface from the outer leaflet during an all-atom (AA) simulation. Right: snapshots at the same timepoint of 1,2-dioleoyl-sn-glycero-3-phosphocholine (DOPC) lipids that entered the TMEM16F (PDB ID 6QM6) dimer interface during a CG simulation. Nearby side chains are colored by residue type: basic (red), acidic (blue), and polar (green).

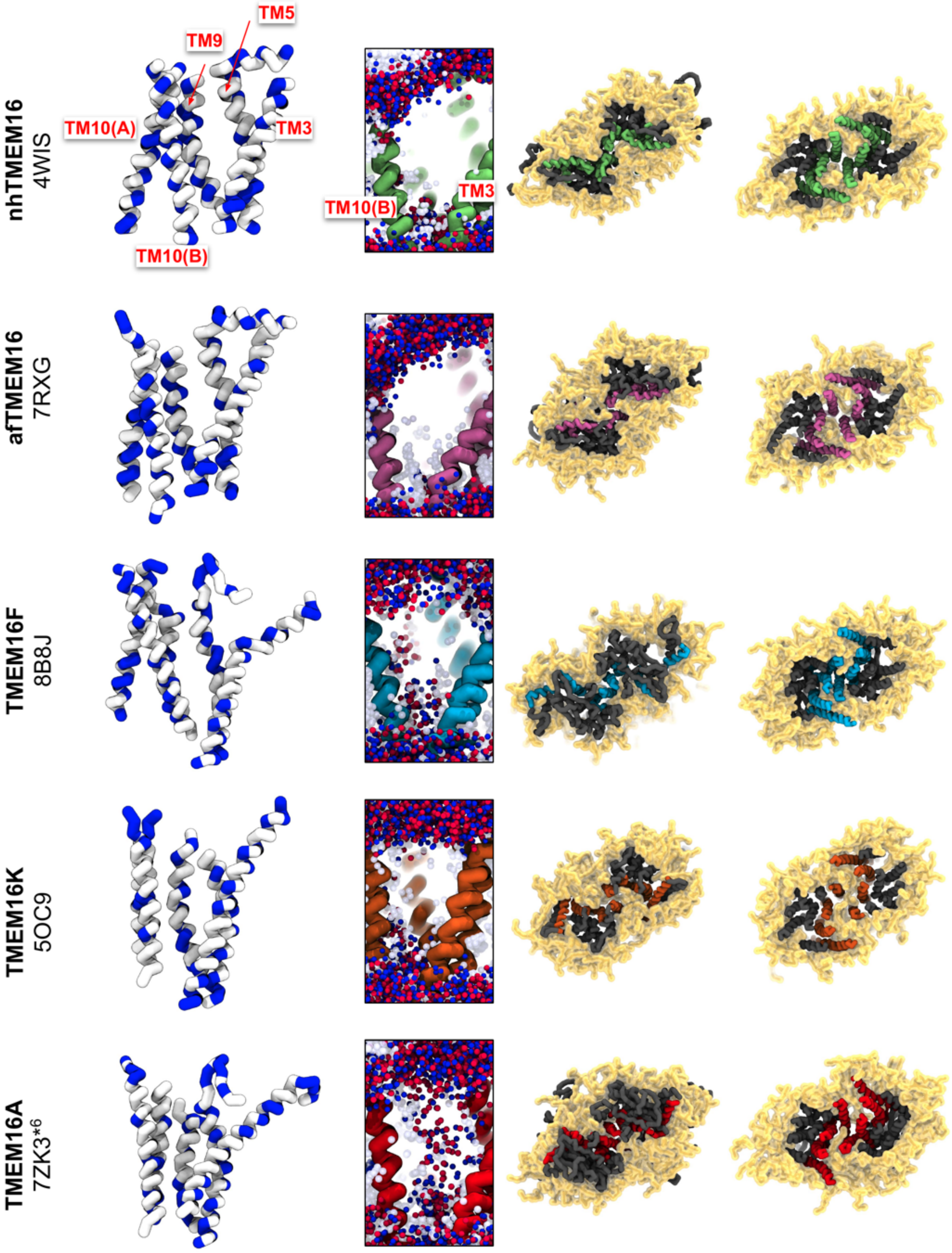

Figure S24. Dimer interface hydrophobicity and lipid positions. Images in each row were taken from CG MD simulations of 5 different TMEM16s. The first column depicts TM helices forming one half of the dimer interface (rest of the protein not shown) colored by residue type (small/hydrophobic: white, charge/polar: blue). The second column depicts snapshots of the same dimer interface helices with overlayed positions of water (light blue spheres) and lipid head groups (red and blue spheres) every 100 frames of the last 9 μs of each simulation. The last two columns are two views of the same snapshot showing the protein with its annulus of lipids (yellow). The dimer interface-forming helices are colored green (nhTMEM16), purple (afTMEM16), blue (TMEM16F), orange (TMEM16K), and red (TMEM16A).

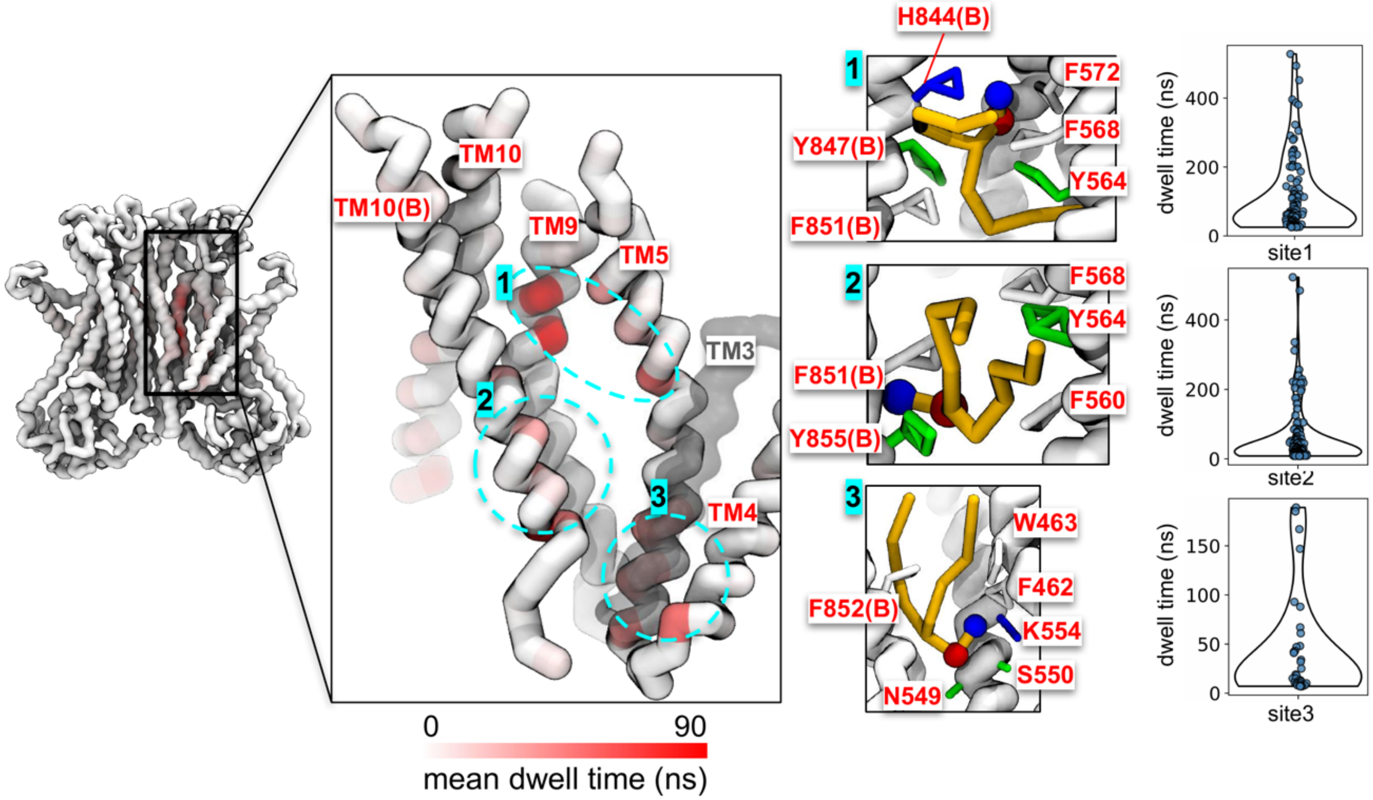

Figure S25. Dwell time analysis for scrambling events observed at TMEM16F F518H mutant dimer interface. The scrambling region is delineated by TM3, TM4, TM5, TM9, and TM10 both monomers. TM3 and part of TM5 are transparent for clarity. The backbone region of the dimer interface is colored by dwell time of the scrambling lipids. Sites with prolonged dwell time are circled in cyan (center left) and shown in zoomed-in images (center right). Distributions of dwell times at each site are shown as violin plots (far right).

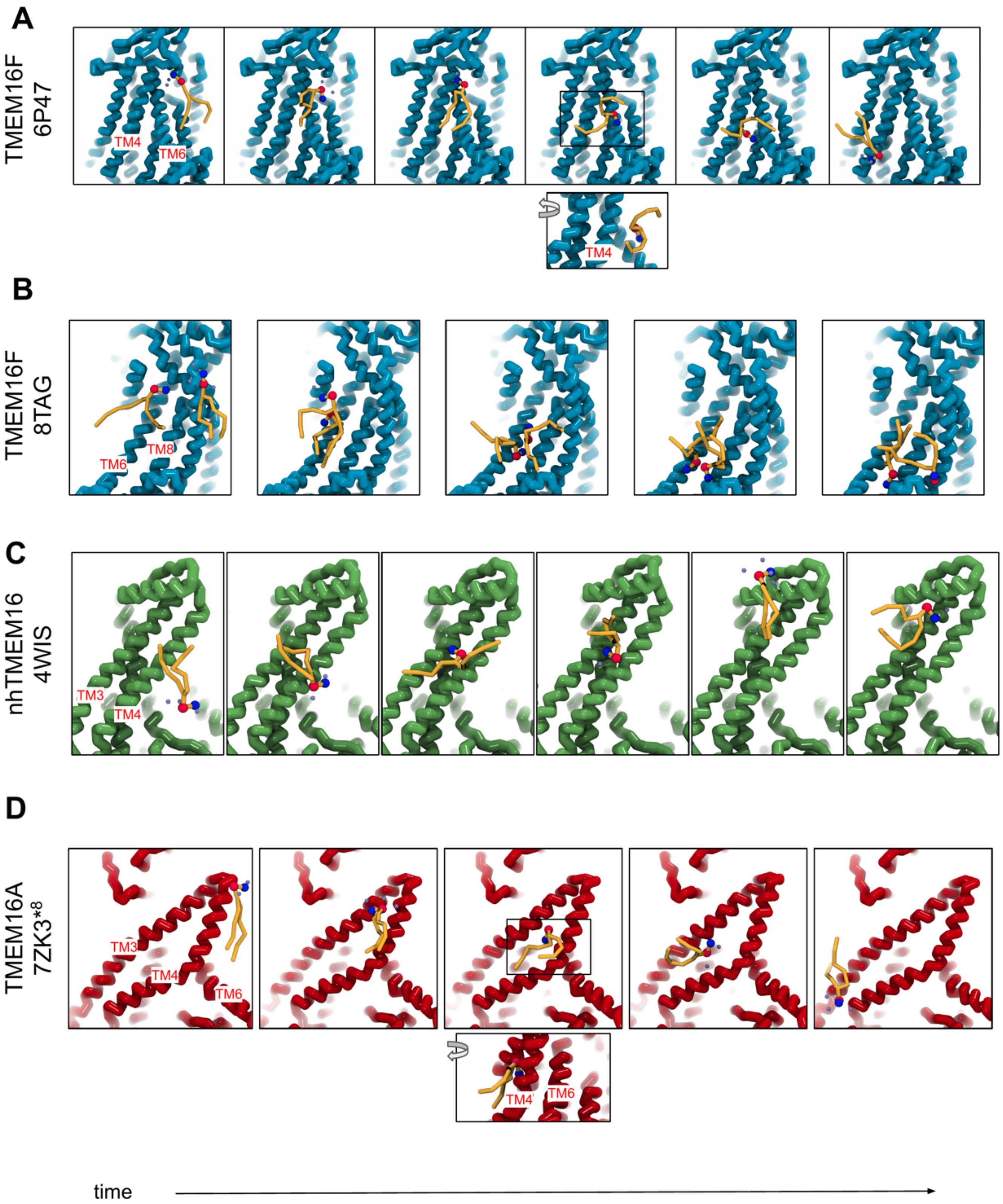

Figure S26. "Out-of-the-groove" scrambling events outside of the dimer interface. (A) Snapshots over time of a single lipid scrambling event by TMEM6F over the closed TM4/TM6 groove. **(B)** Snapshots over time of two concurrent scrambling events by TMEM16F over TM6 and TM8. **(C)** Snapshots over time of a single lipid scrambling event by nhTMEM6 over TM3 and TM4. **(D)** Snapshots over time of a single lipid scrambling event by TMEM6A over TM3 and TM4.

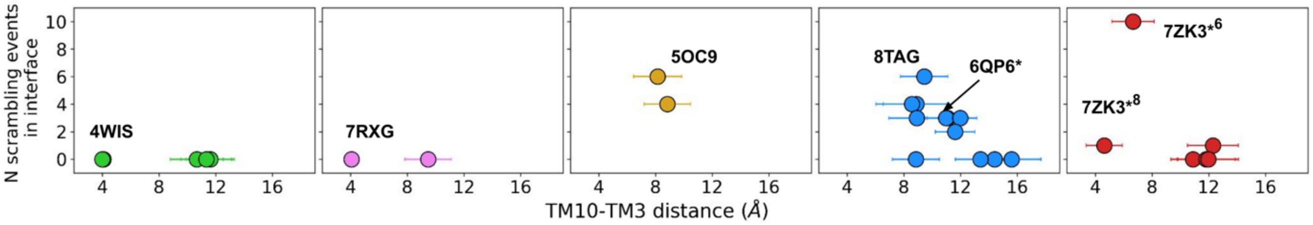

Figure S27. Number of scrambling events and the width of the dimer interface inner leaflet entrance. The number of scrambling events at the dimer interface plotted against the average minimum distance between TM10 and TM3 on the opposite helix near the inner leaflet-solvent boundary.

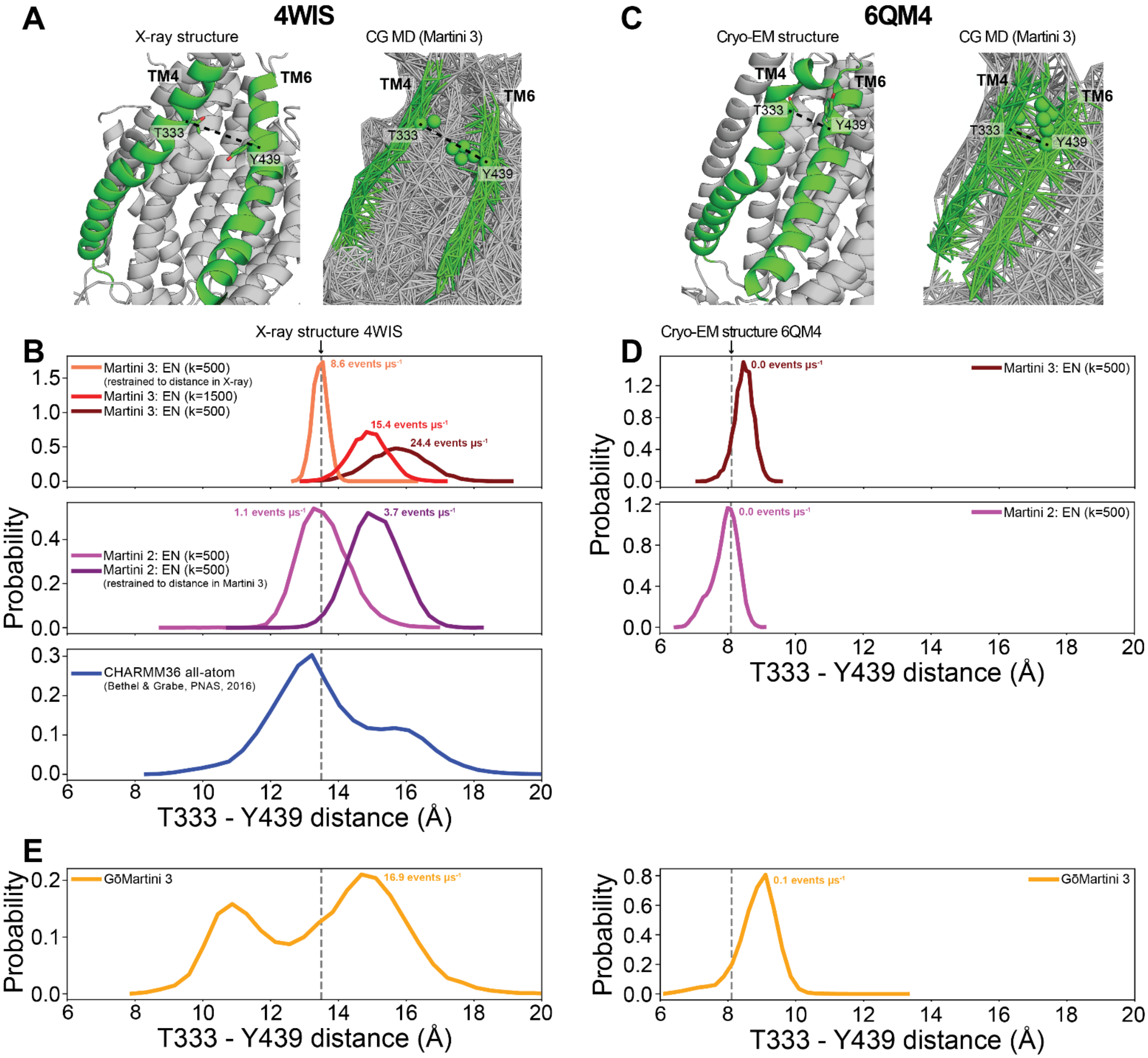

Figure S28. TM4/TM6 groove distances vary between different force fields. (A) The X-ray structure (left) and the full elastic network for the last frame of our CG MD Martini 3 simulation (right) of open-groove nhTMEM16 (4WIS). TM4 and TM6 are colored green. The dashed line indicates the distance between the C_α-atoms/backbone beads of T333 and Y439, which is used to measure TM4/TM6 distance in **B** and **E**. **(B)** Probability distributions of the T333-Y439 distance in 4WIS for different settings of the Martini 3, Martini 2, and CHARMM36 force fields. EN = elastic network. k indicates the force constant used in the EN. Scrambling rates are calculated as described throughout the current work. The CHARMM36 data was taken from ref. (6). Dashed gray line indicates the distance in the X-ray structure. **(C)** The cryo-EM structure (left) and the full elastic network for the last frame of our CG MD Martini 3 simulation (right) of closed-groove nhTMEM16 (6QM4). TM4 and TM6 are colored green. The dashed line indicates the distance between the C_α-atoms/backbone beads of T333 and Y439, which is used to measure TM4/TM6 distance in **D-E**. Note the elastic network connections between TM4 and TM6 in green, that are absent in **A**. **(D)** Probability distributions of the T333-Y439 distance in 6QM4 for different settings of the Martini 3 and Martini 2 force fields. Dashed gray line indicates the distance in the X-ray structure. **(E)** Probability distributions of the T333-Y439 distance in 4WIS (left) and 6QM4 (right) for simulations with the GōMartini 3 force field (7). Dashed gray line indicates the distance in the respective experimental structures.

	PDB ID	Mutations	Source	# calcium	State	Scrambling events/µs	std	Thinning (Å)	mean TM4/6 distance (Å)	std	mean TM10/3 distance (Å)	std	Water permeation/µs	std	# Cl- permetations	# Na+ permeations	selectivity
nhTMEM16	4WIS	WT	X-ray	4	Open	24.44	5.23	12.0	7.85	0.17	4.08	0.21	545.40	32.90	10.00	51.00	5.10
	6QM6	WT	Cryo-EM	0	Open	15.67	3.94	11.6	7.49	0.2	4	0.25	369.50	31.70	5.00	16.00	3.20
	6QM4	WT	Cryo-EM	0	Closed	0.00	0.00	22.4	3.53	0.01	10.66	1.86	55.50	6.10	0.00	0.00	1.00
	6QMA	WT	Cryo-EM	4	Intermediate	0.00	0.00	18.5	4.67	0.27	11.61	1.7	161.00	22.80	0.00	0.00	1.00
	6QMB	WT	Cryo-EM	4	Closed	0.00	0.00	22.1	3.55	0.01	11.33	1.78	98.30	13.00	0.00	0.00	1.00
afTMEM16	7RXG	WT	Cryo-EM	4	Open	10.67	2.91	12.2	7.57	0.2	4.07	0.3	353.70	47.10	1.00	6.00	6.00
	7RXB	WT	Cryo-EM	0	Closed	0.00	0.00	12.2	3.55	0	9.46	1.64	122.50	17.00	0.00	0.00	1.00
TMEM16K	5OC9	WT	X-ray	6	Open	8.22	2.94	11.9	8.23	1.28	8.15	1.69	314.80	80.20	5.00	9.00	1.80
	6R7X	WT	Cryo-EM	6	Closed	0.44	0.68	19.1	3.88	0.03	8.83	1.63	33.60	10.50	0.00	0.00	1.00
TMEM16F	8B8J	F518H	Cryo-EM	6	Open, kink in TM3	11.33	4.08	11.9	5.14	0.47	8.88	2.33	546.80	56.60	43.00	56.00	1.30
	6QP6*(10)	WT	Simulation	6	One open groove, one closed groove	3.00	1.56	13.1	5.55	0.16	11.07	1.43	411.10	48.30	9.00	3.00	0.33
	8TAG	WT	Cryo-EM	3	Closed	1.00	0.67	14.1	3.63	0.01	9.43	1.67	44.40	13.80	0.00	0.00	1.00
	8B8Q	F518H	Cryo-EM	6	Closed	0.44	0.68	17.7	3.55	0.02	10.97	1.58	218.00	22	0	0	1.00
	6P47	WT	Cryo-EM	0	Closed	0.44	0.50	18.3	3.65	0.02	11.98	1.17	45.70	13.10	0.00	0.00	1.00
	6P48	WT	Cryo-EM	2	Closed	0.44	0.50	18.9	3.60	0.02	8.57	2.53	3.70	2.20	0.00	0.00	1.00
	8BC0	F518A, Q623A	Cryo-EM	6	Closed	0.44	0.50	19.0	3.48	0.02	8.92	1.99	49.90	10.50	0.00	0.00	1.00
	6QPB	WT	Cryo-EM	0	Closed	0.33	0.67	16.3	3.70	0.03	11.61	1.39	69.90	9.50	0.00	0.00	1.00
	8B8G	F518H	Cryo-EM	0	Closed	0.22	0.42	13.8	3.70	0.01	15.6	2.06	24.90	6.20	0.00	0.00	1.00
	6QP6	WT	Cryo-EM	6	Closed	0.00	0.00	15.7	3.65	0.02	14.4	0.9	39.90	5.50	0.00	0.00	1.00
	6QPC	WT	Cryo-EM	4	Closed	0.00	0.00	17.2	3.62	0.01	8.85	1.65	12.60	6.20	0.00	0.00	1.00
	8B8K	N562A	Cryo-EM	6	Closed	0.00	0.00	17.5	3.72	0.01	13.41	1.77	47.30	11.70	0.00	0.00	1.00
TMEM16A	7ZK3*(6)	WT	Simulation	6	Closed (conductive to ions)	1.22	1.55	19.6	4.41	0.04	6.66	1.49	77.90	15.30	0.00	0.00	1.00
	5OYB*	WT	Simulation	4	Closed (conductive to ions)	0.22	0.42	16.7	5.13	0.16	10.88	1.55	168.80	46.70	0.00	0.00	1.00
	7ZK3*(8)	WT	Simulation	6	Closed (conductive to ions)	0.11	0.31	12.5	4.59	0.06	4.63	1.27	372.30	38.80	4.00	0.00	-
	7ZK3*(10)	WT	Simulation	6	Closed (conductive to ions)	0.11	0.31	16.6	4.21	0.02	12.28	1.79	246.00	40.00	0.00	0.00	1.00
	5OYG	WT	Cryo-EM	0	Closed	0.00	0.00	23.1	4.30	0.01	11.82	2.04	48.80	19.50	0.00	0.00	1.00
	5OYB	WT	Cryo-EM	4	Closed	0.00	0.00	23.1	4.16	0.02	11.96	2.12	56.70	40.80	0.00	0.00	1.00

Table S2. Values for calculated membrane thinning, water permeation, and TM helix distances.

REFERENCES

1. V. Kalienkova, *et al.*, Stepwise activation mechanism of the scramblase nhtmem16 revealed by cryo- em. *Elife* **8** (2019).
2. M. E. Falzone, *et al.*, TMEM16 scramblases thin the membrane to enable lipid scrambling. *Nat Commun* **13**, 2604 (2022).
3. S. R. Bushell, *et al.*, The structural basis of lipid scrambling and inactivation in the endoplasmic reticulum scramblase TMEM16K. *Nat Commun* **10**, 3956 (2019).
4. M. Arndt, *et al.*, Structural basis for the activation of the lipid scramblase TMEM16F. *Nat Commun* **13**, 6692 (2022).
5. Schrödinger LLC, "The AxPyMOL Molecular Graphics Plugin for Microsoft PowerPoint, Version~1.8" (2015).
6. N. P. P. Bethel, M. Grabe, Atomistic insight into lipid translocation by a TMEM16 scramblase. *Proceedings of the National Academy of Sciences* **113**, 14049–14054 (2016).
7. P. C. T. Souza, *et al.*, GōMartini 3: From large conformational changes in proteins to environmental bias corrections. *bioRxiv* 2024.04.15.589479 (2024). https://doi.org/10.1101/2024.04.15.589479.
